# Production of tailored hydroxylated prodiginine showing combinatorial activity with rhamnolipids against plant-parasitic nematodes

**DOI:** 10.3389/fmicb.2023.1151882

**Published:** 2023-05-02

**Authors:** D. F. Kossmann, M. Huang, R. Weihmann, X. Xiao, F. Gätgens, T. M. Weber, H. U. C. Brass, N. L. Bitzenhofer, S. Ibrahim, K. Bangert, L. Rehling, C. Mueller, T. Tiso, L. M. Blank, T. Drepper, K.-E. Jaeger, F. M. W. Grundler, J. Pietruszka, A. S. S. Schleker, A. Loeschcke

**Affiliations:** ^1^Institute of Bio- and Geosciences (IBG-1): Biotechnology, Forschungszentrum Jülich GmbH, Jülich, Germany; ^2^Institute of Bioorganic Chemistry, Forschungszentrum Jülich, Heinrich Heine University Düsseldorf, Jülich, Germany; ^3^INRES, Molecular Phytomedicine, University of Bonn, Bonn, Germany; ^4^Institute of Molecular Enzyme Technology, Forschungszentrum Jülich, Heinrich Heine University Düsseldorf, Jülich, Germany; ^5^iAMB—Institute of Applied Microbiology, ABBt—Aachen Biology and Biotechnology, RWTH Aachen University, Aachen, Germany

**Keywords:** Prodiginines, plant-parasitic nematodes, plant protection, mutasynthesis and semisynthesis, *Pseudomonas putida*, combinatorial activity, rhamnolipids

## Abstract

Bacterial secondary metabolites exhibit diverse remarkable bioactivities and are thus the subject of study for different applications. Recently, the individual effectiveness of tripyrrolic prodiginines and rhamnolipids against the plant-parasitic nematode *Heterodera schachtii*, which causes tremendous losses in crop plants, was described. Notably, rhamnolipid production in engineered *Pseudomonas putida* strains has already reached industrial implementation. However, the non-natural hydroxyl-decorated prodiginines, which are of particular interest in this study due to a previously described particularly good plant compatibility and low toxicity, are not as readily accessible. In the present study, a new effective hybrid synthetic route was established. This included the engineering of a novel *P. putida* strain to provide enhanced levels of a bipyrrole precursor and an optimization of mutasynthesis, i.e., the conversion of chemically synthesized and supplemented monopyrroles to tripyrrolic compounds. Subsequent semisynthesis provided the hydroxylated prodiginine. The prodiginines caused reduced infectiousness of *H. schachtii* for *Arabidopsis thaliana* plants resulting from impaired motility and stylet thrusting, providing the first insights on the mode of action in this context. Furthermore, the combined application with rhamnolipids was assessed for the first time and found to be more effective against nematode parasitism than the individual compounds. To obtain, for instance, 50% nematode control, it was sufficient to apply 7.8 μM hydroxylated prodiginine together with 0.7 μg/ml (~ 1.1 μM) di-rhamnolipids, which corresponded to *ca.* ¼ of the individual EC_50_ values. In summary, a hybrid synthetic route toward a hydroxylated prodiginine was established and its effects and combinatorial activity with rhamnolipids on plant-parasitic nematode *H. schachtii* are presented, demonstrating potential application as antinematodal agents.

**Graphical Abstract:**
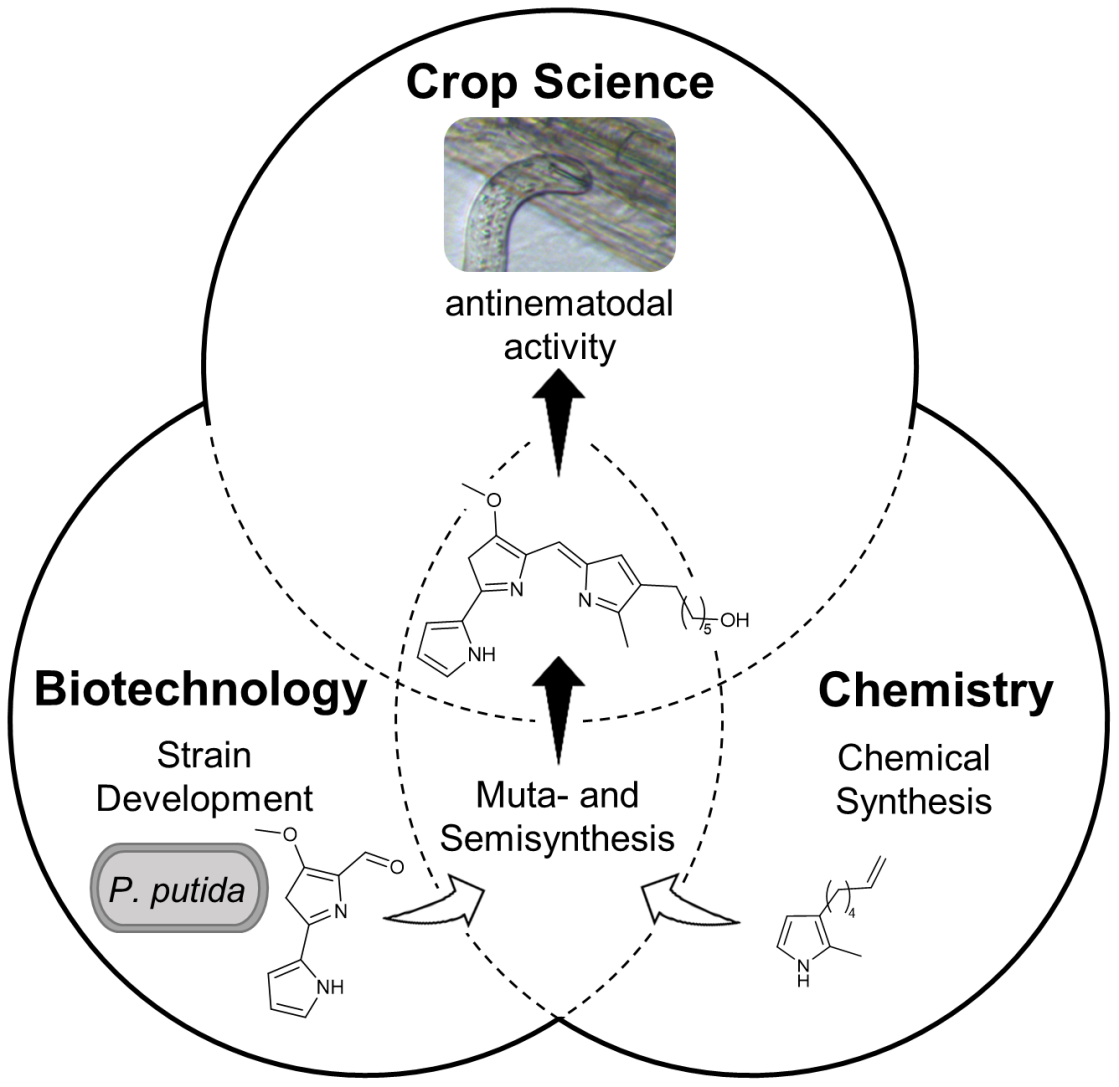

## 1. Introduction

Prodiginines are a group of bacterial secondary metabolites with diverse remarkable bioactivities, among which anticancer and antimicrobial effects have perhaps been described in most detail thus far (Fürstner, 2003; Stankovic et al., 2014; Hu et al., 2016; Sakai-Kawada et al., 2019; Yip et al., 2019; Berning et al., 2021; Li et al., 2022). Notably, prodiginine-producing bacteria such as *Streptomyces* and *Serratia* species also dwell in the rhizosphere, the dynamic micro-biosphere around the plant roots and site of manifold chemical interactions between the bacteria and plants (Berg, 2009; Lugtenberg and Kamilova, 2009; Meschke et al., 2012). Here, direct interactions can result in hormonal stimulation, increased stress tolerance and improved nutrient availability and uptake by plant roots. In addition, indirect beneficial effects for the plant can result from the suppression of pathogens. Although the ecophysiological role of prodiginines is still poorly understood, various bioactivities of prodigiosin (**1**) and related tripyrrolic analogs against several plant pathogens have already been reported (Someya et al., 2001; Meschke et al., 2012; Rahul et al., 2014; Roberts et al., 2021). Recently, their effectiveness against the plant-parasitic nematode (PPN) *Heterodera schachtii*, the causative agent of tremendous losses in crop plants, was described (Habash et al., 2020). Moreover, naturally-occurring bacterial secondary metabolites with surfactant properties, namely rhamnolipids, were recently reported to act against *H. schachtii* (Bredenbruch et al., 2023). It has further been shown that prodigiosin (**1**) possesses combinatorial antibacterial activities when applied together with biosurfactants, such as the lipopeptide serrawettin W1 and rhamnolipids (Hage-Hülsmann et al., 2018). It has been postulated that the pigment can only exert its full antimicrobial activity in combination with biosurfactants (Williamson et al., 2008; Roberts et al., 2021).

The aim of the present interdisciplinary study was to facilitate access to the relevant compounds and to investigate their combinatorial activities against *H. schachtii*. *Pseudomonas putida* has become a widely established biotechnological host for natural product biosynthesis (Nikel et al., 2016; Loeschcke and Thies, 2020; Weimer et al., 2020) including heterologous prodigiosin and rhamnolipid production (Domröse et al., 2015; Tiso et al., 2020; Cook et al., 2021). The rhamnolipid bioprocess has already been industrially implemented by Evonik Industries AG while research to improve accessing prodiginine derivatives is ongoing: recent studies showed heterologous expression of the *pig* gene cluster of *Serratia marcescens* via chromosomal integration, which established the biosynthesis of prodigiosin (**1**) (Domröse et al., 2015, 2019; Cook et al., 2021). Further, a mutasynthesis approach enabled the generation of new prodiginines (Klein et al., 2017, 2018). This procedure was based on the partial disruption of the native biosynthesis pathway and feeding of monopyrrole precursor analogs, which were incorporated into new tripyrrolic compounds. A limitation of this mutasynthesis approach was a relatively low product yield of 2–12% (Klein et al., 2017). Therefore, the development of a novel mutasynthesis *chassis* with enhanced bipyrrole production capacity and an optimization of mutasynthesis were identified as promising strategies to obtain target prodiginines more efficiently.

**Figure 1:**
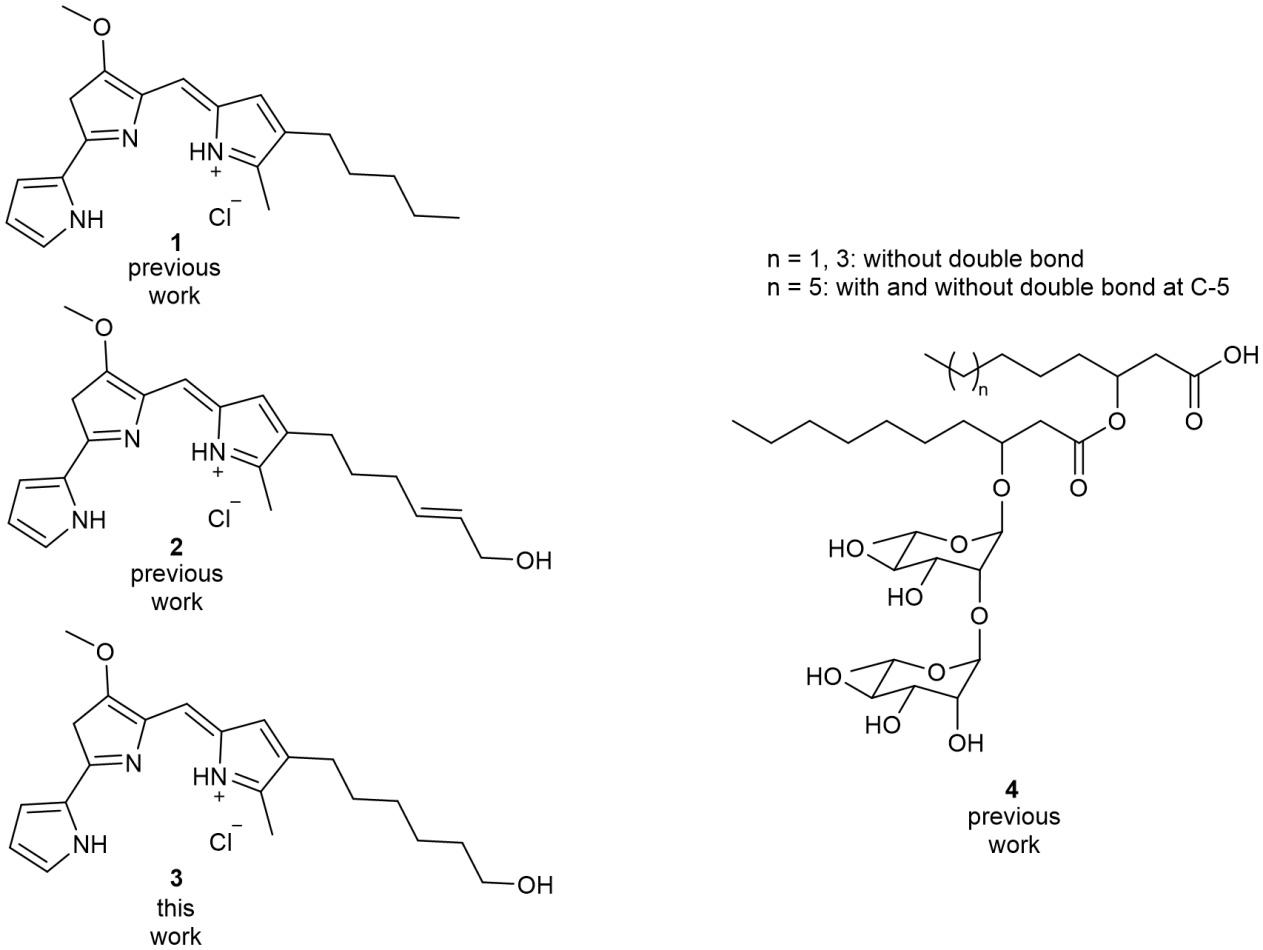
Bioactive compounds assessed in this study. The synthesis of prodigiosin **(1)** and hydroxylated derivative **(2)** has been established in previous studies. A synthesis route to hydroxylated prodiginine **(3)** is presented in this study, enabling the compound’s application together with di-rhamnolipids **(4)**, which can be obtained via microbial biosynthesis as a mixture of congeners as indicated.

In previous work, a prodiginine bearing a terminal allylalcohol group (**2**) was reported to show remarkable plant growth promoting properties. In addition, it was not toxic for *Caenorhabditis elegans* at concentrations where prodigiosin (**1**) was lethal for this non-target organism (Habash et al., 2020). Based on these findings, the focus of the present study is the development of a feasible synthetic approach to hydroxylated prodiginines for investigations of anti-nematode activity. This study thus reports a mutasynthesis approach in a novel, efficient bipyrrole-producing *P. putida* strain and subsequent chemical conversion, i.e., semisynthesis, toward hydroxylated prodiginine **3** (Figure 1). The mode of action against *H. schachtii* as well as combinatorial activity with di-rhamnolipids (**4**) were investigated for the first time, demonstrating potential application as antinematodal agents.

## 2. Results

### 2.1. Development of an enhanced *Pseudomonas putida chassis* for optimized mutasynthesis

In natural prodigiosin (**1**) biosynthesis, the monopyrrole MAP (2-methyl-3-amylpyrrole, **5**) and the bipyrrole MBC (4-methoxy-2,2′-bipyrrole-5-carbaldehyde, **6**) are condensed by a ligase (PigC in *S. marcescens*) to a tripyrrolic scaffold. The mutasynthesis approach has previously been established successfully in *P. putida* pig-r2 Δ*pigD* (Klein et al., 2017, 2018). The strain harbored the entire *S. marcescens pig* gene cluster—with the exception of the first MAP biosynthetic gene *pigD*, which was replaced by an antibiotic resistance gene. By feeding chemically synthesized monopyrroles to this MAP-deficient *P. putida* pig-r2 Δ*pigD* strain, the PigC-catalyzed condensation with MBC (**6**) led to novel tripyrrolic compounds in these previous studies.

**Figure 2:**
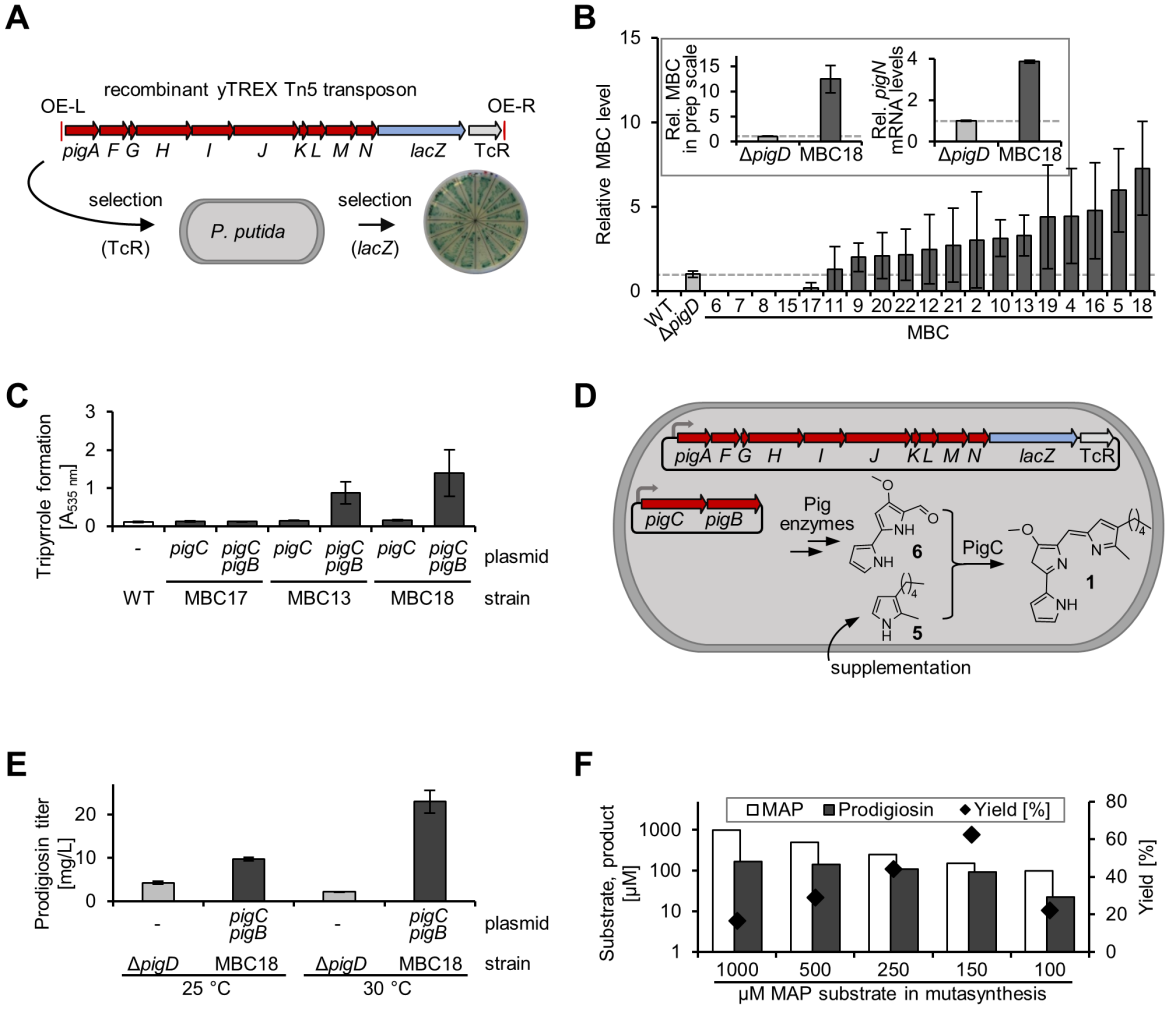
Construction of a *Pseudomonas putida* mutasynthesis *chassis*. **(A)** The artificial 4-methoxy-2,2′-bipyrrole-5-carbaldehyde (MBC) biosynthesis-encoding gene cluster was assembled by yeast-cloning and integrated in the bacterial chromosome via Tn5 transposition. After that, expressing clones were selected based on LacZ reporter activity. **(B)** Relative MBC production in small scale cultivation of engineered *P. putida* shown as x-fold increase relative to the signal of *P. putida* pig-r2 Δ*pigD*. Left Inset: Evaluation in preparative mutasynthesis setup (100 ml scale, MBC18 with pVLT33-pigC-pigB). Right inset: Relative *pigN* mRNA levels. **(C)** Dependence of the condensation of biosynthesized MBC (**6**) with supplemented MAP (**5**) on co-expression of PigB in *P. putida* stains MBC17, −13, −18 with plasmids pVLT33-pigC or pVLT33-pigC-pigB, respectively, in small scale cultivation. **(D)** Genetic setup of the new mutasynthesis *chassis* carrying the MBC (**6**) biosynthetic genes in the chromosome and expressing *pigC* and *pigB* from a plasmid. **(E)** Mutasynthesis performance of the newly established *chassis P. putida* MBC18/pVLT33-pigC-pigB in comparison with hitherto used *P. putida* pig-r2 Δ*pigD* in small scale cultivation at 25 or 30°C. The product was detected in extracts via spectrophotometry. Shown are mean values of independently replicated measurements (min. triplicates) with the respective standard deviation. **(F)** Optimization of preparative mutasynthesis in 5 × 100 ml scale with *P. putida* MBC18/pVLT33-pigC-pigB. The amount of substrate MAP (**5**) was varied from 1,000 μM to 100 μM to optimize conditions with respect to isolated prodigiosin (**1**) and corresponding yields (% mol of product relative to the substance amount of substrate).

Since mutasynthesis might be limited by biosynthetic provision of the bipyrrole precursor MBC (**6**), we aimed to establish a streamlined MBC-producing *chassis* preferentially lacking genetic and metabolic burdens. As a new strategy, a truncated *pig* gene cluster specifically designed to facilitate the biosynthesis of the bipyrrole **6** was integrated in the host. To this end, the artificial *pigAFGHIJKLMN* gene cluster (excluding the MAP (**5**) biosynthesis-encoding genes *pigBDE* and the ligase-encoding *pigC*, see Supplementary Figure S1) was assembled by PCR and yeast recombinational cloning into the yTREX vector following established protocols (Domröse et al., 2017; Weihmann et al., 2020). In this process, the promotor-less *lacZ* gene was inserted downstream of *pigN* (Figure 2A). The resulting vector was used for random transposon Tn5-based integration of the recombinant operon in the *P. putida* bacterial chromosome and LacZ was utilized as transcription reporter. Based on that, clones with strong gene expression were indicated by blue coloration as a result of X-gal conversion. Of these clones, 19 were selected and subjected to small scale cultivation, metabolite extraction and LC–MS analysis to verify bipyrrole product accumulation. Results were comparatively evaluated in relation to the previously established mutasynthesis *chassis P. putida* pig-r2 Δ*pigD* (Figure 2B). While four strains did not produce any detectable amounts of MBC (**6**), one clone (MBC17) produced a lower amount of MBC (**6**) than *P. putida* pig-r2 Δ*pigD*, and 14 accumulated the bipyrrole at higher levels (up to 8-fold increased). The best producer (MBC18) showed likewise elevated transcript levels (approx. 4-fold), indicating that a higher expression level could be reached in the new strain, which in turn contributed to higher bipyrrole synthesis. The enhanced MBC (**6**) level was verified for MBC18 in larger scale as applied in preparative mutasynthesis: Both strains, *P. putida* pig-r2 Δ*pigD* and MBC18, were cultivated in TB medium and polyurethane (PU) foam cubes were added as adsorbent for the hydrophobic compound, as previously established for prodigiosin (**1**) recovery (Domröse et al., 2015). Extracts from PU of the cultures with the newly constructed strain MBC18 contained about 12-fold more MBC (**6**) than those of the previously reported strain *P. putida* pig-r2 Δ*pigD* — a promising precondition for mutasynthesis.

The next step was to evaluate the capacity of the new strains for mutasynthesis with MBC17, MBC13, and MBC18 representing a low, an intermediate, and a high MBC level producer, respectively. The ligase PigC was thus introduced into these strains by plasmid-based expression from the IPTG-inducible P*_tac_* promoter in pVLT33-pigC (Brands et al., 2020). After small scale cultivation with supplementation of MAP (**5**, 1 mm), cells were extracted and prodigiosin-specific absorption was measured at 535 nm (Figure 2C). However, no noteworthy conversion of the fed monopyrrole to prodigiosin could be detected with either strain (MBC17, MBC13, MBC18/*pigC*).

Since it is known that in *in vitro* assays PigC does not require any additional proteins for functioning (Brass et al., 2019; Brands et al., 2020) it could be excluded that a generally necessary component was missing in the new strains. However, the previous *pigD* deletion mutant *P. putida* pig-r2 Δ*pigD* could facilitate conversion and it additionally contained functional PigB and PigE-encoding genes. This suggested an important role of either one or both of these proteins in the mutasynthesis setup (see Supplementary Figure S1). Based on their localization at the membrane and the proven importance of their membrane-anchoring domains, it may further be speculated that the last precursor-delivering enzymes PigN/F and PigB, as well as PigC could form a membrane-associated protein complex (Williamson et al., 2005; Chawrai et al., 2012; Couturier et al., 2019). This suggested that this complex consisting of PigC and PigN/F, but devoid of PigB might not be functional (see also Supplementary Figures S2, S3). PigB co-expression was therefore tested next by using plasmid pVLT33-pigC-pigB in the new strains (Figures 2C,D). While in the low-level precursor providing strain MBC17, co-expression of PigB did not lead to prodigiosin (**1**) formation, an increased signal was observed with MBC13 and highest levels were found in the high-level MBC-providing strain MBC18 (MBC17, MBC13, MBC18/*pigC-pigB*).

As a next step, mutasynthetic prodigiosin (**1**) production of the newly established *chassis P. putida* MBC18/pVLT33-pigC-pigB was assessed in comparison with hitherto used *P. putida* pig-r2 Δ*pigD*. To this end, small scale cultivation was used to test performance at 25°C as previously established (Klein et al., 2017, 2018) and at the optimal *P. putida* growth temperature 30°C (Figure 2E). At 25°C, 4.2 and 10 mg/L prodigiosin were obtained, while at 30°C, 2.3 and 21 mg/L were produced with *P. putida* pig-r2 Δ*pigD* and MBC18/pVLT33-pigC-pigB, respectively. These results therefore verified enhanced performance of the newly constructed *chassis*, especially at 30°C, so all following experiments were conducted at this temperature.

Since during small scale cultivation under the applied conditions with excess of MAP (1 mm, **5**), MBC (**6**) was consumed to below limits of quantification, an adjustment of MAP concentrations was tested next. Hence, different MAP (**5**) concentrations were supplemented to *P. putida* MBC18/pVLT33-pigC-pigB in order to potentially match the levels of both precursors in preparative scale experiments and optimize yields (Figure 2F). The best yield of 62% purified product was obtained with 150 μM MAP (**5**). Isolation by soxhlet extraction of PU and column chromatography on silica yielded 17 mg prodigiosin (**1**) from 500 ml mutasynthesis cultures. This outcome corresponding to 34 mg/l represented an improvement to previously reported 17 mg/l, obtained with the same pyrrole via comparable procedures (Klein et al., 2017). Remaining MBC (**6**) was below 2 μM in this experiment (Supplementary Figure S4). This procedure was therefore deemed as suitable for further steps toward accessing the hydroxylated target compound **3**.

### 2.2. Chemical synthesis of pyrroles, muta- and semisynthesis of hydroxylated prodiginine **3**

In order to establish a synthesis route to a hydroxylated prodiginine tripyrrole, a pyrrole with terminal double bond was aimed as precursor to allow a late-stage semisynthetic functionalization. Previously reported prodiginine **2** was synthesized chemically in a condensation reaction of *Boc*-**6** and a pyrrole with allylalcohol function (Habash et al., 2020). With this approach, the functional group is already applied, but further modification and derivatization are less feasible. The here presented mutasynthesis approach is more flexible in terms of late-stage functionalization and allows to functionalize the obtained mutasynthesis product in a variety of ways, such as with a bromination or oxidation. In order to yield the hydroxylated prodiginine **3**, we applied a hydroboration as semisynthetic step.

**Figure 3:**
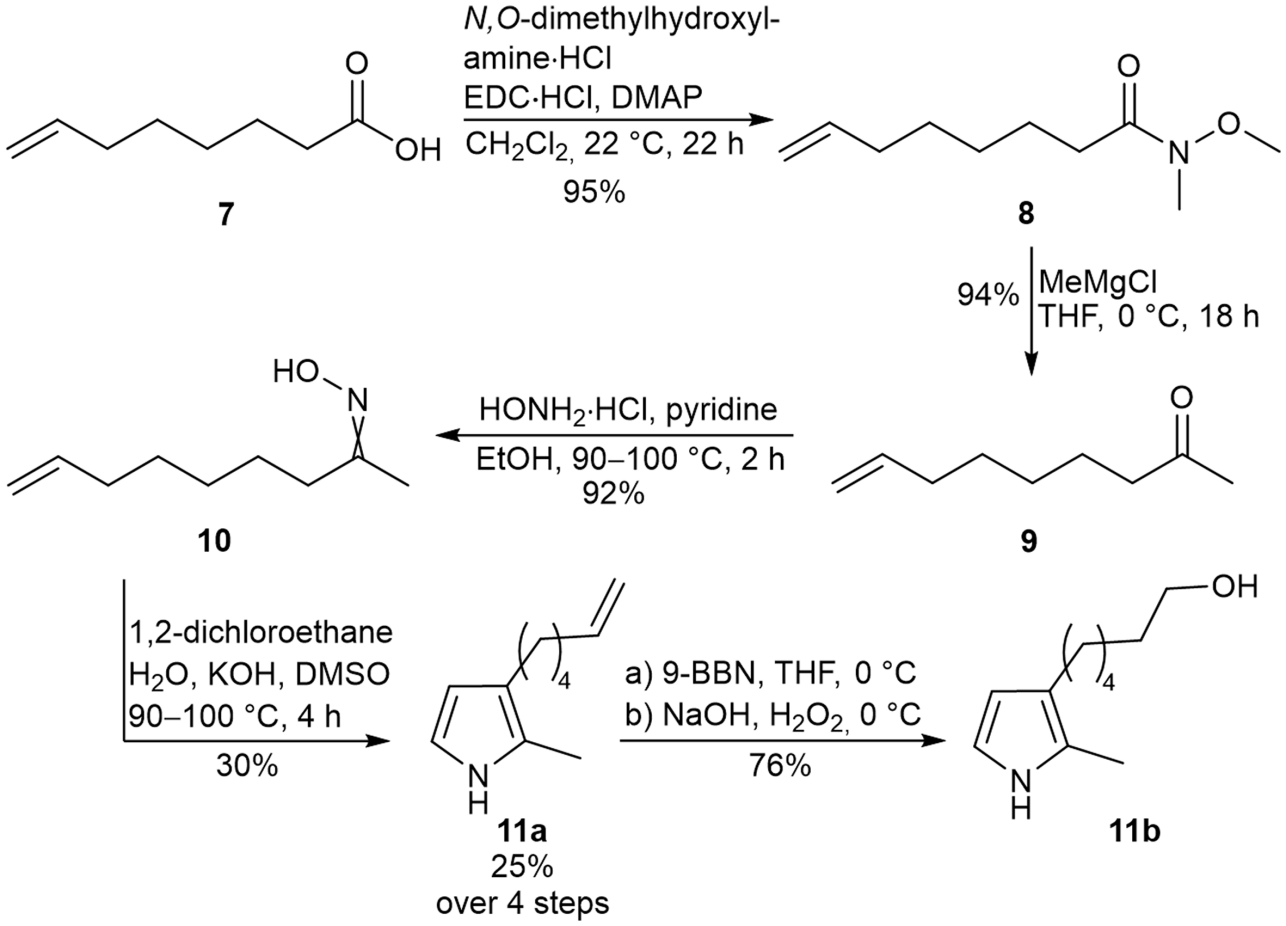
Synthesis route toward the 2,3-disubstituted pyrrole precursors **11a** and **11b**.

Starting from carboxylic acid **7**, Weinreb-amide **8** and ketone **9** were obtained in two steps, consecutively. Afterwards, oxime **10** was synthesized to be used as starting material in the Trofimov pyrrole synthesis toward pyrrole **11a**, as previously described (Mikhaleva et al., 1981; Trofimov et al., 2015; Klein et al., 2018). The Trofimov pyrrole synthesis represents the yield-limiting step in this synthesis sequence (Figure 3). This could be explained by the strongly basic reaction conditions, which favor side-product formations as described previously (Ivanov et al., 2014). Observed side-products were a ketoxime diether and an *N*-alkylated pyrrole, which could be removed by flash column chromatography on silica. In a subsequent hydroboration with the organoborane 9-BBN (9-Borabicyclo[3.3.1]nonane), a hydroxylated pyrrole **11b** was obtained as precursor.

**Figure 4:**
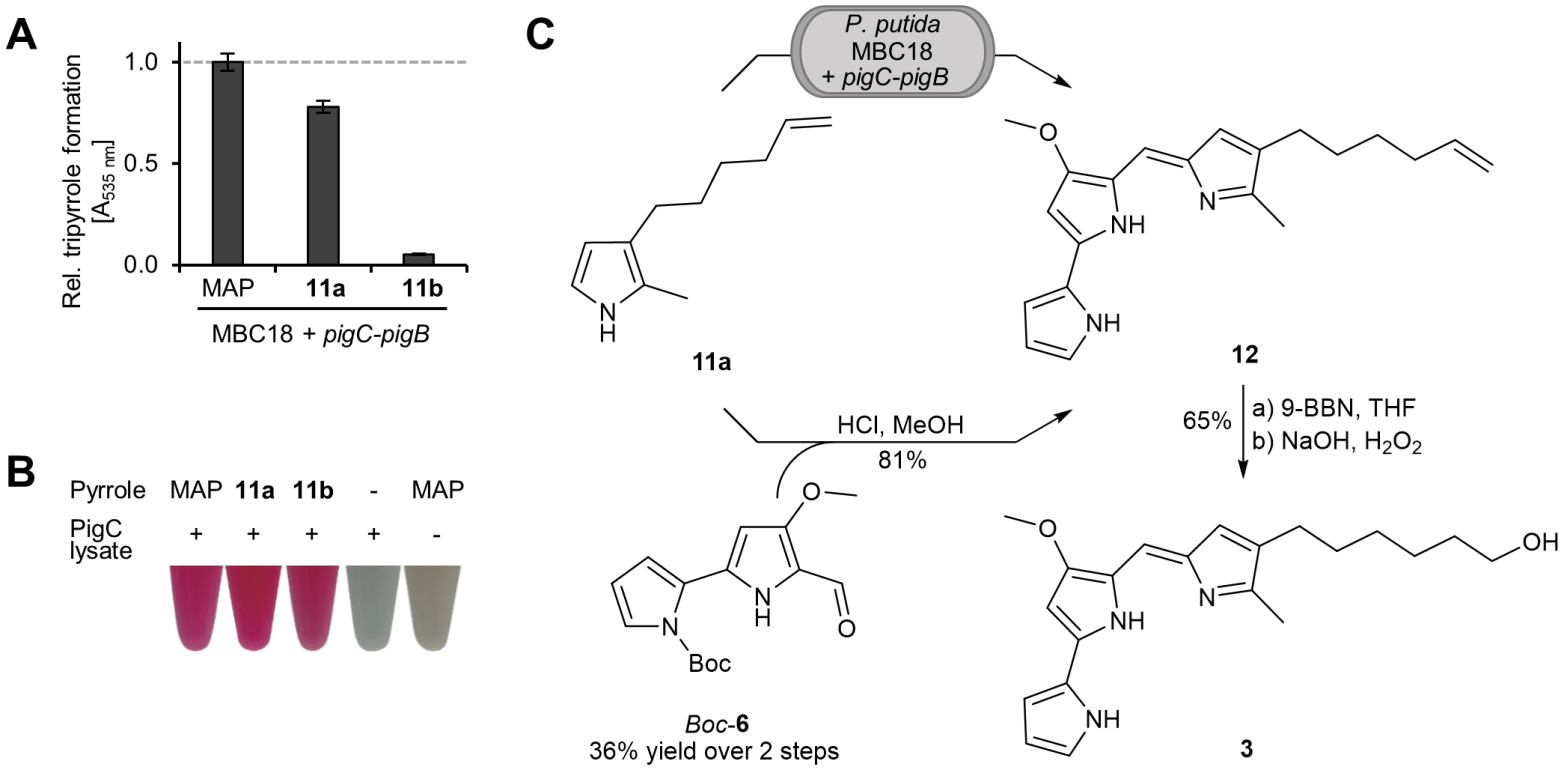
Synthetic route toward hydroxylated prodiginine 3. **(A)** Mutasynthetic tripyrrole formation upon feeding of **11a** and **11b**, respectively, in small scale cultivation of *P. putida* MBC18/pVLT33-pigC–pigB. The products were detected spectrophotometrically and quantified relative to the signal upon MAP (**5**) supplementation. Mean values of triplicate measurements with the respective standard deviation are shown. **(B)** Assessment *in vitro* of PigC substrate acceptance, shown as tripyrrole-typical coloration of reaction mixtures after incubation of lysate from PigC-expressing *E. coli* cells with MBC (**6**) and monopyrroles. **(C)** Synthesis scheme of hydroxylated prodiginine **3**. Pyrrole **11a** was supplemented to the MBC producing strain *P. putida* MBC18/pVLT33-pigC–pigB in preparative mutasynthesis to obtain the product **12**. As reference, the compound was also synthesized chemically in a condensation reaction with *Boc*-protected MBC (**6**). By the following semisynthetic hydroboration step, the final hydroxylated prodiginine **3** could be obtained.

Both pyrroles were tested as mutasynthons, but only pyrrole **11a** was converted in *P. putida* MBC18/pVLT33-pigC-pigB (Figure 4A). The hydroxylated group likely prevents the pyrrole **11b** from crossing the cell membrane, since PigC-mediated condensation was observed in experiments with lysate (Figure 4B). Therefore, 150 μM **11a** was applied in a preparative mutasynthesis, yielding 30 mg/L of the corresponding tripyrrole **12** which corresponds to 54% yield.

The hydroxylated prodiginine **3** was synthesized in a final semisynthetic step by hydroboration of the obtained mutasynthesis product **12**, yielding targeted compound **3** in 65% yield (Figure 4C). Notably, for the hydroboration to be successful, the mutasynthesis product had to be purified via reversed phase column chromatography in advance. Overall, a hybrid synthesis route for a hydroxylated prodiginine involving advantageous biosynthesis of the bipyrrole precursor **6** instead of laborious organic synthesis could be established.

### 2.3. Impact of prodiginines on the plant-parasitic nematode *Heterodera schachtii*

**Figure 5:**
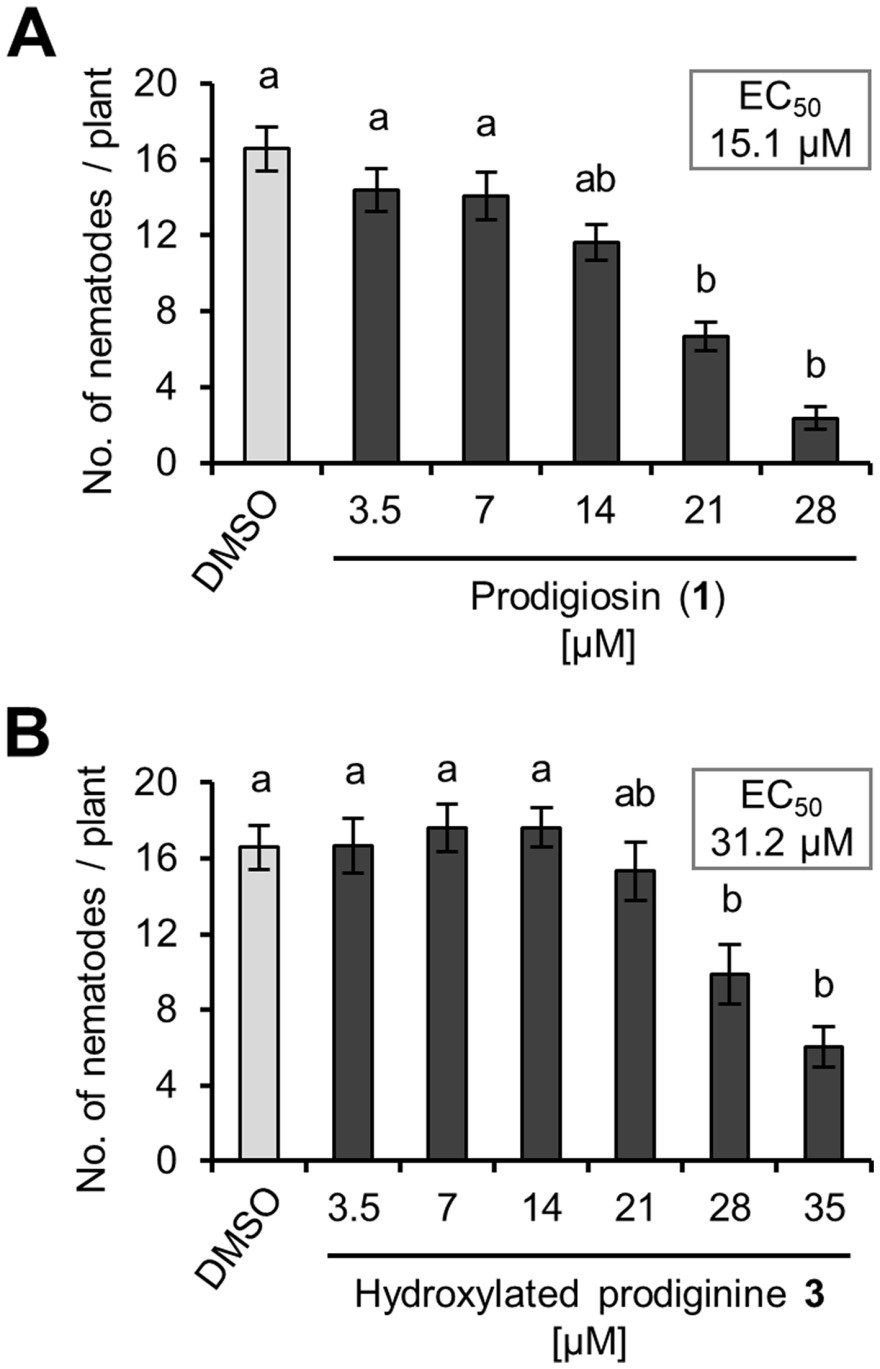
EC_50_ determination of prodiginines on the plant-parasitic nematode *Heterodera schachtii*. **(A)** Impact of prodigiosin (**1**) on nematodal plant infestation. **(B)** Impact of hydroxylated prodiginine **3** on nematodal plant infestation. The impact on *H. schachtii* was assessed as reduction of nematode numbers on *Arabidopsis thaliana*. EC_50_ values (effective concentration that causes a reduction of nematode infestation of *A. thaliana* by 50%) were calculated by using ‘CompuSyn’. Results are expressed as the mean ± standard error of three independent biological replicates (*n* ≥ 18). Different letters indicate statistically significant differences among treatments according to Dunn’s Method (*p* < 0.05).

The main focus in this study was on hydroxylated prodiginine **3** for the above mentioned reasons. Additionally, the natural product prodigiosin (**1**) was used as reference in all subsequent assays. The individual effects of the hydroxylated prodiginine **3** and prodigiosin (**1**) against the PPN *H. schachtii* were determined first. To this end, the half maximal effective concentration (EC_50_) for the reduction of nematode numbers on the model plant *Arabidopsis thaliana* was assessed. Prodiginines were found to inhibit nematode infestation by up to 80%. The EC_50_ (nematode infection) was 31.2 μM for hydroxylated prodiginine **3** and 15.1 μM for prodigiosin (**1**) (Figure 5). Although the synthesis of the previously presented prodiginine **2** is relatively laborious it appeared still interesting to validate its impact on *H. schachtii.* Prodiginine **2** and an alternative prodiginine with a side chain one carbon longer than prodiginine **3** (compound **13**, see Supplementary Information) did not exhibit stronger effects compared to prodiginine **3** (see Supplementary Figure S6) thus justifying our focus on hydroxylated prodiginine **3**.

A next aim was to pinpoint the nematode’s life stage(s) and the time during host-pathogen interaction when the effect of prodiginines on the nematode becomes obvious. Therefore a time-resolved analysis of the nematodes’ reaction to application of prodigiosin (**1**) and the hydroxylated prodiginine **3** was performed. To this end, fitness parameters of second-stage juveniles of *H. schachtii* (J2), nematode infection of *A. thaliana* and nematode development at the plant were investigated. These parameters were evaluated at different time points during exposure of the parasite to the above determined EC_50_ (nematode infection).

**Figure 6:**
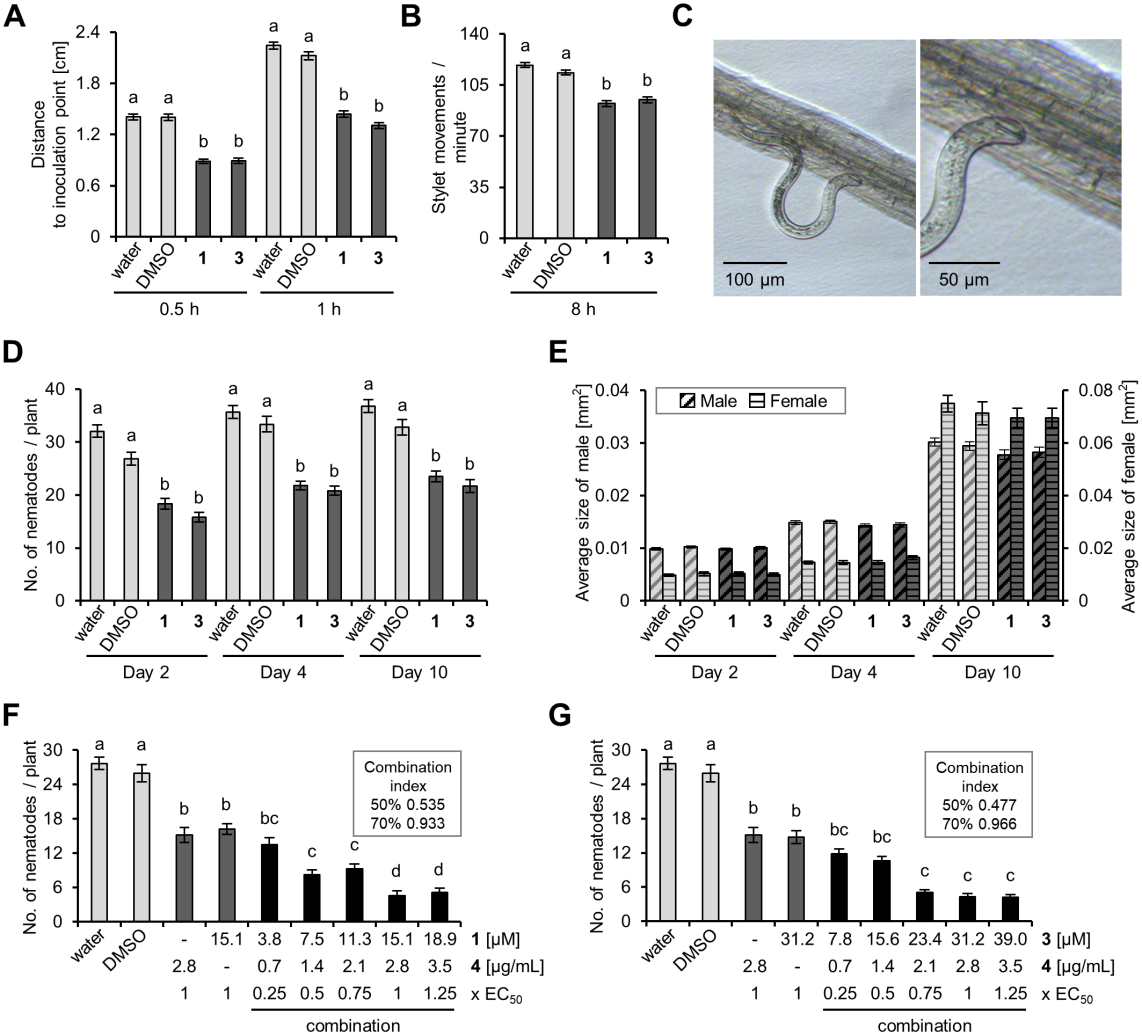
Prodiginine impact on *Heterodera schachtii* fitness parameters, plant parasitism, and combined activity with di-rhamnolipids. **(A)** *H. schachtii* J2 motility, assessed as distance to an inoculation point after incubation. **(B)** Stylet thrusting at *Arabidopsis thaliana* roots, assessed as movements/min. **(C)** *A. thaliana* root with an invading J2. **(D)** Nematode infection rate at *A. thaliana*, investigated at different time points after inoculation. **(E)** Nematode size development, evaluated during 10 days after inoculation. **(F,G)** The combined effect of prodigiosin (**1**) and di-rhamnolipids (**4**) as well as hydroxylated prodiginine **3** and di-rhamnolipids (**4**), respectively, was assessed at 10 days after inoculation. Combination indices were calculated using ‘CompuSyn’. Results are expressed as the mean ± standard error of at least three biological replicates [n ≥ 614 **(A)**; *n* = 40 **(B)**; *n* = 56 (D); *n* ≥ 70 **(E)**; *n* ≥ 20 **(F)**; *n* ≥ 22 **(G)**]. Different letters indicate statistically significant differences among treatments according to Dunn’s Method (*p* < 0.05). EC_50_ is the effective concentration that causes a reduction of nematode infestation of *A. thaliana* by 50%. Prodigiosin (**1**) and hydroxylated prodiginine **3** were used at their EC_50_ concentration in **A**, **B**, **D**.

The motility of *H. schachtii* J2, which were exposed to prodigiosin (**1**) and the hydroxylated prodiginine **3** at their EC_50_ concentrations for 1 h, was significantly reduced by 32 and 39%, respectively, compared to the control (Figure 6A). An investigation of *H. schachtii* J2 stylet movement revealed a significant reduction in the frequency of thrusting at *A. thaliana* roots by 19 and 16% upon prodigiosin (**1**) and hydroxylated prodiginine **3** application, respectively (Figure 6B). This could be the reason that the number of nematodes that successfully penetrated the root epidermis (Figure 6C) and established a sedentary interaction with the plant was significantly reduced by prodiginines by 28 to 41% compared to the control (Figure 6D). Finally, the growth of *H. schachtii* females and males developing from J2 that successfully infected the plant despite prodiginine exposure was slightly reduced but not significantly impaired by prodiginines (Figure 6E).

The presented data provides for the first time specific hints to the mode of action of prodiginines and substantiates that prodiginines exert a direct antagonistic effect on *H. schachtii*. In contrast, the antinematodal effect of rhamnolipids has recently been shown to be indirect, i.e., triggering plant defense mechanisms (Bredenbruch et al., 2023). Based on these findings, it is intriguing to assess the combined effects of prodiginines and rhamnolipids as a synergistic activity against nematodes may be hypothesized. For these investigations the previously evaluated and described mixture of di-rhamnolipid congeners (Bredenbruch et al., 2023) was chosen, which revealed an EC_50_ (nematode infection) of 2.8 μg/mL (corresponding to approximately ~ 4.3 μM; Supplementary Figure S7).

Investigations of the combined activities of prodiginines (**1** and **3**) and di-rhamnolipids (**4**) revealed that the combination of the two compounds was more effective against nematode parasitism than the individual compounds, i.e., the combined application reduced the required concentrations for the same effect (Figures 6F,G). To obtain, for instance, 50% nematode control, it was sufficient to apply ¼ the concentration of hydroxylated prodiginine **3** and di-rhamnolipids (**4**) together or prodigiosin (**1**) and di-rhamnolipids (**4**) together instead of the full dose of each compound individually (see Supplementary Figure S8 for dose response curves and combination index plots).

## 3. Discussion

### 3.1. Development of hybrid synthesis toward tailored hydroxylated prodiginine

The concept of mutasynthesis is highly attractive for synthetic chemists as a method for structural diversification of natural products by using genetically engineered microorganisms (Kirschning et al., 2007). By using nature’s enzymatic biosynthetic machinery for the synthesis of precursor MBC (**6**) this study presents an environmentally friendly way by avoiding heavy metal catalyzed synthesis of *Boc*-**6** (Dairi et al., 2006; Domröse et al., 2015). Furthermore, this approach allows the incorporation of non-native building blocks into new derivatives due to enzyme promiscuity (Sun et al., 2015).

While in our previous studies, prodigiosin (**1**) biosynthesis resulted in titers, e.g., ranging from 60 to 150 mg/L (Domröse et al., 2015, 2017, 2019) depending on the applied conditions, mutasynthetic derivatives were only obtained with 0.6–3.1 mg/L (Klein et al., 2018) or maximally 19.8 mg/L (Klein et al., 2017). In general, this may reflect poor substrate entry into the cells, a low activity of PigC toward unnatural substrates, an insufficient MBC (**6**) supply, or a combination of these factors. In the present study, the MBC (**6**) supply was addressed by implementing expression of MBC (**6**) biosynthetic genes, which successfully led to reaching enhanced titers of 30–34 mg/L. Further improvements are conceivable with alternative ligase enzymes. The new strain setup of *P. putida* MBC18 allows their exchange more easily by use of alternative plasmids, whereas strain *P. putida* pig-r2 Δ*pigD* carried all *pig* genes in the chromosome. In this context, our studies uncovered, that coexpression of *pigB* is essential in this setup. It might be speculated that the enzymes PigN/F and PigB, that naturally deliver the pyrrole precursors, as well as PigC assemble to form a membrane-associated complex (Williamson et al., 2005; Chawrai et al., 2012; Couturier et al., 2019). This may hypothetically ensure fast product release via the membrane. Our findings suggest that an only partial assembly of this putative complex consisting of PigC and PigN/F, but devoid of PigB is not functional. Notably in this context, *Janthinobacterium lividum* appears to contain a *pigCB* fusion in its *pig* gene cluster (Schloss et al., 2010). However, further studies are required to elucidate these interactions.

Previous studies investigated the versatile substrate range of the condensing enzyme PigC (Klein et al., 2017, 2018; Brass et al., 2019). According to these findings, the present study focused on a mutasynthesis approach toward tailored hydroxylated prodiginines by feeding modified pyrroles to the new strain *P. putida* MBC18. In advance, the mutasynthesis experiment was optimized concerning the yields (Figure 2F). Notably, feeding a high amount of MAP (**5**) did not lead to a likewise high amount of prodigiosin (**1**) and thus resulted in relatively low yields up to 17%. Interestingly, by varying the MAP (**5**) concentration in a range of 100–1,000 μm, the prodigiosin (**1**) concentrations obtained (93–167 μm) were relatively constant and did not reflect the magnitude of the fed pyrrole concentration (Figure 2F). This observation could be explained by a substrate inhibition of involved enzymes. A PigC substrate inhibition by MAP (**5**) has been demonstrated in a previous study: conversion assays using different concentrations of MAP (**5**) at a fixed MBC (**6**) concentration suggested an ordered kinetic mechanism in which MBC (**6**) has to bind before MAP (**5**) (Chawrai et al., 2012). In the present study, 62% prodigiosin (**1**), obtained with an adjusted MAP (**5**) concentration, is the highest yield achieved in a preparative scale. However, further investigation and optimization of the mutasynthesis approach may lead to even higher yields, for example by feeding the pyrrole over a period of time to overcome PigC substrate inhibition.

As mentioned above, a strength of mutasynthesis is the substrate promiscuity of the enzymes used. Results of an *in vitro* PigC assay showed acceptance for both pyrroles **11a** and **11b** (Figure 4B). However, using **11b** as mutasynthon in an *in vivo* approach did not result in formation of the targeted hydroxylated prodiginine **3**, rendering an alternative approach necessary. We assume that the hydroxyl group prevents the pyrrole **11b** from crossing the cell membrane. To overcome this issue, pyrrole **11a** was supplemented as mutasynthon to produce prodiginine **12** in a yield of 54%. The hydroxyl group was introduced in a semisynthetic step toward the targeted prodiginine **3**. Notably, the mutasynthesis product **12** had to be purified via reversed-phase column chromatography for the final hydroboration step to succeed. Alternative purification methods, such as aqueous washing steps (sat. NH_4_Cl, sat. NaHCO_3_, sat. Na_2_SO_4_, sat. NaCl or sat. LiCl) or normal phase column chromatography (silica or alox) led to an apparently inactivated prodiginine, which did not show any conversion to the hydroxylated prodiginine **3**, not even under high excess of the hydroboration agent 9-BBN. Quantitative ^1^H-NMR spectroscopy showed, that the purity of the mutasynthesis product, which was isolated by normal phase column chromatography increased from 60 ± 3% to 92 ± 5% after an additional reversed-phase column chromatography. This finding was crucial for the success of the hybrid synthesis toward hydroxylated prodiginine **3**, even if the impurities that led to an inactivation of isolated mutasynthesis products could not be identified and further investigations are necessary.

### 3.2. Prodiginine-induced reduction of the fitness and infectiousness of the plant-parasitic nematode *Heterodera schachtii*

Since PPN mostly inhabit the soil and primarily attack below-surface parts of plants, it is often challenging to control PPN. Microscopic life in the rhizosphere is particularly robust and naturally occurring microbial compounds have been studied and commercialized as potent bioactive ingredients against a diversity of soil-borne pathogens over the past decades (Handelsman and Stabb, 1996; Haas and Défago, 2005; Köhl et al., 2019). Our previous work revealed that prodiginines and rhamnolipids possess activity against the PPN *H. schachtii* (Habash et al., 2020; Bredenbruch et al., 2023). Rhamnolipids exert their activity by triggering plant defense responses effective against nematodes. Unlike that, this current study demonstrated that prodigiosin (**1**) and hydroxylated prodiginine **3** directly antagonize *H. schachtii* J2 by inhibiting motility and stylet thrusting (Figures 6A,B). The J2 is the infective larval stage of *H. schachtii*, which migrates within the soil towards the root. In the natural habitat, *H. schachtii* J2 migrate at the maximum speed when there is no lateral movement and each body part usually follows the movement of its front part (Wallace, 1958). The presence of the investigated prodiginines appears to hinder nematode forward movement.

Stylet thrusting of *H. schachtii* J2 is a prerequisite for destructively invading the host’s root tissue and later—after reaching the vascular cylinder—carefully probing plant cells until a suitable cell is found, which becomes the initial syncytial cell (ISC). The frequency of this stylet movement is significantly reduced by prodiginines, as demonstrated in this study. The continuous vigorous stylet thrusting of J2 is an energy consuming process (Wyss and Zunke, 1986; Grundler et al., 1991; Wyss, 1992; Wyss and Grundler, 1992). Since it has been described that prodiginines can uncouple mitochondrial F-ATPases due to their H^+^/Cl-symport activity (Konno et al., 1998), their direct effect on the cellular energy metabolism might be one explanation for the lower frequency of J2 stylet movements.

Once *H. schachtii* J2 accomplish the formation of permanent feeding sites, they become immobile and change from a migratory into a sedentary form (Wyss, 1992). Prodiginines were observed to interfere with nematode invasion and infection of *A. thaliana*. However, female and male development at the host plant is not influenced by prodiginines at 2 and 4 days after the inoculation, while it is slightly impaired after 10 days (Figures 6D,E). As stated in our previous study, a significant reduction of female size due to prodigiosin application can be observed after 13 days (Habash et al., 2020). Probably, the impact of prodiginines on the pathogen’s development at the root becomes obvious only at later time points after infection. Future studies should investigate this aspect further and additionally determine whether prodiginine exposure interferes with reproduction.

While investigations on the molecular mode of action for the interaction of prodiginines with nematodes are missing, studies of their effects on bacteria document a disturbing impact on biological membranes and reactive oxygen species (ROS) generation (Darshan and Manonmani, 2015, 2016; Suryawanshi et al., 2017; Ravindran et al., 2020; Choi et al., 2021). Whether prodiginines cause disruption of PPN membranes and whether this contributes to the observed effects still needs to be elucidated.

Unlike “single compound - single target” approaches, multicomponent therapeutics enable interactions with multiple targets (Keith et al., 2005; Hopkins, 2007; Yildirim et al., 2007). The discovery of drug–drug combinations offers promising strategies for (1) the improvement of drug treatment efficacy, (2) the reduction of drug dosage to avoid toxicity, and (3) the minimization of drug resistance evolution (Jia et al., 2009; Cheng et al., 2019). Our results demonstrate that, when applying prodiginines and di-rhamnolipids combined, only ¼ of the concentration rather than the full dose of each single agent is required to achieve the same nematode control efficacy (50% reduction of nematode infestation; Figures 6F,G). This validates the two compounds to exert synergistic effects. Intriguingly, by increasing the doses for combinations, the combined effect is diminished and even appears antagonistic. Similar results were reported previously in the analyses of antibacterial effects, which were dependent on the ratio of prodigiosin and rhamnolipids (Hage-Hülsmann et al., 2018).

Synergistic effects between biosurfactants including rhamnolipids and various antibiotics have been described multiple times before (Boonlarppradab et al., 2008; Williamson et al., 2008; Sotirova et al., 2012; Magalhães and Nitschke, 2013; Das et al., 2014; Rossi et al., 2016). On the one hand, rhamnolipids, which exhibit surface-active properties, can increase the solubility of the drug and facilitate its access to target cells (Beal and Betts, 2000; Abdel-Mawgoud et al., 2010). On the other hand, rhamnolipids can perturb the packing of the cell membrane phospholipids by intercalating into the bilayer, which leads to increased permeability of the cell membrane (Dowhan, 1997; Ortiz et al., 2006). As a consequence, the interaction of combined applications facilitates more effective penetration through biological interfaces to better reach the site of action (Bhadoriya et al., 2013; Bnyan et al., 2018; Hage-Hülsmann et al., 2018).

However, the role of rhamnolipids in combined antinematodal activity goes far beyond that. Plants have developed sophisticated defense mechanisms that enhance resistance to their enemies. After perception of rhamnolipids, early events of cell signaling, including calcium influx, MAP kinase activation, ROS accumulation, and defense-related gene stimulation were detected in plants (Garcia-Brugger et al., 2006; Varnier et al., 2009). Besides, rhamnolipids also trigger the activation of a phytohormone-regulated immune signaling network and thus modulate late defense responses to a diversity of phytopathogens (Pieterse et al., 2012; Sanchez et al., 2012). A recent study confirmed that di-rhamnolipids put *A. thaliana* on alert so that the plant responds stronger to *H. schachtii* attack (Bredenbruch et al., 2023). The combined application of rhamnolipids and prodiginines may therefore effectuate molecular structures and processes on multiple levels in both, the plant and the pathogen, and may pose a promising starting point for the development of multicomponent antinematodal agents.

## 4. Materials and methods

### 4.1. Engineering and characterization of *Pseudomonas putida* strains, PigC activity assay

#### 4.1.1. Bacterial strains and standard cultivation conditions

Cultivation of *P. putida* KT2440 (Nelson et al., 2002; Belda et al., 2016) was conducted at 30°C, if not stated otherwise, shaking (130 rpm) in Erlenmeyer shake flasks in LB medium (Carl Roth, Karlsruhe, Germany), or on LB agar plates (LB medium completed with 15 g/L Agar). Small scale production tests were conducted in TB medium (Carl Roth, Karlsruhe, Germany) in Flowerplates (m2p-labs GmbH, Baesweiler, Germany). Cultures of *P. putida* pig-r2 Δ*pigD* (Klein et al., 2017) were supplemented with 80 μg/mL streptomycin, MBC strains created in the present study with 50 μg/mL tetracycline, and MBC strains carrying pVLT33 derived plasmids with 25 μg/mL kanamycin. *Escherichia coli* strains S17-1 (Simon et al., 1983; used for conjugation), and DH5α (Hanahan, 1983; used for cloning) were cultivated at 37°C under constant agitation (120 rpm) in shake flasks in liquid LB medium or on LB agar plates.

#### 4.1.2. Cloning of integrative yTREX vector with MBC biosynthetic genes and ligase expression plasmids

The synthetic MBC biosynthesis-encoding *pig* gene cluster was PCR-amplified in three parts for assembly into the yTREX vector (Domröse et al., 2017; Weihmann et al., 2020) which was linearized with endonuclease I-*Sce*I: *pigA* (primers AD142 + 171, 1,239 bp), *pigFGHI* (primers AD172 + 162, 4,867 bp) and *pigJKLMN* (primers AD163 + 164, 5,033 bp) using vector pPIG (Loeschcke et al., 2013) as template. In addition, *lacZ* was amplified as promotorless gene (primers AD124 + 125, 3,127 bp) using vector pRcExpII2-YF1-FixJ-PFixK2-LacZ (Weihmann et al., 2020) as template. All genes were amplified with the respective 5′-UTR sequences including the ribosome binding sites; primers added homology arms to each of the fragments. Assembly of the vector yTREX-MBC-lacZ, carrying the gene cassette *pigA-pigFGHIJKLMN-lacZ*, in *Saccharomyces cerevisiae* VL6-48 was conducted as previously described (Domröse et al., 2017; Weihmann et al., 2020). Plasmid pVLT33-pigC, carrying the *pigC* gene with an adapted codon usage for *P. putida* (Brands et al., 2020), was used for PigC expression. The *pigB* gene was amplified (primers RW144 + 145, 2090 bp) using yTREX-pig (Domröse et al., 2017) as template. The vector and PCR product were hydrolyzed with endonucleases *Hind*III und *Xba*I and ligated to obtain pVLT33-pigC-pigB. All plasmids and oligonucleotides are listed in Supplementary Table S1.

#### 4.1.3. Generation of MBC producing *Pseudomonas putida* strains

To generate *P. putida* production strains, the plasmid yTREX-MBC-lacZ was transformed into *E. coli* S17-1 and further transferred to *P. putida* KT2440 via conjugation as previously described (Weihmann et al., 2020). Since yTREX constructs do not replicate in *P. putida*, positive selection for strains in which Tn5 transposition of the recombinant yTREX transposon occurred, could be conducted by using LB medium supplemented with tetracycline. In addition, 25 μg/mL irgasan was added to prevent *E. coli* growth. Among exconjugants, production strains were identified visually after 16 h of cultivation on agar plates (Weihmann et al., 2020): The selection medium after conjugation was additionally supplemented with 0.3 mm X-gal (stock solution: 50 mm in DMF), and expression strains were identified by blue color due to β-galactosidase activity.

#### 4.1.4. Analysis of MBC biosynthesis in *Pseudomonas putida*

For verification of MBC biosynthesis in small scale cultivation, expression cultures of *P. putida* pig-r2 Δ*pigD* and MBC strains (denoted with 2, 4, 5, 6, 7, 8, 9, 10, 11, 12, 13, 15, 16, 17, 18, 20, 21 or 22) (n = 4, independent biological replicates) were cultivated in Flowerplates. To this end, 1 mL precultures in LB medium were used for inoculation of 1,1 mL main cultures in TB medium to an OD (650 nm) of 0.05. Flowerplates were covered with breathable air sheets (139.7 μm sterile Rayon films, VWR North America Cat.No. 60941-086) and incubated for 24 h (30°C, 1,400 rpm). After that, 700 μL samples were used for cell harvesting (15,000 rpm, 5 min). The pellet was resuspended in 300 μL methanol and subjected to sonication in a water bath (Sonorex RH 100 H) for 10 min. After centrifugation (15,000 rpm, 5 min), the supernatant was dried at 45°C under reduced pressure (in a Concentrator 5,301). For 2-phase extraction, 300 μL DCM und 300 μL MilliQ-water were added and mixed well, before centrifugation (15,000 rpm, 3 min). The organic lower layer was transferred in a fresh reaction tube and the extraction repeated. The resulting pooled DCM was again dried (45°C, Eppendorf Concentrator 5,301). Samples were resuspended in 200 μL methanol, centrifuged (15,000 rpm, 1 min) and finally subjected to LC–MS analysis or stored until that at 4°C. LC–MS analysis was conducted using an HP 1100 Series LC/MSD (Agilent Analytical Instruments) with an Atlantis T3 column (3 μm, 3*100 mm) from Waters. The eluents water (A) and methanol (B), both supplemented with 0.1% formic acid, were used for gradient chromatography at 0.6 mL/min flow rate: Starting at 90% A and 10% B, increasing to 60% B in 4 min, then in further 2 min to 100% B, which was maintained for 4 min. After that, starting conditions were implemented again (10% B) and held for 1 min. Sample volumes of 10 were injected and detection was accomplished with a G1315A DAD-detector and an G1946A mass spectrometer (API-ES, positive ion mode, single quadrupole detector, m/z-range 100–2000). After verification of specific signals corresponding to the ion [M + H]^+^ of MBC (191 m/z), the UV/Vis signal (364 nm) of the corresponding peaks detected at 6.7 min were evaluated for relative quantification with reference to the values obtained with strain *P. putida* pig-r2 Δ*pigD*. For subsequent routine MBC analysis, HPLC-PDA analysis using an LC-10Ai (Shimadzu Deutschland GmbH, Duisburg, Germany) was also applied with an SPDM10Avp photodiode array detector (PDA), and an Accucore^™^ C18 HPLC column (2.6 μm, 4.6*50 mm) from Thermo Fisher Scientific (Walkham, United States). At a column oven temperature of 30°C, 10 μL samples were analyzed at 1 mL/min flow rate in a gradient elution using water (A) and acetonitrile (B), both with 0.1% formic acid: Starting at 95%A and 5% B for 0.5 min, increasing to 25% B at 0.9 min, to 55% at 9.5 min, and to 98% at 10 min, which was held for 1 min, before returning to 5% B at 11.5 min, which was maintained for 2 min. Chromatograms were recorded at 360 nm and detected MBC at 4.5 min (λ_max_ 363 nm). To analyze MBC in 100 mL cultures, 1 g polyurethane (PU) foam cubes were added as described for mutasynthesis conditions, and extracted with 15 mL ethanol.

#### 4.1.5. Quantification of *pig* gene transcript by RT-qPCR

Reverse transcription (RT) followed by quantitative PCR (qPCR) was employed to quantify mRNA levels of *pigN*. Precultures, grown in LB medium, were used to inoculate 1 mL TB main cultures of *P. putida* pig-r2 Δ*pigD* and MBC18 with pVLT33-pigC-pigB to an OD (650 nm) of 0.05 (*n* = 3, independent biological replicates). These were incubated shaking (1,200 rpm) at 30°C for 4 h, before induction of the latter with 0.5 mm IPTG (10 μL of a 50 mm stock solution in water). After additional 4 h incubation, 500 μL of each culture were harvested to extract total RNA with the NucleoSpin® RNA-Kit (Macherey-Nagel, Düren, Germany). DNAse treatment was conducted in three steps with DNase from Macherey-Nagel, Qiagen (Hilden, Germany) and Ambion (Thermo Fisher Scientific). The Maxima Reverse Transcriptase (Thermo Fisher Scientific) was used for reverse transcription of 2000 ng RNA in 20 μL volumes. The qPCR reaction mix contained 9.2 μL of the cDNA solution (diluted to correspond to 50 ng RNA), 0.4 μL containing 4 pmol of each primer (stocks: 10 pmol/μL), and 10 μL Maxima SYBR Green/ROX qPCR Master Mix (2x; Thermo Fisher Scientific). All qPCR reactions were carried out as technical quadruplicates in addition to the biological triplicates. Controls without reverse transcriptase and without template as well as monitoring of PCR product melting curves ensured signal specificity. Calibration with the pPIG plasmid allowed determination of *pigN* copy numbers in *P. putida* pig-r2 Δ*pigD* and MBC18, which were 177 × 10^4^ (± 4 × 10^4^) and 683 × 10^4^ (± 12 × 10^4^) per 100 ng total RNA, respectively. For direct comparison, the data was evaluated as expression levels relative to the signal of *P. putida* pig-r2 Δ*pigD.* To corroborate comparability, the transcript levels of *rpoD* were analyzed as internal control, which yielded similar results in both strains as expected [571 × 10^4^ (± 16 × 10^4^) and 750 × 10^4^ (± 30 × 10^4^) per 100 ng total RNA].

#### 4.1.6. Mutasynthesis in small scale cultivation

Expression cultures of *P. putida* pig-r2 Δ*pigD* and strains MBC13, −17, −18 with plasmids pVLT33-pigC or pVLT33-pigC-pigB (*n* = 3, independent biological replicates) were cultivated in Flowerplates. Precultures in 1 mL LB medium were used for inoculation of 1 mL main cultures in TB medium to an OD (650 nm) of 0.05. Flowerplates were covered with breathable air sheets and incubated for 4 h (30°C, 1,200 rpm), before 1 mm MAP was supplemented to the cultures (20 μL of a 50 mm stock solution in DMSO) and gene expression in strains with plasmids was induced by addition of 0.5 mm IPTG (10 μL of a 50 mm stock solution in water). Cultivation was subsequently continued for 20 h at 30°C or 25°C. After that, cultures were harvested (12,000 rpm, 2 min). The pellets were pre-resolved with 20 μL milliQ-water and extracted with 500 μL acidified ethanol [4% (*v*/*v*) 1 n HCl in ethanol]. After centrifugation (2 min, 14,000 rpm), 150 μL samples were either diluted by a factor of 10 or directly subjected to spectrophotometric analysis in a microplate reader “Infinite M1000 pro” (Tecan Group LTD., Maennedorf, Switzerland) to measure characteristic absorption spectra from 400 to 700 nm. Plotting the absorption at 535 nm facilitated comparative evaluation. For quantification of prodigiosin formation via the previously published molar extinction coefficient (Domröse et al., 2015), the signal of the Tecan plate reader was calibrated with solutions of known concentration based on ε (535 nm) [m^−1^ cm^−1^] = 139,800 (Domröse et al., 2015). Prodigiosin titers (expressed in mg/L) were determined by considering the molecular weight of the compound (323.432 g/mol) and the extracted culture volume.

#### 4.1.7. PigC substrate acceptance assay

The *in vitro* assay with cell lysate of *E. coli* BL21 (DE3) pET28a(+)-*pigC* was performed as described previously (Klein et al., 2017; Brass et al., 2019). Accordingly, the heterologous expression of PigC was carried out using *E. coli* BL21 (DE3) pET28a(+)-*pigC* cells, which were stored at −20°C after cultivation and harvesting. Frozen cells (1 g) were thawed and resuspended in potassium phosphate buffer (KP_i_ buffer, 50 mm, pH 7.0; 5 mL). The cell suspension was disrupted using a SONOPULS Ultrasonic homogenizer (Bandelin, Berlin, Germany) for 2 × 5 min (five cycles, 40% power), to obtain lysed cells which were used for the PigC substrate acceptance assay. The assay solution contained 440 μL of cell lysate in KP_i_ buffer (50 mm, pH 7.0), 25 μL of a pyrrole **5**, **11a**, **11b** solution in DMSO (20 mm, end concentration: 1 mm), 25 μL of an MBC (**6**) solution in DMSO (20 mm, end concentration: 1 mm) and 10 μL of an ATP∙Na_2_ solution in water (62.5 mm, end concentration: 1.25 mm). The reaction mixture was shaken in a 1.5 mL tube at 300 rpm and 30°C for 4 h. The supernatant was removed after centrifugation (5 min, 21,100 rcf, 23°C) and the prodiginine pellet was resuspended in 300 μL acidic ethanol [4% (*v*/*v*) 1 n HCl in ethanol]. After centrifugation (5 min, 21,100 rcf, 23°C), the supernatant was transferred in a new 1.5 mL reaction tube and documented photographically.

### 4.2. Chemical precursor syntheses

#### 4.2.1. General experimental procedures for chemical syntheses

All reactions were carried out under nitrogen atmosphere and magnetic stirring. Used glassware and magnetic stirring bars were dried previously at 110°C. All starting materials were purchased from commercial sources without further purification unless stated otherwise. Dichloromethane, diethyl ether, ethyl acetate (EtOAc) and petroleum ether (PE) were distilled prior to use. Tetrahydrofuran (THF) was used directly. Reactions were monitored by GC–MS, ^1^H-NMR and thin layer chromatography (TLC; Polygram SIL G/UV254, Macherey-Nagel) using an acidic solution of *p*-anisaldehyde for staining or UV light at 245 nm for visualization. Purification of reaction products was carried out by flash chromatography on silica gel 60 (particle size 0.040–0.063 mm, 230–240 mesh, Macherey-Nagel). Analytics were carried out as described in the Supplementary Information, including Supplementary Tables S2, S3, and Supplementary Figure S5. The precursors 2-methyl-3-*N*-amylpyrrole (MAP, **5**), *tert*-butyloxycarbonyl-5′-formyl-4′-methoxy-1*H*,1′*H*-2,2′-bipyrrole (*boc*-MBC, *Boc*-**6**) and prodigiosin (**1**) as chemical references were synthesized as previously described (Dairi et al., 2006; Domröse et al., 2015).

#### 4.2.2. *N*-methoxy-*N*-methyloct-7-enamide (**8**)

To a solution of 7-octenoic acid (2 mL, 13.0 mmol, 1.0 eq.) in dichloromethane (70 mL) was added *N*,O-dimethylhydroxylamine hydrochloride (1.19 g, 19.5 mmol, 1.50 eq.), *N*-(3-dimethylaminopropyl)-*N*′ethylcarbodiimid hydrochloride (3.03 g, 19.5 mmol, 1.5 eq.) and 4-(dimethylamino)pyridine (2.38 g, 19.5 mmol, 1.5 eq.). After stirring for 22 h at 22°C, the reaction mixture was quenched with a saturated solution of NaCl and extracted with dichloromethane (3 × 50 mL). The combined organic layers were first washed with 1 N HCl, afterwards with saturated NaHCO_3_ solution and dried over MgSO_4_. After removal of the solvent under reduced pressure *N*-methoxy-*N*-methyloct-7-enamide (**8**, 2.29 g, 12.4 mmol, 95%) was obtained as a yellow oil and was used for the following experiment without further purification. *R*_f_ = 0.15 (PE/EtOAc 85:15); ^**1**^**H-NMR** (600 MHz, CDCl_3_): *δ* [ppm] = 1.36 (m_c_, 2H, 4-H), 1.41 (m_c_, 2H, 5-H), 1.64 (m_c_, 2H, 3-H), 2.05 (m_c_, 2H, 6-H), 2.41 (t, ^3^*J*_2,3_ = 7.7 Hz, 2H, 2-H), 3.18 (s, 3H, 1′′-H), 3.68 (s, 3H, 1′-H), 4.93 (dd, ^cis,3^*J*_8a,7_ = 10.2 Hz, ^2^*J*_8a,8b_ = 1.2 Hz, 1H, 8-H_a_), 4.99 (ddd, ^trans,3^*J*_8b,7_ = 17.1 Hz, ^2^*J*_8b,8a_ = 1.8 Hz, ^4^*J*_8b,6_ = 1.8 Hz, 1H, 8-H_b_), 5.80 (ddt, ^trans,3^*J*_7,8b_ = 17.0 Hz, ^cis,3^*J*_7,8a_ = 10.2 Hz, ^3^*J*_7,6_ = 6.7 Hz, 1H, 7-H); ^**13**^**C-NMR** (151 MHz, CDCl_3_): *δ* [ppm] = 24.6 (C-3), 28.8 (C-4), 29.1 (C-5), 32.0 (C-2), 32.3 (C-1′′) 33.8 (C-6) 61.3 (C-1′), 114.5 (C-8), 139.1 (C-7); **IR** (ATR-film): $\tilde{v}$ [1/cm] = 3,074, 2,931, 2,855, 1,667, 1,463, 1,415, 1,384, 1,178, 998, 910, 732; **MS** (APCI, positive ion): *m*/*z* = 186 [(M)^+^], 97, 83, 55.

#### 4.2.3. Non-8-en-2-one (**9**)

*N*-methoxy-*N*-methyloct-7-enamide (**8**, 2.29 g, 12.4 mmol, 1.0 eq.) was dissolved in dry THF (100 mL) and methylmagnesium chloride (3 M in diethyl ether, 12.4 mL, 37.1 mmol, 3.0 eq.) was added within 15 min at 0°C. The reaction mixture was stirred for 1.5 h at 0°C and afterwards quenched by the addition of a saturated solution of NH_4_Cl at 0°C and extracted with dichloromethane (3 × 60 mL). The combined organic layers were dried over MgSO_4_ and the solvent was evaporated under reduced pressure to yield non-8-en-2-one (**9**, 1.63 g, 11.6 mmol, 94%) as a yellowish oil without further purification. *R*_f_ = 0.40 (PE/EtOAc 85:15); ^**1**^**H-NMR** (600 MHz, CDCl_3_): *δ* [ppm] = 1.25–1.34 (m, 2H, 5-H), 1.35–1.43 (m, 2H, 6-H), 1.58 (tt, ^3^*J*_4,3_ = 7.5 Hz, ^3^*J*_4,5_ = 7.5 Hz, 2H, 4-H), 2.05 (m, 2H, 7-H), 2.13 (s, 3H, 1-H), 2.42 (t, ^3^*J*_3,4_ = 7.5 Hz, 2H, 3-H), 4.93 (ddt, ^cis,3^*J*_9a,8_ = 10.2 Hz, ^4^*J*_9a,7_ = 2.3 Hz, ^2^*J*_9a,9b_ = 1.2 Hz, 1H, 9-H_a_), 4.99 (dd, ^trans,3^*J*_9b,8_ = 17.1 Hz, ^2^*J*_9b,9a_ = 1.7 Hz, 1H, 9-H_b_), 5.79 (ddt, ^trans,3^*J*_8,9b_ = 17.0 Hz, ^cis,3^*J*_8,9a_ = 10.2 Hz, ^3^*J*_8,7_ = 6.7 Hz, 1H, 8-H); ^**13**^**C-NMR** (151 MHz, CDCl_3_): *δ* [ppm] = 23.8 (C-4), 28.7 (C-5), 28.8 (C-6), 30.0 (C-1), 33.7 (C-7), 43.9 (C-3), 114.6 (C-9), 139.0 (C-8), 209.4 (C-2); **IR** (ATR-film): $\tilde{v}$ [1/cm] = 2,928, 2,855, 1738, 1721, 1,443, 1,363, 1,217, 907; **MS** (APCI, positive ion): *m*/*z* = 123.

#### 4.2.4. Dec-9-en-2-one oxime (**10**)

To a solution of the non-8-en-2-one (**9**, 1.73 g, 12.4 mmol, 1.0 eq.) in ethanol (6.2 mL) was added pyridine (0.78 g, 0.8 mL, 9.89 mmol, 0.8 eq.) and grounded hydroxylamine hydrochloride (1.5 eq.). The reaction mixture was refluxed for 2 h and afterwards extracted with dichloromethane (3 × 25 mL). The combined organic layers were washed with 1 N HCl (3 × 20 mL) and dried over MgSO_4_. After removal of the solvent under reduced pressure, dec-9-en-2-one oxime (**10**, 1.76 g, 11.3 mmol, 92%) was obtained in a diastereomeric mixture of *E*:*Z* (1:3) without further purification. *R*_f_ = 0.34 (PE/EtOAc 80:20); ^**1**^**H-NMR** (600 MHz, CDCl_3_): *δ* [ppm] = 1.28–1.36 (m, 2H, 5-H), 1.37–1.44 (m, 2H, 6-H), 1.51 (tt, ^3^*J*_4,3_ = 7.5 Hz, ^3^*J*_4,5_ = 7.5 Hz, 2H, 4-H), 1.87 (s, 3H, 1-H), 2.05 (dt, ^3^*J*_7,6_ = 6.4 Hz, ^3^*J*_7,8_ = 6.4 Hz, 2H, 7-H), 2.18 (t, ^3^*J*_3,4_ = 7.5 Hz, 2H, 3-H), 4.94 (ddt, ^cis,3^*J*_9a,8_ = 10.2 Hz, ^4^*J*_9a,7_ = 2.4 Hz, ^2^*J*_9a,9b_ = 1.3 Hz, 1H, 9-H_a_), 5.00 (dd, ^trans,3^*J*_9b,8_ = 17.1 Hz, ^2^*J*_9b,9a_ = 1.5 Hz, 1H, 9-H_b_), 5.80 (ddt, ^trans,3^*J*_8,9b_ = 17.0 Hz, ^cis,3^*J*_8,9a_ = 10.4 Hz, ^3^*J*_8,7_ = 6.6 Hz, 1H, 8-H); ^**13**^**C-NMR** (151 MHz, CDCl_3_): *δ* [ppm] = 13.4 (C-1), 26.2 (C-4), 28.7 (C-5), 28.8 (C-6), 33.7 (C-7), 35.8 (C-3), 114.4 (C-9), 139.1 (C-8), 159.1 (C-2); **IR** (ATR-film): $\tilde{v}$ [1/cm] = 3,074, 2,929, 2,855, 1,637, 1,461, 1,369, 910; **MS** (APCI, positive ion): *m*/*z* = 156 [(M)^+^], 83; **HRMS** (ESI, positive ion): calculated for C_9_H_18_NO [(M + H)]^+^ = 156.1383, found = 156.1384.

#### 4.2.5. 3-(hex-5-en-1-yl)-2-methyl-1*H*-pyrrole (**11a**)

A reaction mixture of dec-9-en-2-one oxime (**10**, 0.72 g, 4.64 mmol, 1.0 eq.), potassium hydroxide (1.30 g, 46.4 mmol, 5.0 eq.), water (62.7 mg, 62.3 μL, 3.48 mmol, 0.75 eq.) and DMSO (8.9 mL) was heated at 90–100°C (using previously degassed DMSO on molecular sieves (3.5 Å) could increase the yield). Over a period of 2 h, a solution of 1,2-dichloroethane (1.38 g, 1.10 mL, 13.9 mmol, 3.5 eq.) in DMSO (1 mL) was added dropwise. After 1 h of 1,2-dichloroethane addition a secondary amount of potassium hydroxide (5.0 eq.) was added. After an overall reaction time of 4 h at 90–100°C the reaction mixture was allowed to reach room temperature and subsequently ice water (20 mL) was added. The mixture was extracted with diethyl ether (3 × 20 mL) and the combined organic layers were dried over MgSO_4_. After the solvent was evaporated under reduced pressure the crude product was purified by column chromatography on silica gel [PE:dichloromethane (60:40) + 1% trimethylamine (*v*/*v*)] to obtain 3-(hex-5-en-1-yl)-2-methyl-1*H*-pyrrole (**11a**, 228 mg, 1.40 mmol, 30%) as yellowish oil. *R*_f_ = 0.37 (PE/dichloromethane 60:40); ^**1**^**H-NMR** (600 MHz, CDCl_3_): *δ* [ppm] = 1.44 (tt, ^3^*J*_3′′,2′′_ = 7.5 Hz, ^3^*J*_3′′,4′′_ = 7.5 Hz, 2H, 3′′-H), 1.51–1.59 (m, 2H, 2′′-H), 2.02–2.14 (m, 2H, 4′′-H), 2.18 (s, 3H, 1′-H), 2.39 (t, ^3^*J*_1′′,2′′_ = 7.6 Hz, 2H, 1′′-H), 4.93 (dd, ^cis,3^*J*_6a′′,5′′_ = 10.2 Hz, ^2^*J*_6a′′,6b′′_ = 1.2 Hz, 1H, 6′′-H_a_), 5.00 (dd, ^trans,3^*J*_6b′′,5′′_ = 17.1 Hz, ^2^*J*_6b′′,6a′′_ = 1.7 Hz, 1H, 6′′-H_b_), 5.82 (ddt, ^trans,3^*J*_5′′,6b′′_ = 16.9 Hz, ^cis,3^*J*_5′′,6a′′_ = 10.2 Hz, ^3^*J*_5′′,4′′_ = 6.7 Hz, 1H, 5′′-H), 6.01 (dd, ^4^*J*_4,1_ = 2.8 Hz, ^3^*J*_4,5_ = 2.8 Hz, 1H, 4-H), 6.59 (dd, ^3^*J*_5,1_ = 2.7 Hz, ^3^*J*_5,4_ = 2.7 Hz, 1H, 5-H), 7.70 (brs, 1H, 1-NH); ^**13**^**C-NMR** (151 MHz, CDCl_3_): *δ* [ppm] = 11.2 (C-1′), 25.9 (C-1′′), 28.9 (C-3′′), 31.0 (C-2′′), 33.9 (C-4′′), 109.0 (C-4), 114.3 (C-6′′), 115.0 (C-5), 119.7 (C-3), 123.4 (C-2), 139.4 (C-5′′); **IR** (ATR-film): $\tilde{v}$ [1/cm] = 3,385, 2,928, 2,855, 1741, 1,467, 1,363, 1,217, 907, 712; **MS** (APCI, positive ion): *m*/*z* = 164 [(M)^+^]; 121, 108; **HRMS** (ESI, positive ion): calculated for C_11_H_18_N [(M + H)]^+^ = 164.1434, found = 164.1433.

#### 4.2.6. 6-(2-methyl-1*H*-pyrrol-3-yl)hexan-1-ol (**11b**)

9-BBN (0.5 n in THF, 5.54 g, 3.10 mmol, 2.2 eq.) was added to a solution of 3-(hex-5-en-1-yl)-2-methyl-1*H*-pyrrole (**11a**, 230 mg, 1.41 mmol, 1.0 eq.) in dry THF (11.6 mL) over a period of 15 min at 0°C. After stirring for 1 h at 0°C, the reaction mixture was heated up at 70°C under reflux for 3 h. Subsequently an aqueous solution of 3 n NaOH (2.35 g, 2.35 mL, 7.05 mmol, 5.0 eq.) and 30% H_2_O_2_ (2.24 g, 2.00 mL, 19.7 mmol, 14.0 eq.) was added at 0°C. After 1 h at 0°C the reaction mixture was allowed to reach room temperature and was stirred for further 15 h at 22°C. Ice water (40 mL) was added and the mixture was extracted with dichloromethane (3 × 50 mL). The combined organic layer was dried over MgSO_4_, the solvent was evaporated under reduced pressure and the crude product was purified by column chromatography on silica gel [PE:ethyl acetate (70:30 to 50:50) + 1% trimethylamine (*v*/*v*)] to isolate 6-(2-methyl-1*H*-pyrrol-3-yl)hexan-1-ol (**11b**, 193 mg, 1.06 mmol, 76%) as orange oil. *R*_f_ = 0.20 (PE/EtOAc 70:30); ^**1**^**H-NMR** (600 MHz, CDCl_3_): *δ* [ppm] = 1.20 (brs, 1H, 1-OH), 1.34–1.42 (m, 4H, 3-H, 4-H), 1.51–1.61 (m, 4H, 2-H, 5-H), 2.18 (s, 3H, 1′′-H), 2.39 (t, ^3^*J*_6,5_ = 7.7 Hz, 2H, 6-H), 3.64 (t, ^3^*J*_1,2_ = 7.1 Hz, 2H, 1-H), 6.00 (dd, ^4^*J*_4′,1′_ = 2.8 Hz, ^3^*J*_4′,5′_ = 2.8 Hz, 1H, 4′-H), 6.59 (dd, ^3^*J*_5′,1′_ = 2.7 Hz, ^3^*J*_5′,4′_ = 2.7 Hz, 1H, 5′-H), 7.72 (brs, 1H, 1′-NH); ^**13**^**C-NMR** (151 MHz, CDCl_3_): *δ* [ppm] = 11.2 (C-1′′), 25.8 (C-4), 26.0 (C-6), 29.4 (C-3), 31.4 (C-5), 33.0 (C-2), 62.7 (C-1), 109.0 (C-4′), 115.0 (C-5′), 119.7 (C-3′), 123.4 (C-2′); **IR** (ATR-film): $\tilde{v}$ [1/cm] = 3,373, 2,928, 2,855, 1735, 1,436, 1,363, 1,223, 1,059, 748; **MS** (APCI, positive ion): *m*/*z* = 182 [(M)^+^], 164, 108; **HRMS** (ESI, positive ion): calculated for C_11_H_20_NO [(M + H)]^+^ = 182.1539, found = 182.1539.

### 4.3. Muta- and semisynthetic hydroxylated prodiginine production

#### 4.3.1. General procedure for preparative scale mutasynthesis

A preculture of *P. putida* MBC18 with pVLT33-pigC-pigB in LB medium (25 μg/mL kanamycin) was incubated in a shake flask at 30°C and 130 rpm overnight. Five main cultures of 100 mL each in TB medium (25 μg/mL kanamycin) were inoculated to an OD_650_ of 0.05 and incubated in 1 l baffled flask with air-sheet seals for 4 h at 30°C and 130 rpm. The pyrrole precursor was dissolved in DMSO (5–50 mm stock, 10 mL). To each culture 2 mL of pyrrole stock solution was added (final concentration 0.1–1.0 mm). Induction was performed with 0.5 mm IPTG (50 mm stock in dH2O; 1 mL). After an additional hour at 30°C and 130 rpm, 1 g of polyurethane (PU) foam cubes (Softpur, Göllheim, Germany: Softpur foam, 25 kgm^−3^ density, 4 kPa compression hardness, each cube approximately 1 cm^3^) were added to each culture and the cultures were incubated for further 23 h at 30°C and 130 rpm. After a total of 28 h of cultivation, the foam cubes were wrung out, washed with dH_2_O, and then extracted with diethyl ether (250–500 mL) in a soxhlet extractor. After evaporation of the solvent, the crude product was dissolved in diethyl ether (20 mL), washed with water, and the aqueous layer was extracted with dichloromethane (3 × 15 mL). The combined organic layers were washed with saturated NaCl (20 mL) and dried over MgSO_4_. After evaporation of the solvent, the crude product was purified by flash column chromatography on silica gel (CH_2_Cl_2_ + 1.0–1.5% NH_3_ in MeOH). For subsequent hydroboration, the product was further purified by reversed-phase chromatography (column: ISAspher 100–5 C18 AQ, 5 μm, 150*20 mm from ISERA GmbH, Düren, Germany; column oven: 35°C; eluent: 60:40 acetonitrile:water + 0.1% formiate; flow rate 15 mL/min; injection of samples in 1 mL ethanol) to obtain the mutasynthesis product as red solid.

#### 4.3.2. 4-Methoxy-5-[(5-methyl-4-hex-5-en-1-yl-2*H*-pyrrol-2-yliden)methyl]-1*H*,1′*H*-2,2′-bipyrrol (**3**)

According to the general procedure for preparative mutasynthesis and the use of pyrrole **11a** (12.2 mg, 75.0 μmol, 7.5 mm in DMSO, end concentration in 500 mL culture: 0.15 mm) as precursor prodiginine **12** (15.0 mg, 40.3 μmol, 54%) was obtained as red solid. *R*_f_ = 0.15 (dichloromethane); ^**1**^**H-NMR** (600 MHz, CDCl_3_): *δ* [ppm] = 1.43 (tt, ^3^*J*_8′′,7′′_ = 7.5 Hz, ^3^*J*_8′′,9′′_ = 7.5 Hz, 2H, 8′′-H), 1.53–1.59 (m, 2H, 7′′-H), 2.08 (dt, ^3^*J*_9′′,8′′_ = 7.2 Hz, ^3^*J*_9′′,10′′_ = 7.2 Hz, 2H, 9′′-H), 2.41 (t, ^3^*J*_6′′,7′′_ = 7.6 Hz, 2H, 6′′-H), 2.54 (s, 3H, 12′′-H), 4.01 (s, 3H, 7-H), 4.95 (dd, ^cis,3^*J*_11a′′,10′′_ = 10.2 Hz, ^2^*J*_11a′′,11b′′_ = 2.2 Hz, 1H, 11′′-H_a_), 5.01 (dd, ^trans,3^*J*_11b′′,10′′_ = 17.1 Hz, ^2^*J*_11b′′,11a′′_ = 1.7 Hz, 1H, 11′′-H_b_), 5.80 (ddt, ^trans,3^*J*_10′′,11b′′_ = 16.9 Hz, ^cis,3^*J*_10′′,11a′′_ = 10.2 Hz, ^3^*J*_10′′,9′′_ = 6.7 Hz, 1H, 10′′-H), 6.08 (d, ^4^*J*_3,1_ = 2.0 Hz, 1H, 3-H), 6.36 (dd, ^3^*J*_4′,5′_ = 3.7 Hz, ^4^*J*_4′,1′_ = 2.3 Hz, 4′-H), 6.68 (d, ^3^*J* = 2.6 Hz), 6.92 (ddd, ^3^*J* = 3.9 Hz, ^3^*J* = 2.5 Hz, ^3^*J* = 1.3 Hz), 6.96 (s, 1H), 7.24 (d, ^4^*J*_3′′,1′′_ = 2.7 Hz, 1H, 3′′-H), 12.58 (brs, 1H, 1′-NH), 12.75 (brs, 2H, 1-NH, 1′′-NH); ^**13**^**C-NMR** (151 MHz, CDCl_3_): *δ* [ppm] = 12.6 (C-12′′), 25.3 (C-6′′), 28.6 (C-8′′), 29.7 (C-7′′), 33.7 (C-9′′), 58.9 (C-7), 93.0 (C-3), 111.9 (C-4′), 114.7 (C-10′′), 116.1 (C-8), 117.2 (C-3′), 120.9 (C-5), 122.4 (C-2′), 125.3 (C-2′′), 127.1 (C-5′), 128.3 (C-4′′), 128.4 (C-3′′), 138.8 (C-9′′), 146.9 (C-5′′), 147.9 (C-2), 165.9 (C-4); **IR** (ATR-Film): $\tilde{v}$ [1/cm] = 3,163, 3,099, 2,974, 2,928, 2,857, 1,630, 1,604, 1,544, 1,512, 1,414, 1,356, 1,261, 1,158, 1,137, 1,044, 993, 960, 838, 756; **MS** (APCI, positive-Ion): *m*/*z* = 336 [(M)^+^], 266, 163; **HRMS** (ESI, positive ion): calculated for C_21_H_26_N_3_O [(M + H)]^+^ = 336.2070, found = 336.2076.

#### 4.3.3. Semisynthesis toward 6-(2-((4-methoxy-1*H*,1′*H*-(2,2′-bipyrrole)-5-yl) methylene)- 5-methyl-2*H*-pyrrole-4-yl)hexan-1-ol (**3**)

9-BBN (0.5 n in THF, 94.2 mg, 0.05 mmol, 2.2 eq.) was added to a solution of 4-Methoxy-5-((5-methyl-4-hex-5-en-1-yl-2H-pyrrole-2-yliden)methyl)-1*H*,1′*H*-2,2′-bipyrrole (**12**, 9 mg, 0.02 mmol, 1.0 eq.) in dry THF (2 mL) over a period of 15 min at 0°C. After stirring for 1 h at 0°C, the reaction mixture was heated up at 70°C under reflux for 3 h. Subsequently an aqueous solution of 3 N NaOH (39.9 mg, 105 μL, 0.12 mmol, 5.0 eq.) and 30% H_2_O_2_ (38.0 mg, 34.1 μL, 0.34 mmol, 14.0 eq.) was added at 0°C. After 1 h at 0°C the reaction mixture was allowed to reach room temperature and was stirred for further 15 h at 22°C. Ice water (10 mL) was added and the mixture was extracted with dichloromethane (3 × 15 mL). The combined organic layer was dried over MgSO_4_, the solvent was evaporated under reduced pressure and the crude product was purified by column chromatography on silica gel [dichloromethane + 0.5–4.0% trimethylamine (*v*/*v*)] to isolate 6-(2-((4-methoxy-1*H*,1′*H*-(2,2′-bipyrrole)-5-yl)methylene)-5-methyl-2*H*-pyrrole-4-yl)hexan-1-ol (**3**, 6.10 mg, 0.02 mmol, 65%) as red solid. *R*_f_ = 0.16 (PE/EtOAc 50:50); ^**1**^**H-NMR** (600 MHz, CDCl_3_): *δ* [ppm] = 1.28 (brs, 1H, 12′′-OH), 1.37 (m_c_, 4H, 8′′-H, 9′′-H), 1.50–1.63 (m, 4H, 7′′-H, 10′′-H), 2.40 (t, ^3^*J*_6′′,7′′_ = 7.6 Hz, 2H, 6′′-H), 2.54 (s, 3 H, 13′′-H), 3.64 (t, ^3^*J*_11′′,10′′_ = 6.6 Hz, 2H, 11′′-H), 4.00 (s, 3H, 7-H), 6.08 (d, ^4^*J*_3,1_ = 1.9 Hz, 1H, 3-H), 6.35 (dd, ^3^*J*_4′,5′_ = 4.3 Hz, ^4^*J*_4′,1′_ = 2.0 Hz, 1H, 4′-H), 6.67 (d, ^4^*J*_3′′,1′′_ = 2.6 Hz, 1H, 3′′-H), 6.92 (ddd, ^3^*J*_3′,4′_ = 3.9 Hz, ^4^*J*_3′,5′_ = 2.5 Hz, ^4^*J*_3′,1′_ = 1.4 Hz, 1H, 3′-H), 6.94 (s, 1H, 8-H), 7.23 (dd, ^3^*J*_5′,4′_ = 2.7 Hz, ^3^*J*_5′,1′_ = 1.3 Hz, 1-H, 5′-H), 12.56 (brs, 1H, 1′-NH), 12.72 (brs, 2H, 1-NH, 1′′-NH); ^**13**^**C-NMR** (151 MHz, CDCl_3_): *δ* [ppm] = 12.6 (C-13′′), 25.0 (C-6′′), 25.7 (C-8′′), 29.1 (C-9′′), 30.2 (C-7′′), 32.9 (C-10′′), 58.9 (C-7), 63.1 (C-11′′), 93.0 (C-3), 111.9 (C-4′), 116.3 (C-8), 117.3 (C-3′), 120.9 (C-5), 122.4 (C-2′), 125.3 (C-2′′), 127.2 (C-5′), 128.3 (C-4′′), 128.4 (C-3′′), 147.0 (C-5′′), 148.0 (C-2), 166.0 (C-4); **IR** (ATR-film): $\tilde{v}$ [1/cm] = 3,422, 3,172, 2,930, 2,861, 1,630, 1,602, 1,543, 1,511, 1,363, 1,261, 1,137, 960, 748; **MS** (APCI, positive ion): *m*/*z* = 354 [(M)^+^], 279, 157; **HRMS** (ESI, positive ion): calculated for C_21_H_28_N_3_O_2_ [(M + H)]^+^ = 354.2176, found = 354.2179.

### 4.4. Assessment of effects on the plant-parasitic nematode *Heterodera schachtii*

#### 4.4.1. Plant material and nematode culture

*Arabidopsis thaliana* Columbia (Col-0) seeds were surface-sterilized by soaking in 0.7% sodium hypochlorite for 5 min and submerging them in 70% (*v*/*v*) ethanol for 1 min. Subsequently, the seeds were rinsed with sterile distilled water 5 times, dried at room temperature for 4 h and stored at 4°C for further experiments. *H. schachtii* cysts were harvested from the roots of mustard (*Sinapsis alba*), which was grown aseptically on modified Knop agar medium, and submerged with sterile 3 mm ZnCl_2_ in the Baermann funnel (Grundler et al., 1991). After 7 days, the freshly hatched second-stage juveniles (J2s) were collected for subsequent analysis. All preparation procedures were performed under aseptic conditions.

#### 4.4.2. EC_50_ (nematode infection) determination of di-rhamnolipids and prodiginines

EC_50_ determination of selected compounds was performed in an *in vitro* agar system: Petri dishes (90 mm diameter) were filled with modified Knop medium (Sijmons et al., 1991; Matera et al., 2021) supplemented with prodiginines or di-rhamnolipids at different concentrations (Supplementary Table S4). Stock solutions of prodiginines in DMSO were applied to implement a final concentration of 0.5% DMSO. The di-rhamnolipids, which were obtained by microbial production as a congener mixture as previously described (Bredenbruch et al., 2023), were solved in water. Accordingly, modified Knop medium alone or supplemented with 0.5% (v/v) DMSO served as control. On the medium, 2 surface-sterilized *A. thaliana* seeds were germinated aseptically and incubated in a climate chamber under a red/blue light with a 16-h/8-h light/dark photoperiod at 24°C (Sijmons et al., 1991). At 12 days post seeding, each plant was inoculated with approximately 60 *H. schachtii* J2s. At 10 days post inoculation, the total number of males and females on each plant was counted under a Stereo Microscope (Leica, Germany). EC_50_ was determined by using software ‘CompuSyn’ (Chou and Martin, 2005). Three independent biological replicates of the experiment were performed. Each biological replicate included at least 6 technical replicates (plants) per variant.

#### 4.4.3. Determination of the combinatorial effect of compounds on nematode infection

Assays to measure combinatorial effects were carried out in an analogous experimental set-up as the EC_50_ determination described above. The concentration of compounds alone or in combination is detailed in Supplementary Table S5. The compound combination effects (antagonistic, additive or synergistic) were evaluated according to the Combination Index Plot obtained by the software ‘CompuSyn’ (Chou and Martin, 2005). The experiments were performed independently in triplicate.

#### 4.4.4. Time-resolved analyses of the compounds’ impact on nematodes

The time-resolved analysis consisted of four assays investigating nematode motility, stylet thrusting, infection, and development. Petri dishes (90 mm diameter) were filled with modified Knop medium supplemented with prodigiosin (**1**) or hydroxylated prodiginine **3** at the determined EC_50_, which is 15.1 and 31.2 μM, respectively. Modified Knop medium alone or supplemented with 0.5% (*v*/*v*) DMSO served as controls.

##### 4.4.4.1. Nematode motility

Approximately 30 *H. schachtii* J2 were inoculated in the center of a Petri dish containing the test compound. The location of J2s was documented after 30 and 60 min and the distance to the inoculation point was measured by Image J (Schneider et al., 2012). The experiment was performed independently in quadruplicate.

##### 4.4.4.2. Nematode stylet thrusting

Two surface-sterilized *A. thaliana* seeds were germinated aseptically on the medium containing the test compound. At 12 days post seeding, approximately 60 *H. schachtii* J2 were inoculated to each plant. At 6 h post inoculation, 10 J2, which successfully penetrated the root epidermis, were tracked under a Stereo Microscope (Leica, Germany) in order to count the number of stylet movements for 5 min. The experiment was performed independently in quadruplicate.

##### 4.4.4.3. Nematode infection and development

Two surface-sterilized *A. thaliana* seeds were germinated aseptically on the medium containing the test compound. At 12 days post seeding, approximately 60 *H. schachtii* J2 were inoculated to each plant. At 2, 4 and 10 days post inoculation, the number of males and females was counted, and the size of male and female nematodes was measured under a LeicaS4E Stereo Microscope (Leica, Germany) equipped with Leica Application Suite (LAS) software. Four independent biological replicates with 14 plants per variant and biological replicate (*n* = 56) were conducted for the nematode infection assay. Three independent biological replicates with in total *n* = 70 technical replicates (nematodes) were performed for the development experiment.

#### 4.4.5. Statistical analysis of bioactivity evaluating data

All data are expressed as mean ± standard error (SE). Statistical analysis was performed by using one-way analysis of variance (ANOVA; *p* < 0.05; SIGMAPLOT 12.5, Systat Software, Inc., San Jose, CA, United States).

## Data availability statement

The original contributions presented in the study are included in the article/Supplementary material, further inquiries can be directed to the corresponding authors.

## Author contributions

AL, AS, JP, FG, K-EJ, and TD conceived the research concept and designed the experiments. RW, FG, SI, NB, KB, DK, TW, and HB performed microbiological work as well as chemical syntheses and production of prodiginines. CM, TT, and LB provided rhamnolipids. MH, XX, and LR conducted investigations of antinematode activities. DK, RW, and MH drafted the manuscript with input from all the authors. All authors contributed to the article and approved the submitted version.

## Funding

The work was supported by grants from the German Bioeconomy Science Center. The scientific activities of the Bioeconomy Science Center were financially supported by the Ministry of Culture and Science within the framework of the NRW Strategieprojekt BioSC (no. 313/323-400-00213). Parts of this work were funded by the state of NRW in the project RhamnoLizer. This work was supported by the Open Access Publication Fund of the University of Bonn.

## Conflict of interest

The authors declare that the research was conducted in the absence of any commercial or financial relationships that could be construed as a potential conflict of interest.

## Publisher’s note

All claims expressed in this article are solely those of the authors and do not necessarily represent those of their affiliated organizations, or those of the publisher, the editors and the reviewers. Any product that may be evaluated in this article, or claim that may be made by its manufacturer, is not guaranteed or endorsed by the publisher.
