# Supplementary material for: Production of tailored hydroxylated prodiginine showing combinatorial activity with rhamnolipids against plant-parasitic nematodes
Source: Front Microbiol. 2023 May 2;14:1151882. doi: 10.3389/fmicb.2023.1151882 (PMC10187637; doi:10.3389/fmicb.2023.1151882)
Supplement: Supplementary file 1 [file Data_Sheet_1.PDF]

Supplementary Material

Production of tailored hydroxylated prodiginine showing combinatorial activity with rhamnolipids against plant parasitic nematodes

D. F. Kossmann[†,1], M. Huang[†,2], R. Weihmann[†,3], X. Xiao[2], F. Gätgens[3], T. M. Weber[1], H. U. C. Brass[1], N. L. Bitzenhofer[3], S. Ibrahim[3], K. Bangert[3], L. Rehling[2], C. Mueller[4], T. Tiso[4], L. M. Blank[4], T. Drepper[3], K.-E. Jaeger[3,5], F. M. W. Grundler[2], J. Pietruszka*[,‡,1,5], A. S. S. Schleker*[,‡,2], A. Loeschcke*[,‡,3]

[1] Institute of Bioorganic Chemistry, Heinrich Heine University Düsseldorf, Forschungszentrum Jülich, Jülich, Germany.
[2] INRES Molecular Phytomedicine, University of Bonn, Bonn, Germany.
[3] Institute of Molecular Enzyme Technology, Heinrich Heine University Düsseldorf, Forschungszentrum Jülich, Jülich, Germany.
[4] iAMB – Institute of Applied Microbiology, ABBt – Aachen Biology and Biotechnology, RWTH Aachen University, Aachen, Germany
[5] Institute of Bio- and Geosciences (IBG-1): Biotechnology, Forschungszentrum Jülich GmbH, Jülich, Germany
*** Correspondence:** J. Pietruszka, j.pietruszka@fz-juelich.de; A. S. S. Schleker, sylvia.schleker@uni-bonn.de; A. Loeschcke, a.loeschcke@fz-juelich.de.
[†] These authors have contributed equally to this work and share first authorship.
[‡] These authors have contributed equally to this work and share last authorship.

Content

I Development of an enhanced *P. putida* mutasynthesis chassis and optimization of mutasynthesis — **Page**

Table S1 Used plasmids and oligonucleotides 2
Figure S1 Schematic comparison of the MBC-providing *P. putida* chassis pig-r2 Δ*pigD* and MBC18 3
Figure S2 Mutasynthesis of norprodigiosin from biosynthesized HBC and supplemented MAP 4
Figure S3 Hypothetical interplay of late prodigiosin biosynthetic enzymes of *S. marcescens* W838 5
Figure S4 Remaining MBC in MBC18/pVLT33-pigC-pigB in mutasynthesis with MAP 6

II Chemical synthesis of pyrroles and preparation of prodiginines
General methods and analytics 7
Procedure for hydroxylation of Pyrroles with terminal double bond 11
General synthesis procedure of Prodiginines 12
Table S2 Optimization of preparative mutasynthesis 14
Table S3 Dilution series for calculation of molar extinction coefficients 15
Figure S5 Calculation of molar extinction coefficients 16
Analytical data 17

III Impact of prodiginines on the plant-parasitic nematode *Heterodera schachtii*
Table S4 List of concentrations used for treatments 27
Table S5 List of treatments and concentrations used in the combinatorial assay 28
Figure S6 EC_{50} determination of hydroxylated prodiginines (**2**,**13**) on *Heterodera schachtii* 29
Figure S7 EC_{50} determination of di-rhamnolipids (**4**) on *Heterodera schachtii* 30
Figure S8 Dose-effect curves and combination index plots of prodiginines and di-rhamnolipids 31

I Development of an enhanced *P. putida* mutasynthesis chassis and optimization of mutasynthesis

Supplementary Table S1. Used plasmids and oligonucleotides.

**Plasmids**		
Name	Relevant characteristics	Reference
pPIG	pUC19, AmpR, *S. marcescens* W838 *pig* gene cluster	(Loeschcke et al., 2013)
pRcExpII2-YF1-FixJ-PFixK2-LacZ	pRcExpII-2, *yf1-fixJ*, P_{fixK2}, *lacZ*, KmR, CmR	(Weihmann et al., 2020)
yTREX	yCP50-poly derived, pMB1 ori, KmR, URA3, mob/oriT, *CEN4*/ARS1, transposon Tn5 *tnp*, OE-L/R, TcR	(Domröse et al., 2017)
yTREX-MBC-lacZ	yTREX, KmR, *pigAFGHIJKLMN-lacZ*, TcR	This study
yTREX-HBC-pigC-lacZ	yTREX, KmR, *pigACGHIJKLM-lacZ*, TcR	This study
pVLT33-pigC	pVLT33 (de Lorenzo et al., 1993), KmR, $LacI^q$/P_{tac}, *pigC*	(Brands et al., 2020)
pVLT33-pigC-pigB	pVLT33, KmR, $LacI^q$/P_{tac}, *pigC-pigB*	This study

**Oligonucleotides**		
Name	Sequence	Application
Cloning of yTREX-MBC-lacZ		
AD142	AGAAATATTAGCTAATTTAATCTCTCAACCCGATGGCGGAGTTCGTCATGG	PCR *pigA*
AD171	CATTGCTGGCTCCCTAGTCTAAAAAGGTGGAGAGTCATGCGCCTCCCCGCAGAC	PCR *pigA*
AD172	GTCTGCGGGGAGGCGCATGACTCTCCACCTTTTTAGACTAGGGAGCCAGCAATG	PCR *pigFGHI*
AD162	GTTGGACAGATCGATACGATCGATGCCGGGCAGTGCGCAG	PCR *pigFGHI*
AD163	CTGCGCACTGCCCGGCATCGATCGTATCGATCTGTCCAAC	PCR *pigJKLMN*
AD164	GTAATCATGGTCATAGCTGTTTCCTGTGTGTTACAGCACGAAAGGAATGAAACATTTAAC	PCR *pigJKLMN*
AD124	CACACAGGAAACAGCTATGACCATGATTACGGATTCACTGGCCGTCGTTTTAC	PCR *lacZ*
AD125	GAAACAGCTATGACCATGATTACGCCAAGCTAGCGCTTATTTTTGACACCAGACC	PCR *lacZ*
Cloning of yTREX-HBC-pigC-lacZ		
AD142	AGAAATATTAGCTAATTTAATCTCTCAACCCGATGGCGGAGTTCGTCATGG	PCR *pigA*
AD143	CATCACCTCCGCAGGCGTAACGGGCATTGCTCATGCGCCTCCCCGCAGAC	PCR *pigA*
AD144	GTCTGCGGGGAGGCGCATGAGCAATGCCCGTTACGCCTGCGGAGGTGATG	PCR *pigC*
AD145	TGCGAGCTCCCGTTGCTAACTGGTCTAGTTCTAGCCATCGGCACGTTCTCCGCGTTG	PCR *pigC*
AD146	CGCGGAGAACGTGCCGATGGCTAGAACTAGACCAGTTAGCAACGGGAGCTCG	PCR *pigGHIJKLM*
AD158	GTAATCATGGTCATAGCTGTTTCCTGTGTGTCCTCAGCGGATTAGGGGGAATCG	PCR *pigGHIJKLM*
AD124	CACACAGGAAACAGCTATGACCATGATTACGGATTCACTGGCCGTCGTTTTAC	PCR *lacZ*
AD125	GAAACAGCTATGACCATGATTACGCCAAGCTAGCGCTTATTTTTGACACCAGACC	PCR *lacZ*
Cloning of pVLT33-pigC-pigB		
RW144	GTCTGCGGGGAGGCGCATGAGCAATGCCCGTTACGCCTGCGGAGGTGATG	PCR *pigB*
RW145	TGCGAGCTCCCGTTGCTAACTGGTCTAGTTCTAGCCATCGGCACGTTCTCCGCGTTG	PCR *pigB*
qPCR primers		
AD153	TACCTGATAGGCACGCTGTT	PCR *pigN*
AD154	TTGTTCGGATCCTGTTTGAA	PCR *pigN*
AD155	TCGCCAAGAAGTACACCAAC	PCR *rpoD*
AD156	TTTCATCAGACCGATGTTGC	PCR *rpoD*

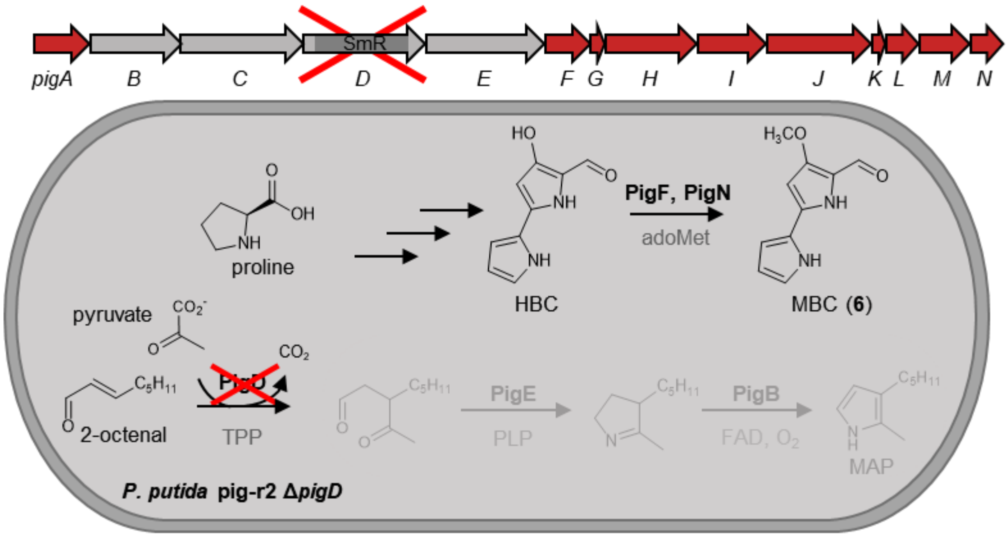

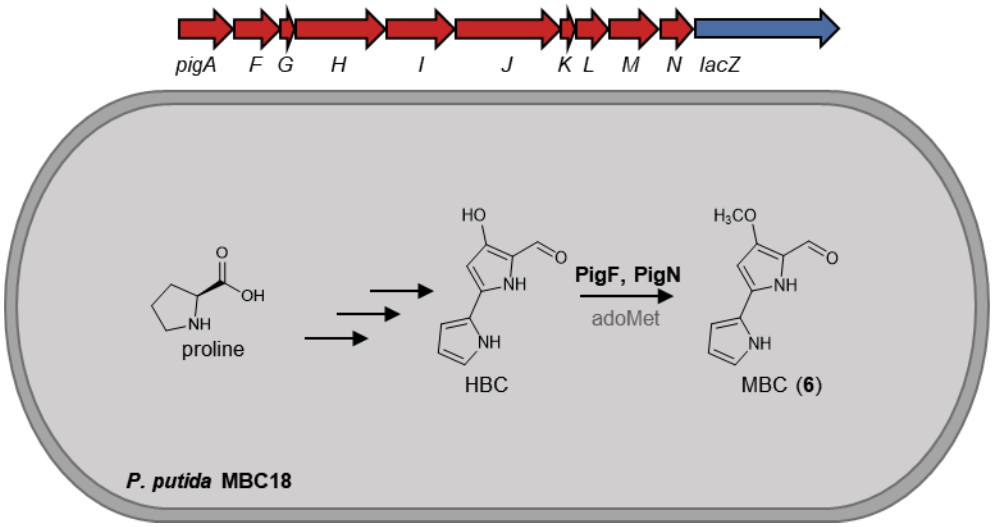

Supplementary Figure S1. Schematic comparison of the MBC-providing *P. putida* chassis pig-r2 Δ*pigD* and MBC18. Chromosomally integrated genes are shown above cells; precursor biosynthetic pathways are schematically shown in cells. **Upper part, *P. putida* pig-r2 Δ*pigD*:** MBC biosynthetic genes are shown in red, MAP biosynthetic genes and ligase-encoding *pigC* are marked in grey. The deletion of *pigD* by integration of a resistance cassette (Klein et al., 2017) is indicated. **Lower part, *P. putida* MBC18:** MBC biosynthetic genes are shown in red, β-galactosidase-encoding *lacZ* is marked in blue. Note that *P. putida* pig-r2 Δ*pigD* is a readily functional mutasynthesis chassis as it also expresses PigC (not shown in the cell), while in MBC18, an additional plasmid for expression of *pigC* and *pigB* has to be introduced.

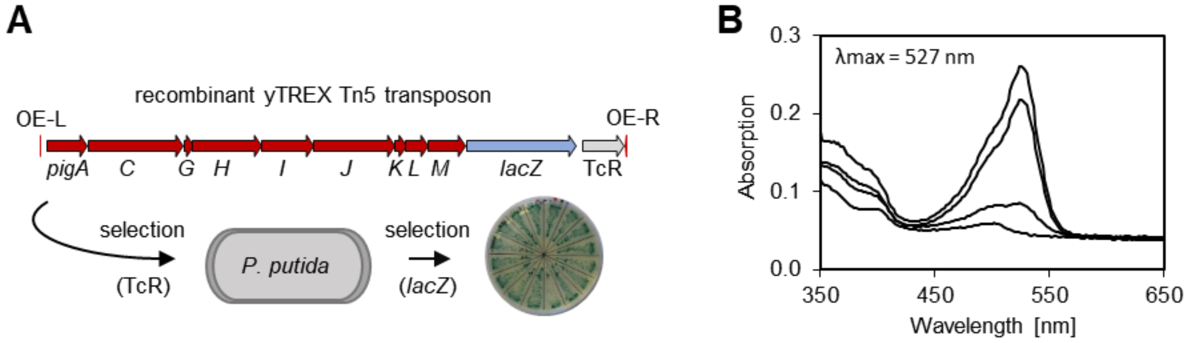

Supplementary Figure S2. Mutasynthesis of norprodigiosin from biosynthesized HBC and supplemented MAP.

(A) The HBC-pigC strain was generated via an analogous procedure to MBC strain construction: *pigA*, *pigC*, and *pigGHIJKLM* were PCR-amplified (primers AD142+AD143, AD144+AD145 and AD146+AD158, respectively) using vector pPIG (Loeschcke et al., 2013) as template. Thereby, *pigNF* and all MAP biosynthetic genes were excluded. In addition, *lacZ* was amplified as promotorless gene (primers AD124+125, 3127 bp) using vector pRcExpII2-YF1-FixJ-PFixK2-LacZ (Weihmann et al., 2020) as template. All genes were amplified with the respective 5'-UTR sequences including the ribosome binding sites; primers added homology arms to each of the fragments. The synthetic gene cluster was assembled into the yTREX vector, which was used for conjugational transfer into *P. putida* KT2440. Clones with integrated recombinant yTREX transposon were selected on LB agar plates containing 50 µg/mL tetracycline. Expressing clones were identified by blue color due to X-gal conversion by *lacZ*-encoded β-galactosidase.

(B) Precultures (1 mL LB medium in Flowerplates) were used to inoculate 1 mL test cultures with an OD_{650} of 0.05 in LB medium. After a 5 h incubation (30 °C, 1200 rpm), 150 µL samples were transferred to a microtiter plate, supplemented with 50 µM MAP, and incubated for 18 h (30 °C, 600 rpm), before metabolites were extracted with acidified ethanol (4% 1 M HCL) and absorption spectra were measured with a plate reader (infinite M1000 PRO, Tecan). Results of quadruplicate measurements showed orange coloration and a characteristic prodiginine absorption (λmax = 527 nm) with extremely varying intensity, which we assigned to norprodigiosin based on previously published data (Mody et al., 1990; Williamson et al., 2005). Samples were additionally analysed via LC-MS as described for MBC strains, which showed specific signals corresponding to the ion [M+H]+ of norprodigiosin (310 m/z), at 8,0 min (data not shown). Since the results were difficult to reproduce, this strategy, which aimed at obtaining norprodigiosin derivatives via mutasynthesis, was not further pursued. However, results were of interest because they indicated that a conversion of HBC and MAP is generally possible in the HBC strain by expression of PigC. Since HBC strains only lack PigN/F compared to MBC strains, this finding pointed to PigN/F completely prohibiting PigC-mediated condensation of MBC and MAP in the MBC strains.

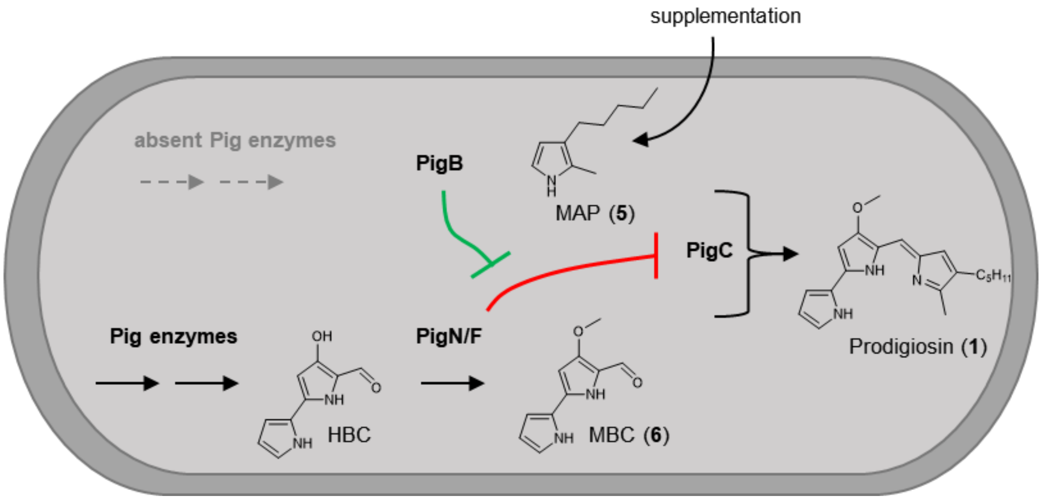

Protein	Function	Putative membrane association*
PigB	Flavin-dependent amine oxidase	3 predicted TMHs, experimental indication (Couturier et al., 2019)
PigC	prodigiosin synthesizing transferase	no predicted TMHs, experimental indication (Chawrai et al., 2012; Brands et al., 2020)
FigF	SAM-dependent O-methyl transferase	no predicted TMHs, potential association to membrane via PigN (Williamson et al., 2005)
PigN	Unknown function in HBC → MBC conversion (Williamson et al., 2005)	6 predicted TMHs, experimental indication (Williamson et al., 2005)

Supplementary Figure S3. Hypothetical interplay of late prodigiosin biosynthetic Pig enzymes of *S. marcescens* W838. Depicted are our summarized results in this regard: **(i)** PigC by itself is sufficient for condensation of MAP and MBC to prodigiosin. **(ii)** In the absence of MAP biosynthetic enzymes, we supplemented MAP (or analoga) for mutasynthesis (indicated in purple). In this setup, we found the presence of PigN/F to prohibit condensation (indicated in red). **(iii)** In the depicted scenario, only the additional expression of PigB restored condensation (abolishment of the PigN/F-associated inhibition is indicated in green).

Both, the last precursor biosynthetic enzymes and the final ligase PigC are thought to be membrane-associated: While PigC lacks sequence features allowing transmembrane domain prediction, activity is often investigated by utilization of membrane fractions from expression cultures (Chawrai et al., 2012; Brands et al., 2020). PigN has predicted transmembrane helices and mutations in these regions are particularly detrimental to pigment formation (Williamson et al., 2005). Its involvement in the conversion of HBC to MBC by a methyl transfer has been shown and since the actual methyl transferase is clearly PigF (which has no predicted transmembrane domains), a function in protein assembly at the membrane was postulated (Williamson et al., 2005, 2006). Another study recently showed that in a Δ*pigB* mutant of *Serratia* sp. ATCC 39006, the pigmentation phenotype could not be recovered by expression of a PigB version with truncated membrane anchor (Couturier et al., 2019). Only faint recovery was observed with HapB complementation, which is the functional equivalent of PigB in *Hahella chejuensis* but lacks the membrane anchor. This could further point to an essential interplay of the late prodigiosin biosynthetic Pig enzymes of *Serratia* that takes place at the membrane.

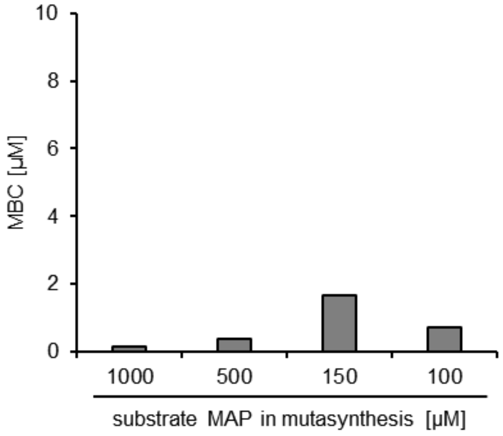

Supplementary Figure S4. Remaining MBC in MBC18/pVLT33-pigC-pigB in mutasynthesis with different MAP concentrations. MBC levels in mutasynthesis cultures were estimated by analysis of crude extracts from PU foam cubes (after recovery from preparative mutasynthesis cultures) via HPLC-PDA.

II Chemical synthesis of pyrroles and preparation of prodiginines

1 General methods and analytics

Mass spectrometry: The reactions were monitored by GS-MS on a Thermo Scientific Frace 1310 Gas Chromatography (*Thermo Scientific*, Waltham, MA, USA) coupled to an ISQ™ QD Single Quadrupole Mass Spectrometer (*Thermo Scientific*, Waltham, MA, USA). For chromatographic separation, an Optima 5MS column (30 m x 0.25 mm, 0.25 µm; *Macherey-Nagel*, Düren, Germany) was used as stationary phase and helium as carrier gas. The measurement followed the following temperature program: 60 °C (1 min), 60−185 °C (15 °C/min), 185−280 °C (120 °C/min), 280 °C (5 min); temperature injector: 250 °C; temperature detector: 230 °C. The ionization method used was electron impact ionization at 70 eV. Fractionation patterns were measured on an Atmospheric Solid Analysis Probe (ASAP) Mass Spectrometer (Model: *Expression Compact*, Advion, New York, USA). Atmospheric pressure chemical ionization (APCI) was used as the ionization method. High-resolution mass spectra (HRMS) were measured at Heinrich Heine University Düsseldorf on a UHR-QTOF maXis 4G (*Bruker*, Massachusetts, USA) using electron spray ionization (ESI).

NMR spectroscopy: For structural elucidation, an NMR data set of all chemical compounds was recorded on the Advance/DRX 600 NMR spectrometer (*Bruker*, Massachusetts, USA). The samples were measured at ambident temperature in $CDCl_3$ at 600 MHz and 151 MHz, respectively. The chemical shift is given in ppm relative to tetramethylsilane [^{1}H: δ($SiMe_4$) = 0.00 ppm] as an internal standard or relative to the solvent reference signal [^{1}H: δ($CDCl_3$) = 7.26 ppm; ^{13}C: δ($CDCl_3$) = 77.16 ppm]. The multiplicities were given as follows: singlet (s), doublet (d), triplet (t), quartet (q), multiplet (m), centred multiplet (m_c) and broad singlet (brs). Quantitative NMR analysis was performed in triplicates with 1,3,5-trimethoxybenzene (*Sigma-Aldrich*) as internal standard.

IR spectroscopy: IR date were recorded on a *SpectrumTwo* ATR-IR instrument (*Perkin Elmer*, Hamburg, Germany). Liquids were measured in pure form. Solids were dissolved in chloroform or dichloromethane and measured after evaporation of the solvent. Absorbance frequencies are reported in cm^{-1}.

2 Synthesis of 2,3-disubstituted pyrroles (11)

The pyrroles **11** were synthesised in a four-step synthesis sequence starting from the according carboxylic acid. The synthesis conditions were adapted from literature (Klein et al., 2017).

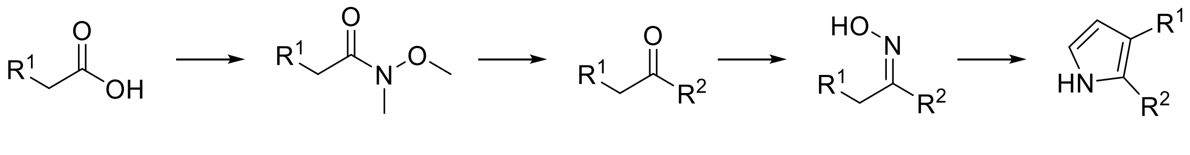

7 **8** **9** **10** **11**

N-Methoxy-N-methylhept-6-enamid (8b)

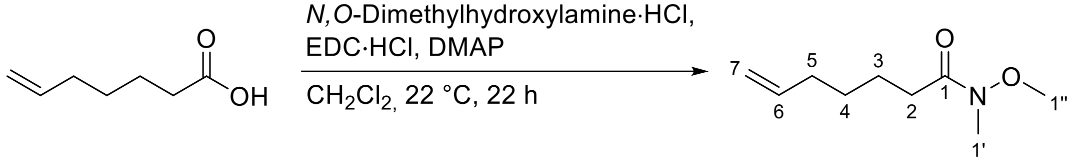

7b **8b**

To a solution of 6-heptenoic acid (3.30 g, 25.8 mmol, 1.0 eq.) in dichloromethane (90.3 mL, 3.5 mL/mmol carboxylic acid) were added N,O-dimethylhydroxylamine hydrochlorid (2.36 g, 38.6 mmol, 1.50 eq.), N-(3-dimethylaminopropyl)-N'ethylcarbodiimid hydrochlorid (6.00 , 38.6 mmol, 1.5 eq.) and 4-(dimethylamino)pyridine (4.72 g, 38.6 mmol, 1.5 eq.). After stirring for 22 h at 22 °C, the reaction mixture was quenched with a saturated solution of NaCl and extracted with dichloromethane (3 x 50 mL). The combined organic layers were first washed with 1 N HCl, afterwards with saturated $NaHCO_3$ solution and dried over $MgSO_4$. After removal of the solvent under reduced pressure N-Methoxy-N-methylhept-6-enamide (**8b**, 4.20 g, 24.5 mmol, 95%) was obtained as a yellow oil and was used for the following experiment without further purification. **^{1}H-NMR** (600 MHz, $CDCl_3$): δ [ppm] = 1.44 (m$_c$, 2H, 4-H), 1.65 (m$_c$, 2H, 3-H), 2.08 (m$_c$, 2H, 5-H), 2.42 (t, $^3J_{2,3}$ = 7.6 Hz, 2H, 2-H), 3.18 (s, 3H, 1″-H), 3.68 (s, 3H, 1′-H), 4.94 (dd, $^{cis,3}J_{7a,6}$ = 10.2 Hz, $^2J_{7a,7b}$ = 1.2 Hz, 1H, 7-H$_a$), 5.01 (dd, $^{trans,3}J_{7b,6}$ = 17.2 Hz, $^2J_{7b,7a}$ = 1.7 Hz, 1H, 7-H$_b$), 5.81 (ddt, $^{trans,3}J_{6,7b}$ = 16.9 Hz, $^{cis,3}J_{6,7a}$ = 10.2 Hz, $^3J_{6,5}$ = 6.7 Hz, 1H, 6-H); **^{13}C-NMR** (151 MHz, $CDCl_3$): δ [ppm] = 24.2 (C-3), 28.8 (C-4), 31.7 (C-2), 31.8 (C-1″) 33.6 (C-5) 61.3 (C-1′), 114.6 (C-7), 138.7 (C-6). **IR** (ATR-film): $\tilde{\nu}$ [1/cm] = 3074, 2936, 2861, 1663, 1414, 1384, 1177, 1118, 993, 910, 497; **MS** (APCI): m/z = 172 [(M)$^+$], 123, 83, 55.

Oct-7-en-2-one (9b)

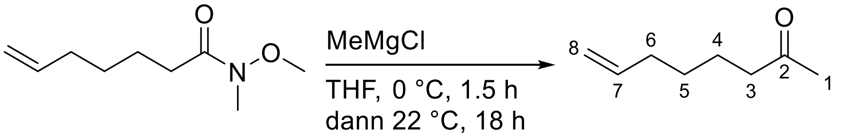

8b **9b**

N-Methoxy-N-methylhept-6-enamide (**8b**, 2.80 g, 16.4 mmol, 1.0 eq.) was dissolved in dry THF (100 mL) and methylmagnesium chloride (3 M in diethyl ether, 16.4 mL, 49.1 mmol, 3.0 eq.) was added within 15 min at 0 °C. The reaction mixture was stirred for 1.5 h at 0 °C and afterwards quenched by the addition of a saturated solution of NH_4Cl at 0 °C and extracted with dichloromethane (3 x 60 mL). The combined organic layers were dried over $MgSO_4$ and the solvent was evaporated under reduced pressure to yield oct-7-en-2-one (**8b**, 1.63 g, 11.6 mmol, 94%) as a yellowish oil without further purification. **^{1}H-NMR** (600 MHz, $CDCl_3$): δ [ppm] = 1.39 (tt, $^3J_{5,4}$ = 9.5 Hz, $^3J_{5,6}$ = 6.8 Hz, 2H, 5-H), 1.56–1.62 (m, 2H, 4-H), 2.06 (dt, $^3J_{6,5}$ = 7.3 Hz, $^3J_{6,7}$ = 7.0 Hz, 2H, 6-H), 2.13 (s, 3H, 1-H), 2.43 (t, $^3J_{3,4}$ = 7.4 Hz, 2H, 3-H), 4.95 (dd, $^{cis,3}J_{8a,7}$ = 10.2 Hz, $^2J_{8a,8b}$ = 1.2 Hz, 1H, 8-H$_a$), 5.01 (dd, $^{trans,3}J_{8b,7}$ = 17.1 Hz, $^2J_{8b,8a}$ = 1.7 Hz, 1H, 8-H$_b$), 5.79 (ddt, $^{trans,3}J_{7,8b}$ = 17.0 Hz, $^{cis,3}J_{7,8a}$ = 10.2 Hz, $^3J_{7,6}$ = 6.7 Hz, 1H, 7-H); **^{13}C-NMR** (151 MHz, CDCl3): δ [ppm] = 23.4 (C-4), 28.5 (C-5), 30.0 (C-6), 33.5 (C-1), 43.7 (C-3), 114.8 (C-8), 138.6 (C-7), 209.2 (C-2); **IR** (ATR-film): $\tilde{\nu}$ [1/cm] = 3074, 2934, 2861, 1717, 1601, 1363, 1217, 992, 913; **MS** (APCI): m/z = 123, 109.

General synthesis procedure for oximes (10)

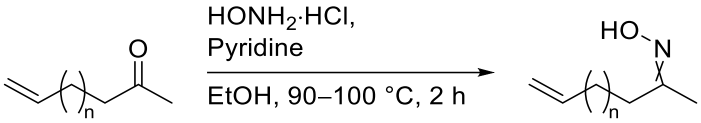

9b,c **10b,c**

To a solution of the ketone (9b,c, 1.0 eq.) in undenatured EtOH (0.5 mL/mmol ketone) was added pyridine (0.8 eq.) and ground hydroxylamine hydrochloride (1.5 eq.). The reaction mixture was heated for 2 h at 90−100 °C under reflux and afterwards extracted with dichloromethane (3 x 25 mL). The combined organic layers were washed with 1 N HCl (3 x 20 mL) and then dried over $MgSO_4$. After removal of the solvent under reduced pressure, the oxime (**10b,c**) was obtained as *E*/*Z* isomer mixture without further purification.

Oct-7-enone oxime (10b)

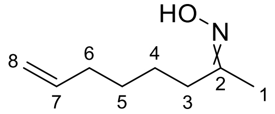

10b

According to the general procedure for the synthesis of oximes, the oxime **10b** was prepared from oct-7-en-2-one (**9b**, 1.42 g, 11.3 mmol) and hydroxylamine hydrochloride (1.17 g, 16.9 mmol) and was obtained as a yellow oil (1.40 g, 10.1 mmol, 88%). **^{1}H-NMR** (600 MHz, $CDCl_3$): δ [ppm] = 1.38−1,44 (m, 2H, 5-H), 1.50−1.60 (m, 2H, 4-H), 1.91 (s, 3H, 1-H), 2.07 (dt, $^3J_{6,5}$ = 7.8 Hz, $^3J_{6,7}$ = 7.8 Hz, 2H, 6-H), 2.24 (t, $^3J_{3,4}$ = 7.5 Hz, 2H, 3-H), 4.93−4.97 (m, 1H, 8-H$_a$), 5.01 (dd, $^{trans,3}J_{8b,7}$ = 17.1 Hz, $^2J_{8b,8a}$ = 1.7 Hz, 1H, 8-H$_b$), 5.78 (m, 1H, 7-H); **^{13}C-NMR** (151 MHz, $CDCl_3$): δ [ppm] = 13.6 (C-1), 25.8 (C-4), 28.4 (C-5), 33.5 (C-6), 35.4 (C-3), 114.8 (C-8), 138.6 (C-7); **IR** (ATR-Film): $\tilde{\nu}$ [1/cm] = 2971, 2922, 2861, 1735, 1443, 1363, 1217, 901, 748; **MS** (APCI): *m*/*z* = 142 [(M)$^+$], 115, 109.

Dec-9-enone oxime (10c)

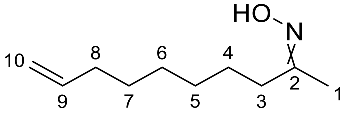

10b

According to the general procedure for the synthesis of oximes, oxime **10c** was prepared from commercial available dec-9-en-2-one (**9c**, 4.34 g, 28.1 mmol) and hydroxylamine hydrochloride (2.93 g, 42.2 mmol) and was obtained as a yellow oil (4.70 g, 27.7 mmol, 99%). **^{1}H-NMR** (600 MHz, $CDCl_3$): δ [ppm] = 1.31−1.37 (m, 4H, 6-H, 7-H), 1.37−1.43 (m, 2H, 5-H), 1.49−1.57 (m, 2H, 4-H), 1.89 (s, 3H, 1-H), 2.06 (dt, $^3J_{8,7}$ = 7.1 Hz, $^3J_{8,9}$ = 7.1 Hz, 2H, 8-H), 2.19 (t, $^3J_{3,4}$ = 7.6 Hz, 2H, 3-H), 4.91−4.97 (m, 1H, 10-H$_a$), 5.01 (dd, $^{trans,3}J_{10b,9}$ = 17.2 Hz, $^2J_{10b,10a}$ = 1.7 Hz, 1H, 10-H$_b$), 5.83 (ddt, $^{trans,3}J_{9,10b}$ = 16.9 Hz, $^{cis,3}J_{9,10a}$ = 10.0 Hz, $^3J_{9,8}$ = 6.7 Hz, 1H, 9-H); **^{13}C-NMR** (151 MHz, $CDCl_3$): δ [ppm] = 13.4 (C-1), 26.3 (C-4), 28.9 (C-5), 29.0 (C-6), 29.2 (C-7), 33.9 (C-8), 35.9 (C-3), 114.4 (C-10), 139.2 (C-9), 159.1 (C-2); **IR** (ATR-film): $\tilde{\nu}$ [1/cm] = 3081, 2927, 1641, 1463, 1368, 993, 909; **MS** (APCI): *m*/*z* = 170 [(M)$^+$], 168, 152. **HRMS** (ESI, positive ion): calculated for $C_{10}H_{20}NO$ (M + H)$^+$ = 170.1539, found = 170.1540.

General synthesis procedure for pyrroles (11)

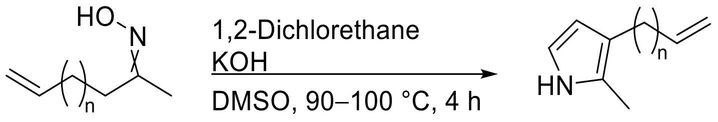

10b,c **11c,d**

To a solution of oxime (**10b,c**, 1.0 eq.) in DMSO (2.14 mL/mmol oxime) was added ground KOH (5.0 eq.) and water (0.75 eq.). A solution of 1,2-dichloroethane (3.5 eq.) in DMSO (0.21 mL/mmol oxime) was added at 95 °C over a period of 2 h by a syringe pump. After 1 h addition, again KOH (5.0 eq.) was added. After a total reaction time of 4 h at 90−100 °C, the reaction was cooled to 23 °C, ice water (20 mL) was added and extracted with diethyl ether (3 x 20 mL). The combined organic layers were dried over $MgSO_4$ and the solvent evaporated under reduced pressure. Subsequent column chromatographic separation [silica gel, PE/AcOEt (80:20) + 1% (*v*/*v*) trimethylamine] allowed excess starting material to be removed and the pyrrole (**11b,c**) was subsequently isolated as a colourless oil via a ball tube distillation (1 mbar, 70−80 °C). The reaction was monitored via thin layer chromatography, GC-MS and ^{1}H NMR. The product was stored under argon atmosphere at −20 °C.

2-Methyl-3-(pent-4-en-1-yl)-1*H*-pyrrole (11c)

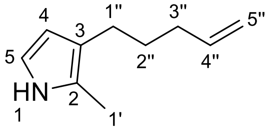

11c

According to the general procedure for the synthesis of pyrroles, the pyrrole **11c** was prepared from oct-7-ene-2-one oxime (**10b**, 1.59 g, 11.2 mmol) and obtained as a colourless oil (863.0 mg, 5.78 mmol, 51%). In addition the side products *N*-vinyl pyrrole (207 mg, 1.18 mmol, 11%) and oxime diether (217 mg, 0.70 mmol, 6%) were isolated. **^{1}H-NMR** (600 MHz, $CDCl_3$): δ [ppm] = 1.63 (tt, $^3J_{2'',1''}$ = 7.6 Hz, $^3J_{2'',3''}$ = 7.6 Hz, 2H, 2″-H), 2.02−2.14 (m, 2H, 3″-H), 2.18 (s, 3H, 1′-H), 2.40 (t, $^3J_{1'',2''}$ = 7.8 Hz, 2H, 1″-H), 4.95 (dd, $^{cis,3}J_{5a'',4''}$ = 10.2 Hz, $^2J_{5a'',5b''}$ = 1.3 Hz, 1H, 5″-H_a), 5.02 (dd, $^{trans,3}J_{5b'',4''}$ = 17.1 Hz, $^2J_{5b'',5a''}$ = 1.8 Hz, 1H, 5″-H_b), 5.85 (ddt, $^{trans,3}J_{4'',5b''}$ = 16.9 Hz, $^{cis,3}J_{4'',5a''}$ = 10.2 Hz, $^3J_{4'',3''}$ = 6.6 Hz, 1H, 4″-H), 6.01 (dd, $^4J_{4,1}$ = 2.8 Hz, $^3J_{4,5}$ = 2.8 Hz, 1H, 4-H), 6.59 (dd, $^3J_{5,1}$ = 2.7 Hz, $^3J_{5,4}$ = 2.7 Hz, 1H, 5-H), 7.71 (brs, 1H, 1-NH); **^{13}C-NMR** (151 MHz, $CDCl_3$): δ [ppm] = 11.2 (C-1′), 25.5 (C-1″), 30.6 (C-2″), 33.7 (C-3″), 109.0 (C-4), 114.4 (C-5″), 115.0 (C-5), 119.4 (C-3), 123.5 (C-2), 139.3 (C-4″); **IR** (ATR-film): $\tilde{\nu}$ [1/cm] = 3381, 3074, 2971, 2928, 2855, 1637, 1443, 1108, 992, 907, 712; **MS** (APCI): *m*/*z* = 150 [(M)$^+$], 108.

2-Methyl-3-(hept-6-en-1-yl)-1*H*-pyrrole (11d)

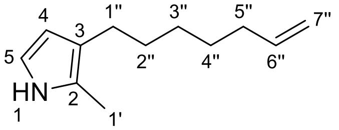

11d

According to the general procedure for the synthesis of pyrroles, the pyrrole **11d** was prepared from dec-9-ene-2-one oxime (**10c**, 3.00 g, 17.7 mmol) and was obtained as a colorless oil (935 mg, 5.3 mmol, 30%). **^{1}H-NMR** (600 MHz, $CDCl_3$): δ [ppm] = 1.44 (tt, $^3J_{3'',2''}$ = 7.5 Hz, $^3J_{3'',4''}$ = 7.5 Hz, 2H, 3″-H), 1.51−1.59 (m, 2H, 2″-H), 2.02−2.14 (m, 2H, 4″-H), 2.18 (s, 3H, 1′-H), 2.39 (t, $^3J_{1'',2''}$ = 7.6 Hz, 2H, 1″-H), 4.93 (dd, $^{cis,3}J_{6a'',5''}$ = 10.2 Hz, $^2J_{6a'',6b''}$ = 1.2 Hz, 1H, 6″-H_a), 5.00 (dd, $^{trans,3}J_{6b'',5''}$ = 17.1 Hz, $^2J_{6b'',6a''}$ = 1.7 Hz, 1H, 6″-H_b), 5.82 (ddt, $^{trans,3}J_{5'',6b''}$ = 16.9 Hz, $^{cis,3}J_{5'',6a''}$ = 10.2 Hz, $^3J_{5'',4''}$ = 6.7 Hz, 1H, 5″-H), 6.01 (dd, $^4J_{4,1}$ = 2.8 Hz, $^3J_{4,5}$ = 2.8 Hz, 1H, 4-H), 6.59 (dd, $^3J_{5,1}$ = 2.7 Hz, $^3J_{5,4}$ = 2.7 Hz, 1H, 5-H), 7.70 (brs, 1H, 1-NH); **^{13}C-NMR** (151 MHz, $CDCl_3$): δ [ppm] = 11.2 (C-1′), 25.9 (C-1″), 28.9 (C-3″), 31.0 (C-2″), 33.9 (C-4″), 109.0 (C-4), 114.3 (C-6″), 115.0 (C-5), 119.7 (C-3), 123.4 (C-2), 139.4 (C-5″); **IR** (ATR-film): $\tilde{\nu}$ [1/cm] = 3385, 2928, 2855, 1741, 1467, 1363, 1217, 907, 712; **MS** (APCI): *m/z* = 164 [$(M)^+$], 121, 108; **HRMS** (ESI, positive ion): calculated for $C_{11}H_{18}N$ $(M + H)^+$ = 164.1434, found = 164.1433.

3 Procedure for hydroxylation of pyrroles with terminal double bond

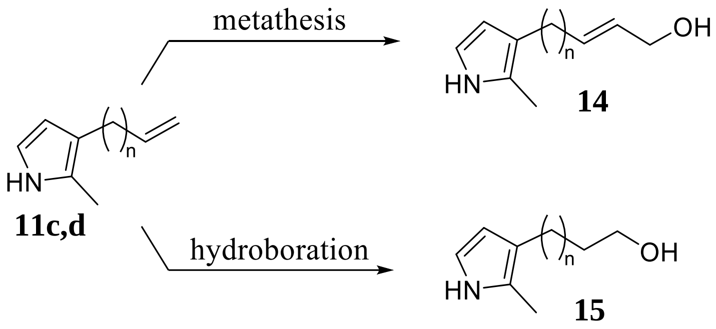

To synthesis pyrrole **14** with allyl alcohol function, a modified metathesis procedure was used(Taber and Frankowski, 2006; Habash et al., 2020). The hydroboration towards pyrrole **15** was performed following the method previously described (Aldrich, 2012).

(*E*)-6-(2-methyl-1*H*-pyrrole-3-yl)hex-2-en-1-ol (14)

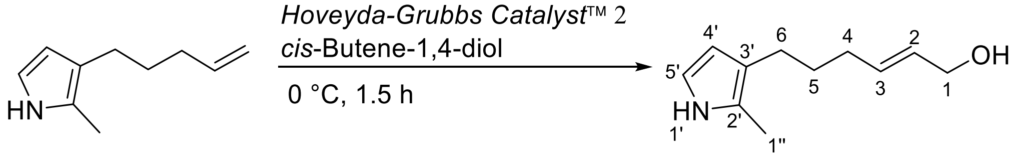

2-Methyl-3-(pent-4-en-1-yl)-1*H*-pyrrole **(11c**, 53.0 mg, 0.36 mmol, 1.0 eq.**)** and *cis*-butene-1,4-diol (125 mg, 1,41 mmol, 4.0 eq.) were dissolved in dry dichloromethane (2.5 mL). At 0 °C, *Hoveyda-Grubbs Catalyst*™ 2nd Generation (7.69 mg, 12.3 µmol, 3 mol-%) was added and the reaction was stirred for 1.5 h at 0 °C. After removal of the solvent under reduced pressure, the crude product was purified by column chromatography [silica gel; PE/AcOEt (70:30, 30−60% AcOEt gradient) + 1% (*v*/*v*) trimethylamine] to give (*E*)-6-(2-methyl-1*H*-pyrrol-3-yl)hex-2-en-1-ol (**14**, 30.0 mg, 0.17 mmol, 47%) as yellow oil. The reaction was monitored by thin layer chromatography, GC-MS and ^{1}H NMR. The products were stored under argon atmosphere at −20 °C. 0.36 mmol). **^{1}H-NMR** (600 MHz, $CDCl_3$): δ [ppm] = 1.2 (brs, 1H, 1-OH), 1.6−1.7 (m, 2H, 5-H), 2.05−2.14 (m, 2H, 4-H), 2.18 (s, 3H, 1″-H), 2.40 (t, $^3J_{6,5}$ = 7.6 Hz, 2H, 6-H), 4.09 (d, $^3J_{1,2}$ = 5.7 Hz, 2H, 1-H), 5.69 (m_c,

2H, 3-H, 2-H), 6.00 ($^4J_{4',1'}$ = 2.8 Hz, $^3J_{4',5'}$ = 2.8 Hz, 1H, 4′-H), 6.59 (dd, $^3J_{5',1'}$ = 2.7 Hz, $^3J_{5',4'}$ = 2.7 Hz, 1H, 5′-H), 7.72 (brs, 1H, 1′-NH); **^{13}C-NMR** (151 MHz, $CDCl_3$): δ [ppm] = 11.2 (C-1″), 25.5 (C-6), 30.7 (C-5), 32.1 (C-4), 64.0 (C-1), 109.0 (C-4′), 115.0 (C-5′), 119.1 (C-3′), 123.3 (C-2′), 129.1 (C-3), 133.7 (C-2); **IR** (ATR-film): $\tilde{\nu}$ [1/cm] = 3373, 2922, 2855, 1741, 1443, 1363, 1089, 974, 706; **MS** (APCI): *m/z* = 180 [(M)$^+$], 162, 120; **HRMS** (ESI, positive ion): calculated for $C_{11}H_{18}NO$ (M + H)$^+$ = 180.1383, found = 180.1382.

7-(2-methyl-1*H*-pyrrole-3-yl)heptan-1-ol (15)

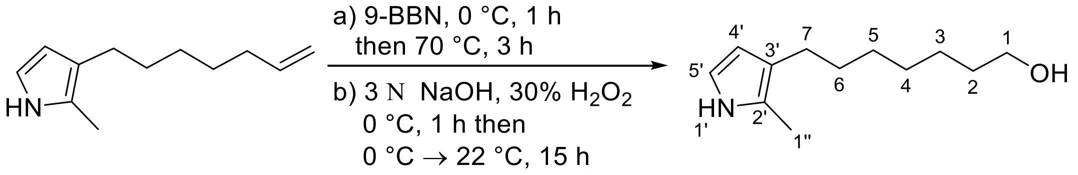

11d **15**

9-BBN (0.5 N in THF, 2.02 g, 1.13 mmol, 2.2 eq.) was added to a solution of 3-(hept-6-en-1-yl)-2-methyl-1*H*-pyrrole (**11d**, 91.0 mg, 0.51 mmol, 1.0 eq.) in dry THF (4.2 mL) over a period of 15 min at 0 °C. After stirring for 1 h at 0 °C, the reaction mixture was heated up at 70 °C under reflux for 3 h. Subsequently an aqueous solution of 3 N NaOH (866 mg, 855 µL, 2.57 mmol, 5.0 eq.) and 30% H_2O_2 (815 mg, 731 µL, 7.19 mmol, 14.0 eq.) was added at 0 °C. After 1 h at 0 °C the reaction mixture was allowed to reach room temperature and was stirred for further 15 h at 22 °C. Ice water (40 mL) was added and the mixture was extracted with dichloromethane (3 x 50 mL). The combined organic layers were dried over $MgSO_4$, the solvent was evaporated under reduced pressure and the crude product was purified by column chromatography on silica gel [PE:ethyl acetate (70:30 to 50:50) + 1% (*v*/*v*) trimethylamine] to isolate 7-(2-methyl-1*H*-pyrrole-3-yl)heptan-1-ol (**15**, 49 mg, 0.25 mmol, 50%) as orange oil. **^{1}H-NMR** (600 MHz, $CDCl_3$): δ [ppm] = 1.18 (brs, 1H, 1-OH), 1.30−1.35 (m, 6H, 3-H, 4-H, 5-H), 1.48−1.60 (m, 4H, 2-H, 6-H), 2.18 (s, 3H, 1″-H), 2.38 (t, $^3J_{7,6}$ = 7.6 Hz, 2H, 7-H), 3.63 (t, $^3J_{1,2}$ = 7.6 Hz, 2H, 1-H), 6.00 (dd, $^4J_{4',1'}$ = 2.9 Hz, $^3J_{4',5'}$ = 2.8 Hz, 1H, 4′-H), 6.59 (dd, $^3J_{5',1'}$ = 2.7 Hz, $^3J_{5',4'}$ = 2.7 Hz, 1H, 5′-H), 7.72 (brs, 1H, 1′-NH); **^{13}C-NMR** (151 MHz, $CDCl_3$): δ [ppm] = 11.2 (C-1″), 25.8 (C-7), 26.0 (C-5), 29.5 (C-4), 29.6 (C-3), 31.4 (C-6), 33.0 (C-2), 63.3 (C-1), 109.0 (C-4′), 115.0 (C-5′), 119.8 (C-3′), 123.3 (C-2′); **IR** (ATR-film): $\tilde{\nu}$ [1/cm] = 3373, 2927, 2855, 1741, 1686, 1467, 1369, 1053, 712; **MS** (APCI, positive ion): *m/z* = 196 [(M)$^+$], 178; **HRMS** (ESI, positive ion): calculated for $C_{12}H_{22}NO$ (M + H)$^+$ = 196.1696, found = 196.1697.

4 General synthesis procedure of prodiginines

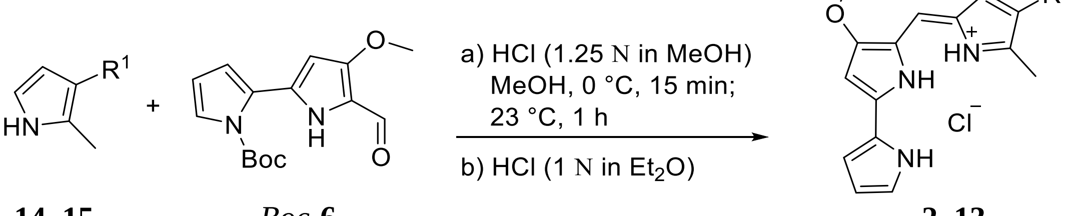

14, 15 *Boc*-**6** **2, 13**

The chemical condensation reaction towards the prodiginines **2** and **13** was performed in a modified reaction procedure (Brass et al., 2019).

To a solution of *Boc*-**6** (1.0 eq.) in dry methanol (20 mL/mmol *Boc*-**6**) was added a solution of the corresponding pyrrole (**14**, **15**, 1.0 eq.) in dry methanol (6 mL/mmol *Boc*-MBC), as well as a HCl in methanol solution (1.25 M, 1.3 eq.) at 0 °C in parallel via two syringes. After stirring for 15 min at 0 °C, the dark red reaction mixture was stirred at 23 °C for 1 h. The reaction was quenched by adding an aqueous ammonia solution [25% (*w*/*w*)] until the reaction solution showed an orange color. The mixture was extracted with dichloromethane (3 x 10 mL), the combined organic layers were washed with saturated NaCl solution and dried over $MgSO_4$. The solvent was evaporated under reduced pressure and the crude product was purified by column chromatography [silica gel; dichloromethane/ammonia in MeOH (7 N)-gradient; then aluminium oxide; *n*-pentane/AcOEt-gradient]. A few drops of HCl solution (1 M in diethyl ether) were added to the product and after evaporation of the solvent, prodiginines (**2**, **13**) were obtained in their hydrochloride form as dark red solids. The products were coated with argon and stored at −20 °C.

(*E*)-6-((*Z*)-2-((4-methoxy-1*H*,1'*H*-[2,2'-bipyrrol]-5-yl)methylene)-5-methyl-2*H*-pyrrol-4-yl)hex-2-en-1-ol hydrochloride (2)

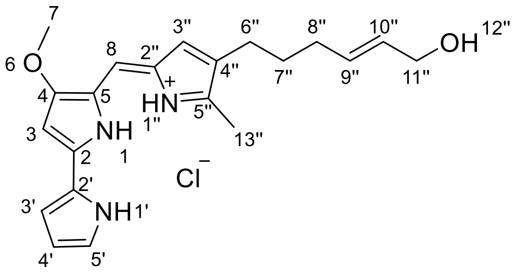

2

Following the general procedure for the synthesis of prodiginines, prodiginine **2** was prepared from *Boc*-**6** (68.0 mg, 0.23 mmol, 1.0 eq.) and the pyrrole **13** (42.0 mg, 0.23 mmol, 1.0 eq.). After purification by column chromatography [silica gel; dichloromethane/ammonia in MeOH (7 N, 0.5-4% gradient); then alumina; *n*-pentane/AcOEt (10-100% gradient; then 1-6% MeOH gradient] and precipitation as hydrochloride, **2** (39.6 mg, 0.10 mmol, 44%) was obtained as dark red solid. **^{1}H-NMR** (600 MHz, $CDCl_3$): δ [ppm] = 1.63 (tt, $^3J_{7'',6''}$ = 7.6 Hz, $^3J_{7'',8''}$ = 7.6 Hz, 2H, 7″-H), 2.08 (dt, $^3J_{8'',7''}$ = 6.9 Hz, $^3J_{8'',9''}$ = 6.9 Hz, 2H, 8″-H), 2.40 (t, $^3J_{6'',7''}$ = 7.6 Hz, 2H, 6″-H), 2.53 (s, 3H, 13″-H), 4.00 (s, 3H, 7-H), 4.10 (d, $^3J_{11'',10''}$ = 5.0 Hz, 2H, 11′-H), 5.67 (ddt, $^3J_{9'',10''}$ = 15.6 Hz, $^3J_{9'',8''}$ = 10.3 Hz, $^3J_{10'',11''}$ = 5.7 Hz, 2H, 9″-H, 10″-H), 6.07 (d, $^4J_{3,1}$ = 1.9 Hz, 1H, 3-H), 6.34 (dd, $^3J_{4',5'}$ = 4.3 Hz, $^4J_{4',1'}$ = 2.3 Hz, 1H, 4′-H), 6.66 (d, $^4J_{3'',1''}$ = 2.7 Hz, 1H, 3″-H), 6.92 (m_c, 1H, 3′-H), 6.93 (s, 1H, 8-H), 7.22 (dd, $^3J_{5',4'}$ = 2.8 Hz, $^3J_{5',1'}$ = 1.4 Hz, 1-H, 5′-H), 12.55 (brs, 1H, 1′-NH), 12.71 (brs, 2H, 1-NH, 1″-NH); **^{13}C-NMR** (151 MHz, $CDCl_3$): δ [ppm] = 12.6 (C-13″), 25.0 (C-6″), 29.6 (C-7″), 31.8 (C-8″), 58.9 (C-7), 63.8 (C-11″), 93.0 (C-3), 111.9 (C-4′), 116.1 (C-3′), 117.4 (C-8), 121.0 (C-5), 122.4 (C-2′), 125.3 (C-2″), 127.2 (C-5′), 127.9 (C-4″), 128.3 (C-3″), 129.7 (C-10″), 132.6 (C-9″), 146.8 (C-5″), 148.1 (C-2), 166.0 (C-4); **IR** (ATR-film): $\tilde{\nu}$ [1/cm] = 3166, 3106, 2932, 2854, 1734, 1630, 1603, 1548, 1509, 1358, 1258, 1140, 1118, 1043, 993, 960, 835, 745; **MS** (APCI, positiv-Ion): *m*/*z* = 352 [$(M)^+$], 334, 163; **HRMS** (ESI, positive ion): calculated for $C_{21}H_{26}N_3O_2$ $(M + H)^+$ = 352.2020, found = 352.2018.

(*Z*)-7-(2-((4-methoxy-1*H*,1'*H*-[2,2'-bipyrrol]-5-yl)methylene)-5-methyl-2*H*-pyrrol-4-yl)heptan-1-ol hydrochloride (13)

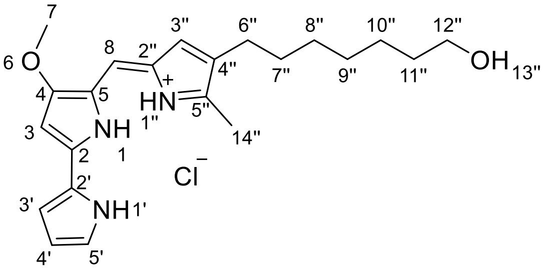

13

Following the general procedure for the synthesis of prodiginines, prodiginine **13** was prepared from *Boc*-**6** (100 mg, 0.34 mmol, 1.0 eq.) and the pyrrole **15** (67 mg, 0.34 mmol, 1.0 eq.). After purification by column chromatography [silica gel; dichloromethane/ammonia in MeOH (7 N, 0.5−4% gradient); then alumina; *n*-pentane/AcOEt (10−100% gradient; then 1−8% MeOH gradient] and precipitation as hydrochloride, **13** (89.8 mg, 0.22 mmol, 65%) was obtained as a dark red solid. **^{1}H-NMR** (600 MHz, $CDCl_3$): δ [ppm] = 1.35 (m_c, 6H, 8″-H, 9″-H, 10″-H), 1.50−1.59 (m, 4H, 7″-H, 11″-H), 2.38 (t, $^3J_{6'',7''}$ = 7.3 Hz, 2H, 6″-H), 2.53 (s, 3H, 14″-H), 3.63 (t, $^3J_{12'',11''}$ = 6.5 Hz, 2H, 12″-H), 3.99 (s, 3H, 7-H), 6.07 (dd, $^4J_{3,1}$ = 3.0 Hz, 1H, 3-H), 6.34 (1H, $^3J_{4',5'}$ = 2.5 Hz, $^4J_{4',1'}$ = 2.5 Hz, 4′-H), 6.66 (s, 1H, 3″-H), 6.91 (s, 1H, 3′-H), 9.94 (s, 1H, 8-H), 7.22 (s, 1H, 5′-H), 12.55 (brs, 1H, 1′-NH), 12.71 (brs, 1H, 1-NH, 1″-NH); **^{13}C-NMR** (151 MHz, $CDCl_3$): δ [ppm] = 12.6 (C-14″), 25.5 (C-6″), 25.8 (C-8″), 29.3 (C-9″), 29.4 (C-10″), 30.2 (C-11″), 32.9 (C-7″), 58.9 (C-7), 63.1 (C-12″), 93.0 (C-3), 111.9 (C-4′), 116.1 (C-8), 117.2 (C-3′), 120.9 (C-5), 122.4 (C-2′), 125.3 (C-2″), 127.0 (C-5′), 127.1 (C-4″), 128.5 (C-3″), 147.0 (C-5″), 147.9 (C-2), 165.9 (C-4); **IR** (ATR-film): $\tilde{\nu}$ [1/cm] = 3007, 2965, 2928, 2855, 1735, 1607, 1540, 1369, 1217, 961; **MS** (APCI, positive ion): *m*/*z* = 368 [$(M)^+$], 341, 313, 267; **HRMS** (ESI, positive ion): calculated for $C_{22}H_{30}N_3O_2$ $(M + H)^+$ = 368.2333, found = 368.2336.

5 Optimization of preparative mutasynthesis

Supplementary Table S2. Optimization of preparative mutasynthesis. The mutasynthesis was performed according to the general procedure for preparative scale mutasynthesis. The final concentration of MAP (**5**) varied from 0.1−1.0 mM resulting in yields between 17% and 62%.

c(MAP) in 500 mL culture [mM]	*n*(MAP) [mmol]	*m*(Prodigiosin) [mg]	*n*(Prodigiosin) [µmol]	yield [%]
1.00	0.50	30.0	83.4	17
0.50	0.25	26.0	72.2	29
0.25	0.125	19.8	55.0	44
0.15	0.075	16.8	46.7	62
0.10	0.05	4.00	11.1	22

6 Calculation of molar extinction coefficient

The determination of the molar extinction coefficients *ε* of the prodiginines **2**, **3**, **12** and **13** was carried out on a UV photometer (*Shimadzu*, Duisburg, Germany). In a triplicate determination, three 4 mM stock solutions were prepared in acidic ethanol [4% (*v*/*v*) 1 N HCl in ethanol] and a 40 µM working solution was prepared in each case by dilution with acidic ethanol. A dilution series was prepared with all three working solutions, resulting in 3 x 6 samples in the concentration range of 1−6 µM for three-fold determination. The volumes used to prepare the dilution series are listed in the following table:

Supplementary Table S3. Dilution series for determination of molar extinction coefficients.

dilution series / µM	added volume of 40 µM stock solution / µL	added volume of acidic ethanol / µL
1	25	975
2	50	950
3	75	925
4	100	900
5	125	875
6	150	850

The absorbances of the dilution series were measured at a wavelength λ of 535 nm. According to *Lambert-Beer's* law, with a linear dependence of absorption *A* and concentration *c*, the molecular extinction coefficient *ε* corresponds to the slope through the layer thickness of the cuvette (polystyrene cuvettes, d = 1 cm):

$$\varepsilon = \frac{A}{c \cdot d} = \frac{\text{slope}}{d} \qquad (1)$$

The respective graphs are shown in **Supplementary Figure S5**.

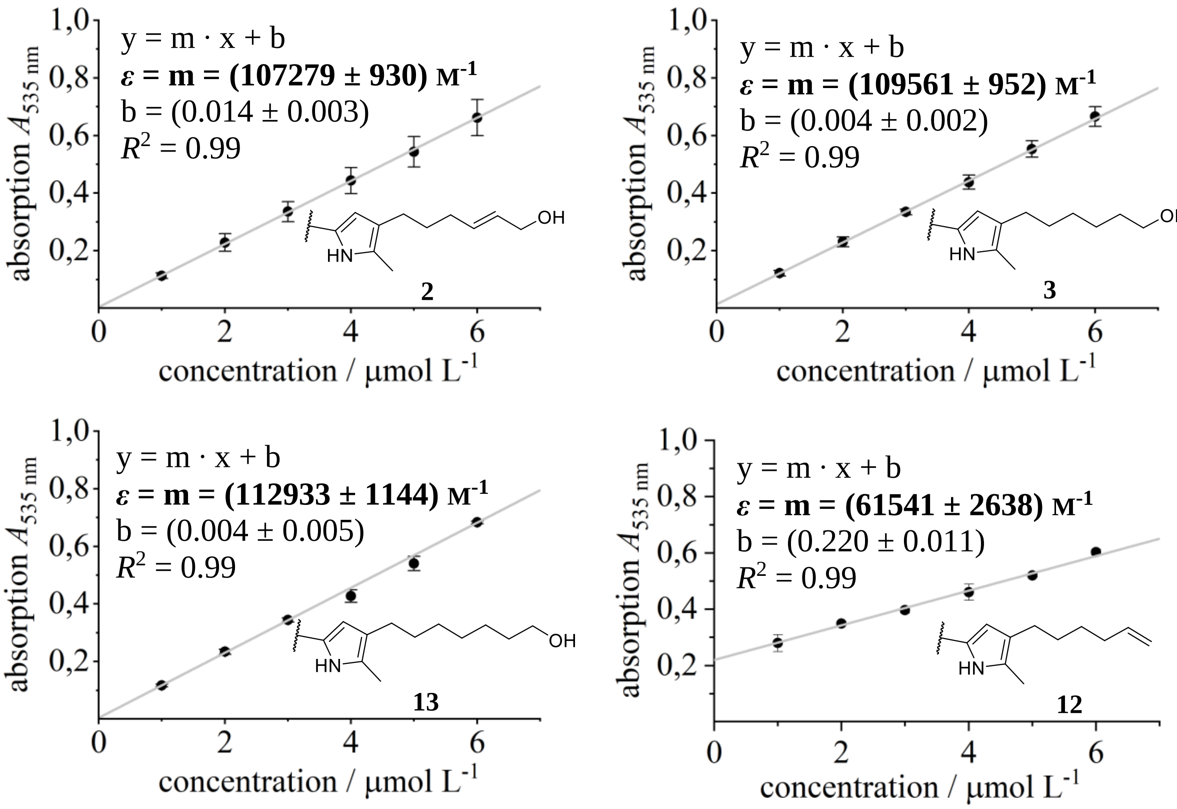

Supplementary Figure S5. Calculation of molar extinction coefficients *ε*. The measurement is carried out in acidic ethanol [4% (*v*/*v*) 1 N HCl] at a wavelength of 535 nm in a three-fold determination. The absorbance values were plotted against the concentrations and linear regression by *OriginPro* 2021 (*OriginLap Corp*.) was used to determine the slopes, which were equated to the molar extinction coefficients *ε*. Chemical structures show the C-pyrrole ring of the measured prodiginine derivatives.

7 Analytical data

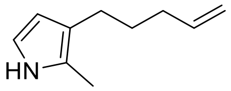

11c

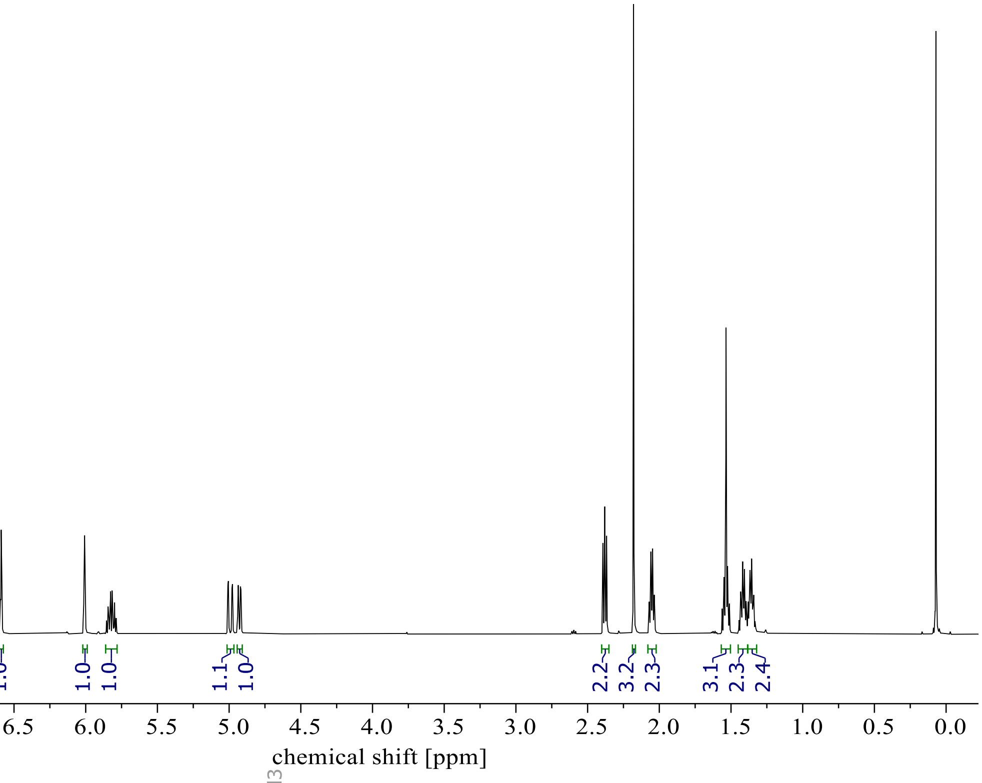

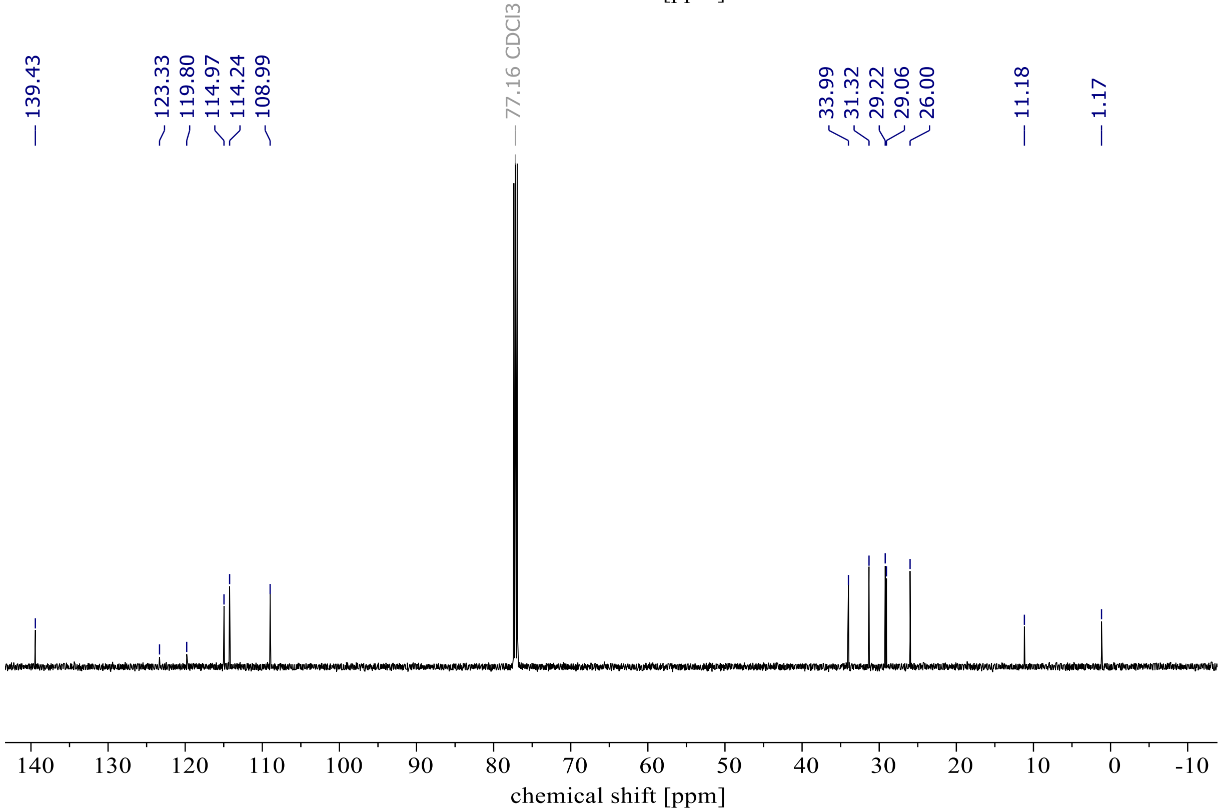

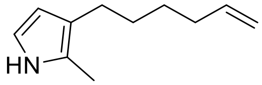
HN

11a

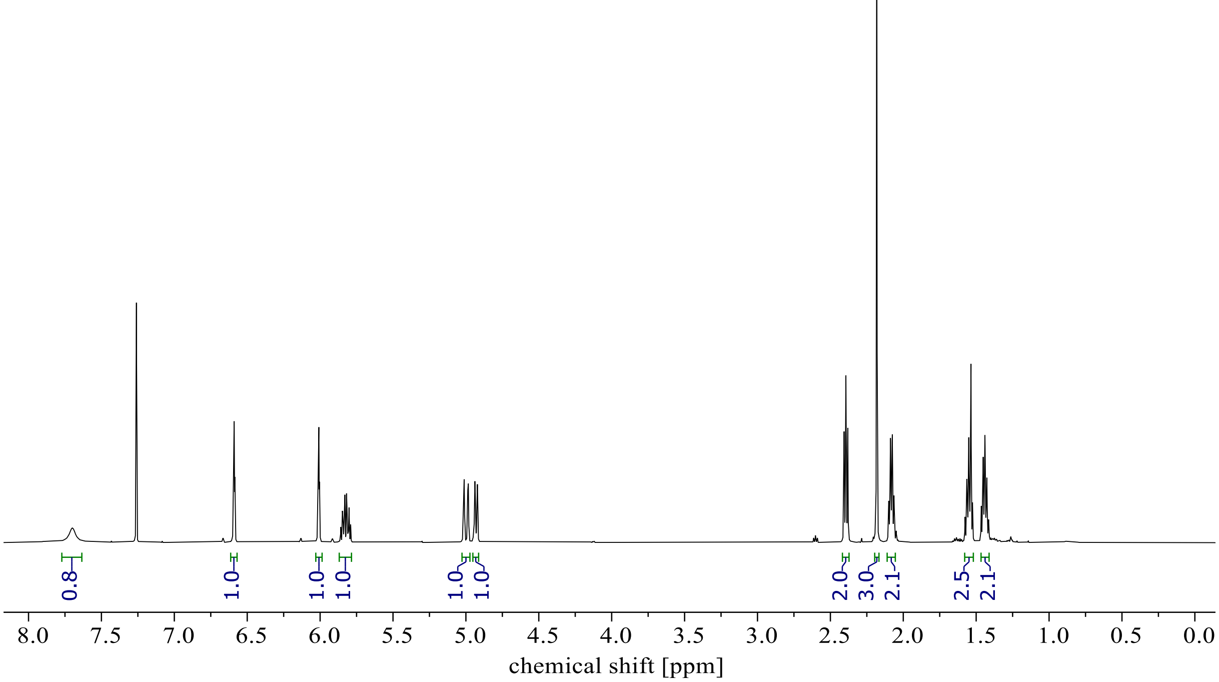
0.8
1.0
1.0
1.0
1.0
1.0
2.0
3.0
2.1
2.5
2.1
8.0 7.5 7.0 6.5 6.0 5.5 5.0 4.5 4.0 3.5 3.0 2.5 2.0 1.5 1.0 0.5 0.0
chemical shift [ppm]

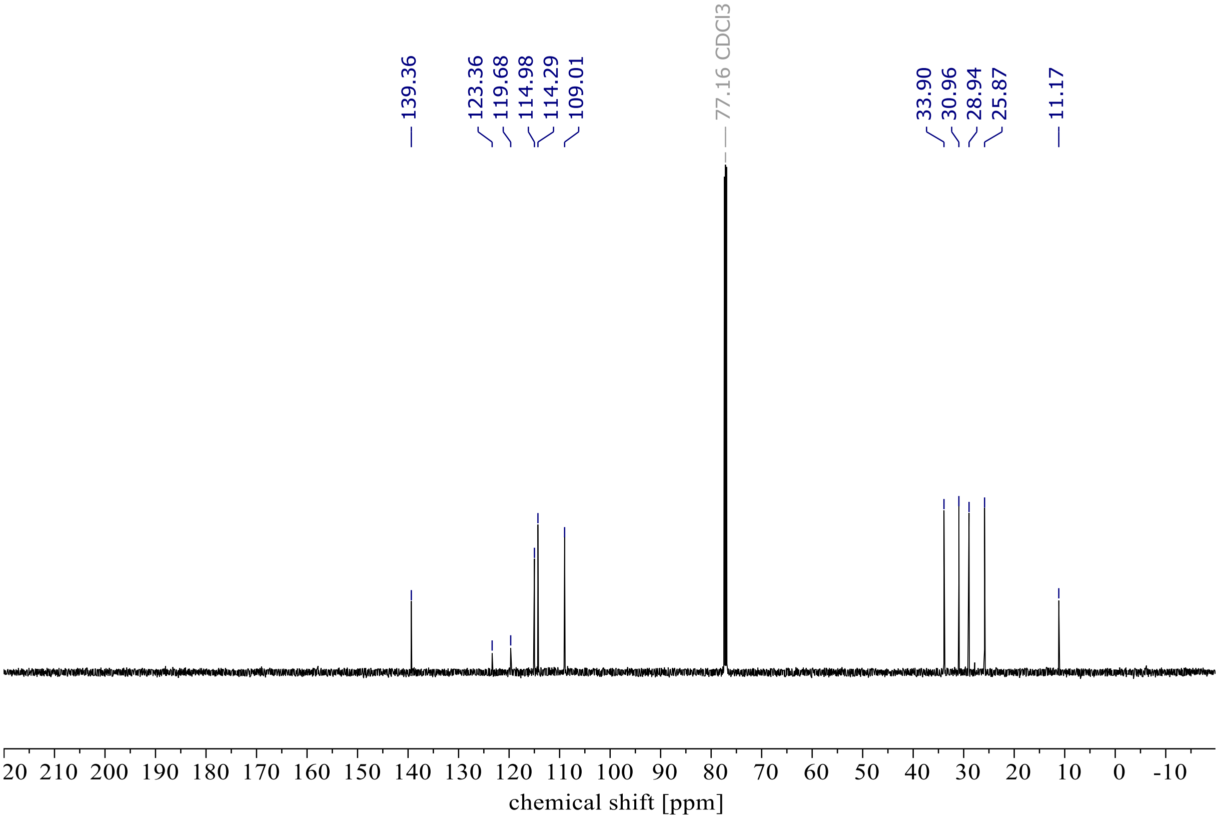
139.36
123.36
119.68
114.98
114.29
109.01
77.16 CDCl3
33.90
30.96
28.94
25.87
11.17
20 210 200 190 180 170 160 150 140 130 120 110 100 90 80 70 60 50 40 30 20 10 0 -10
chemical shift [ppm]

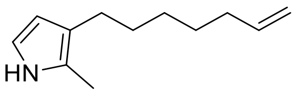
HN

11d

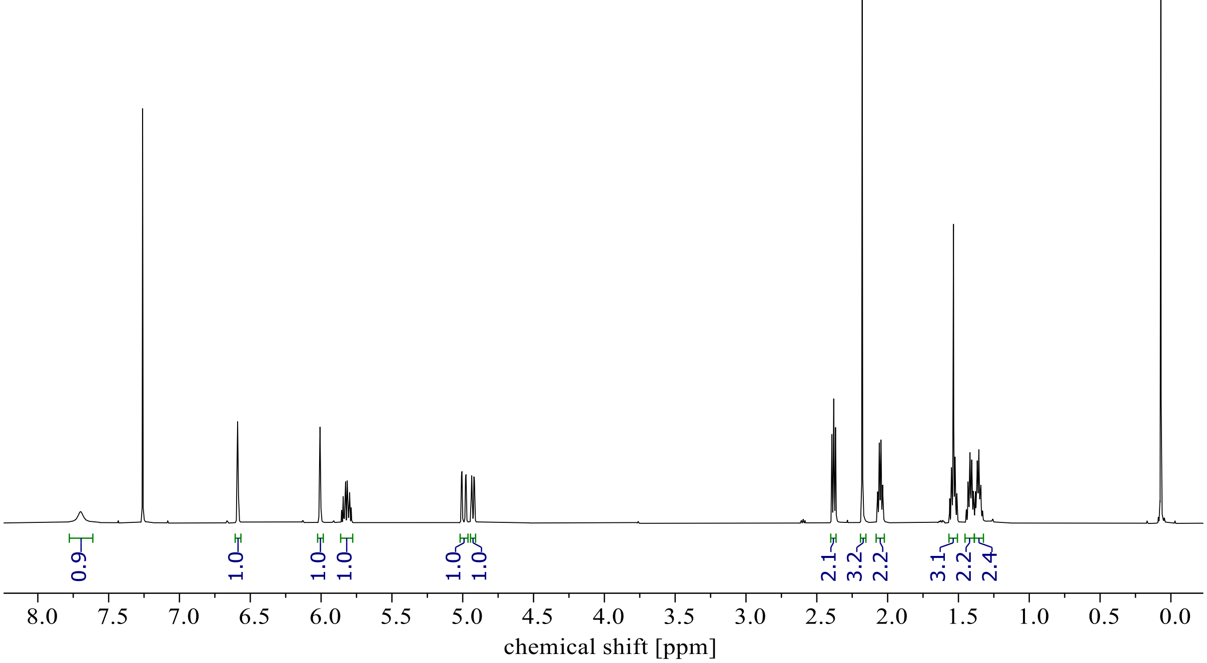
0.9
1.0
1.0
1.0
1.0
1.0
2.1
3.2
2.2
3.1
2.2
2.4
8.0 7.5 7.0 6.5 6.0 5.5 5.0 4.5 4.0 3.5 3.0 2.5 2.0 1.5 1.0 0.5 0.0
chemical shift [ppm]

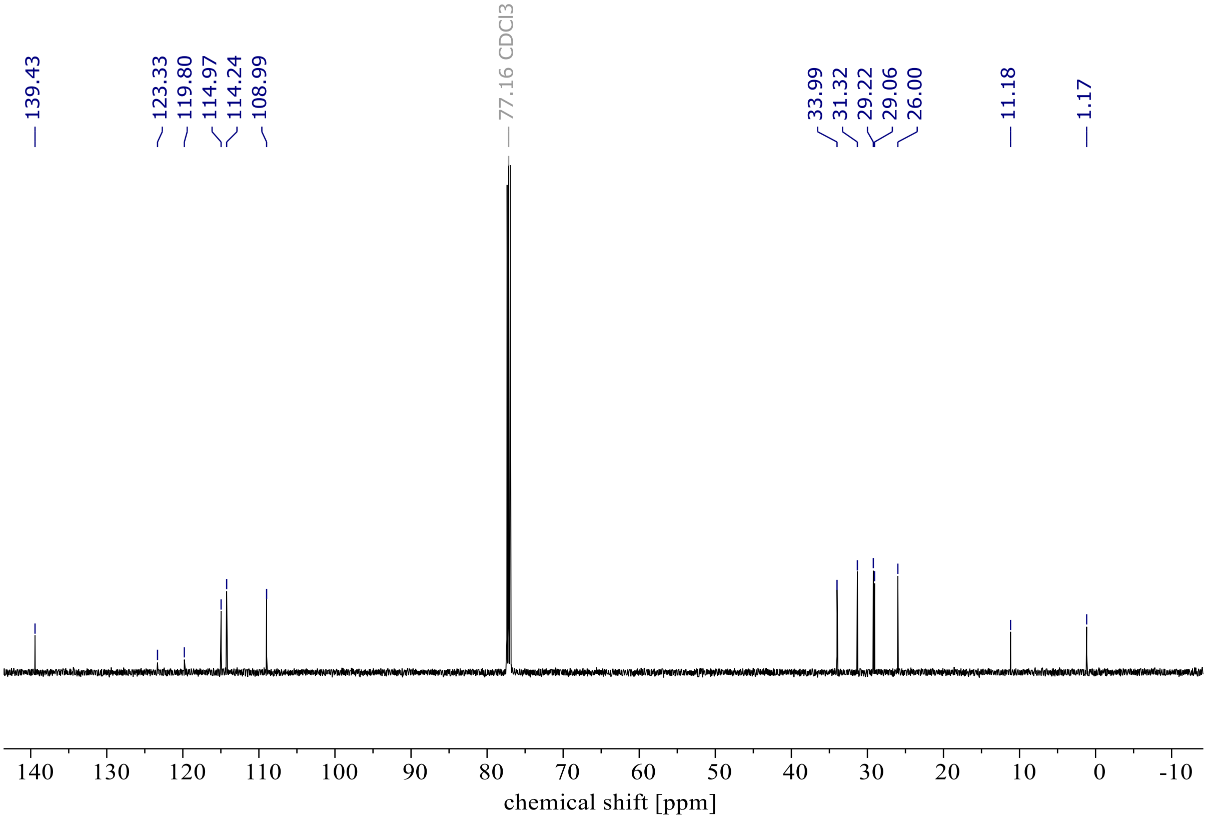
139.43
123.33
119.80
114.97
114.24
108.99
77.16 CDCl3
33.99
31.32
29.22
29.06
26.00
11.18
1.17
140 130 120 110 100 90 80 70 60 50 40 30 20 10 0 -10
chemical shift [ppm]

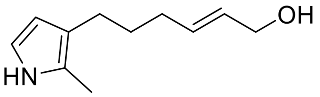
HN
OH

14

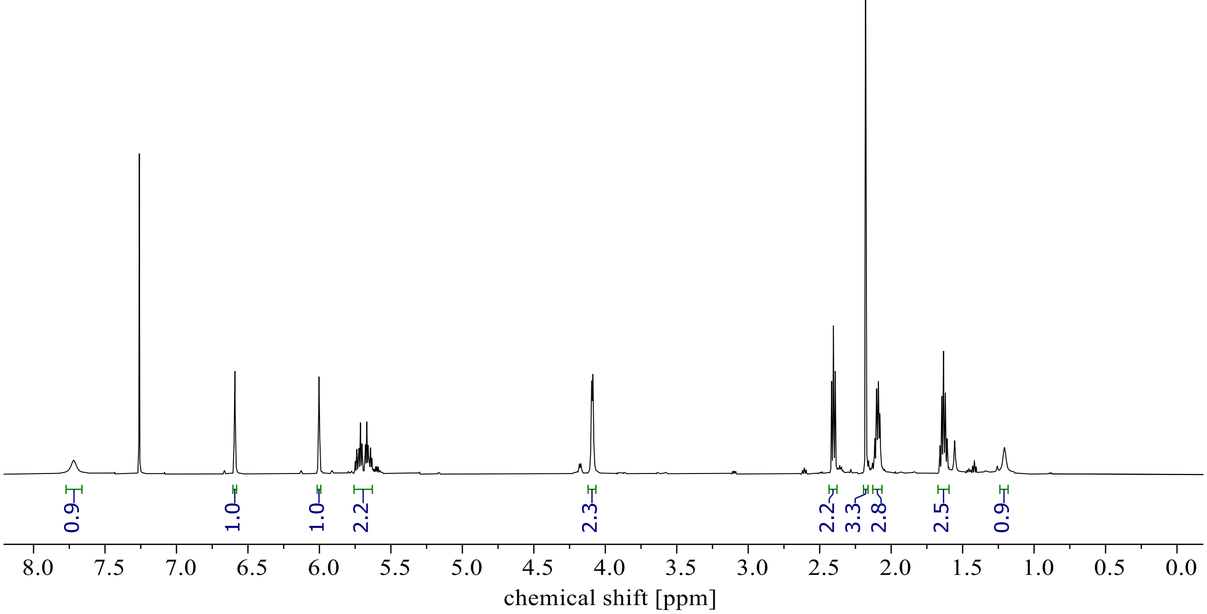
0.9
1.0
1.0
2.2
2.3
2.2
3.3
2.8
2.5
0.9
8.0 7.5 7.0 6.5 6.0 5.5 5.0 4.5 4.0 3.5 3.0 2.5 2.0 1.5 1.0 0.5 0.0
chemical shift [ppm]

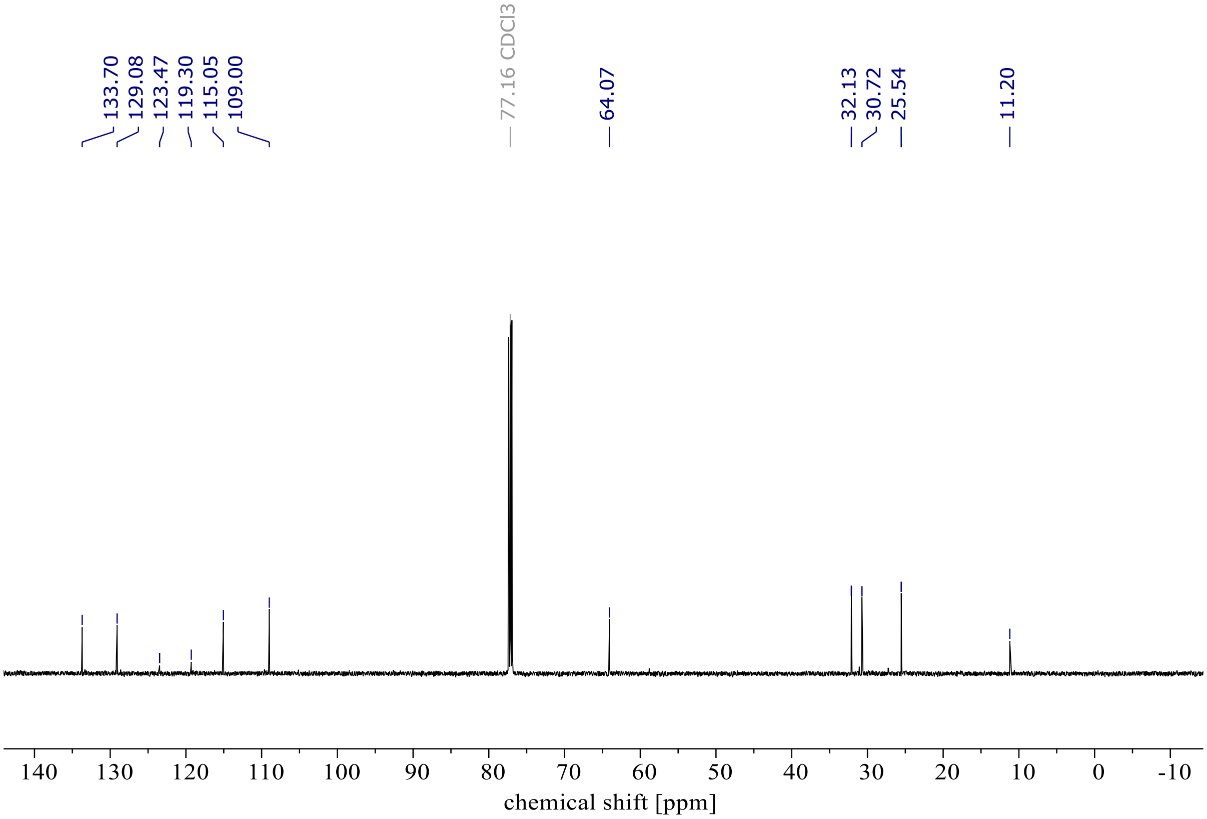
133.70
129.08
123.47
119.30
115.05
109.00
77.16 CDCl3
64.07
32.13
30.72
25.54
11.20
140 130 120 110 100 90 80 70 60 50 40 30 20 10 0 -10
chemical shift [ppm]

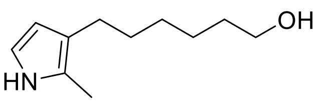
OH
HN

11b

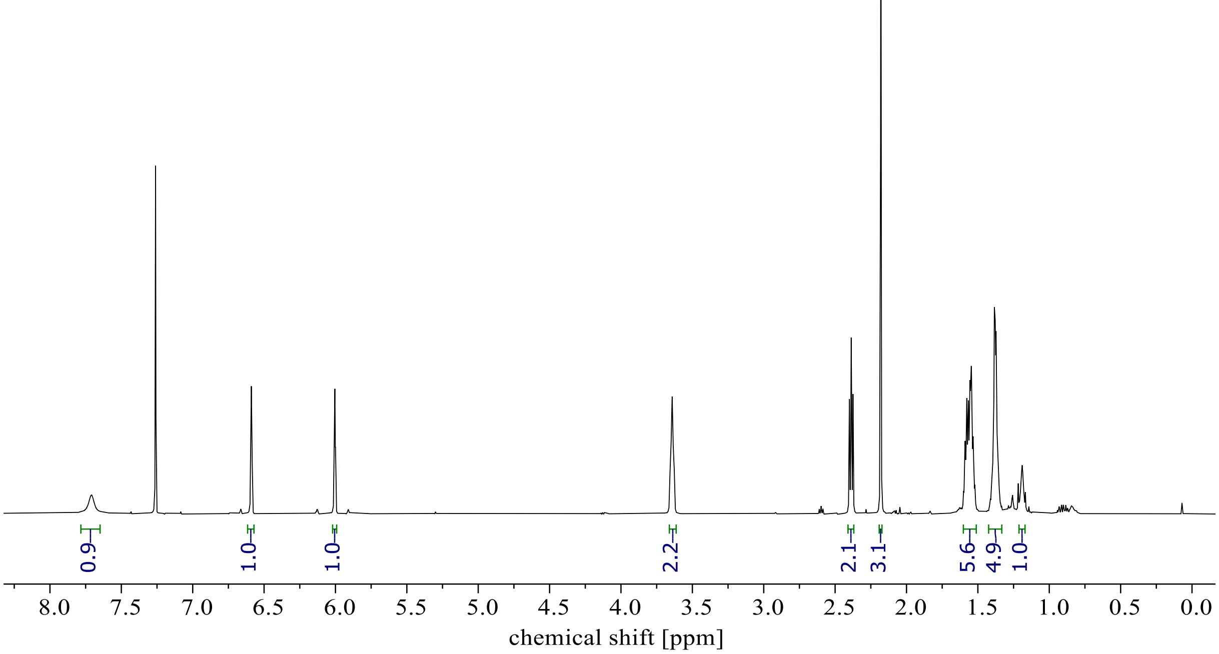
0.9
1.0
1.0
2.2
2.1
3.1
5.6
4.9
1.0
8.0 7.5 7.0 6.5 6.0 5.5 5.0 4.5 4.0 3.5 3.0 2.5 2.0 1.5 1.0 0.5 0.0
chemical shift [ppm]

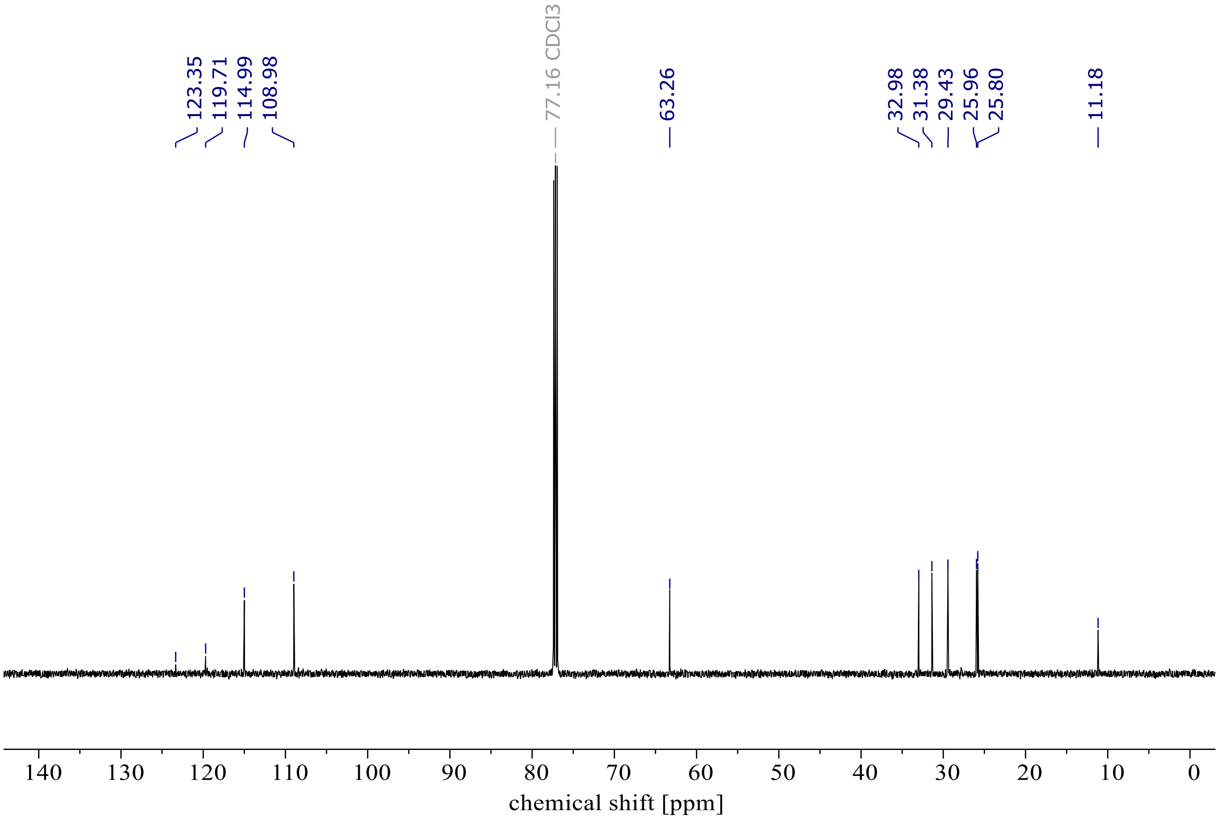
123.35
119.71
114.99
108.98
77.16 CDCl3
63.26
32.98
31.38
29.43
25.96
25.80
11.18
140 130 120 110 100 90 80 70 60 50 40 30 20 10 0
chemical shift [ppm]

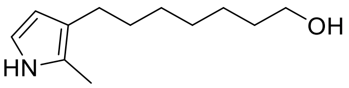
HN
OH

15

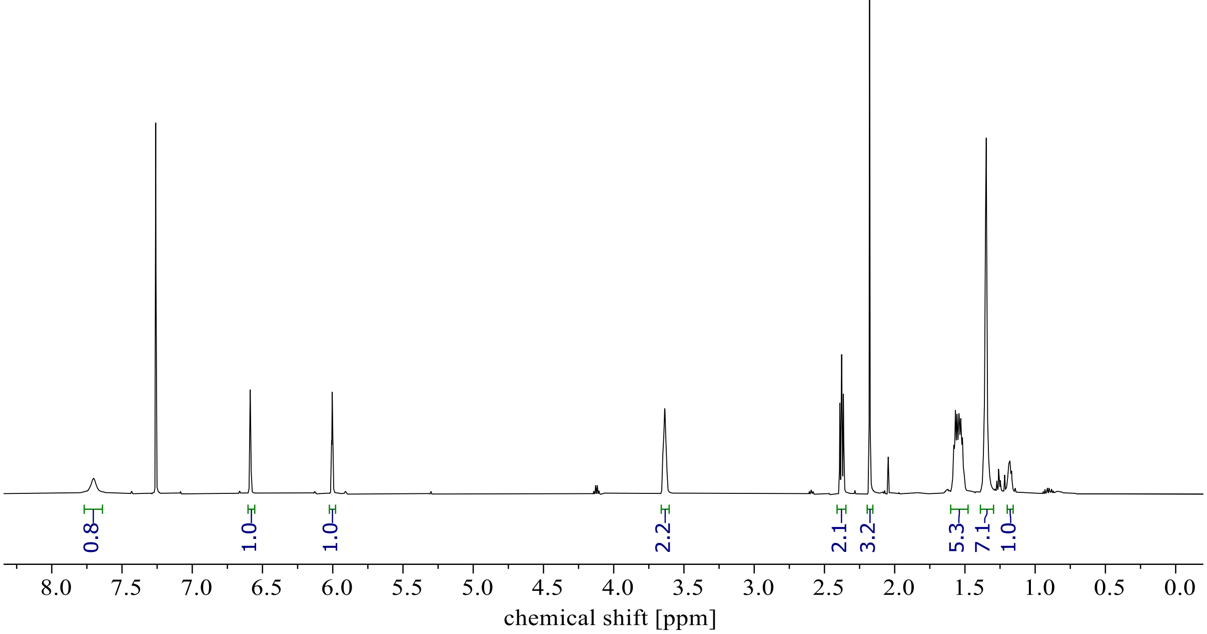
0.8
1.0
1.0
2.2
2.1
3.2
5.3
7.1
1.0
8.0 7.5 7.0 6.5 6.0 5.5 5.0 4.5 4.0 3.5 3.0 2.5 2.0 1.5 1.0 0.5 0.0
chemical shift [ppm]

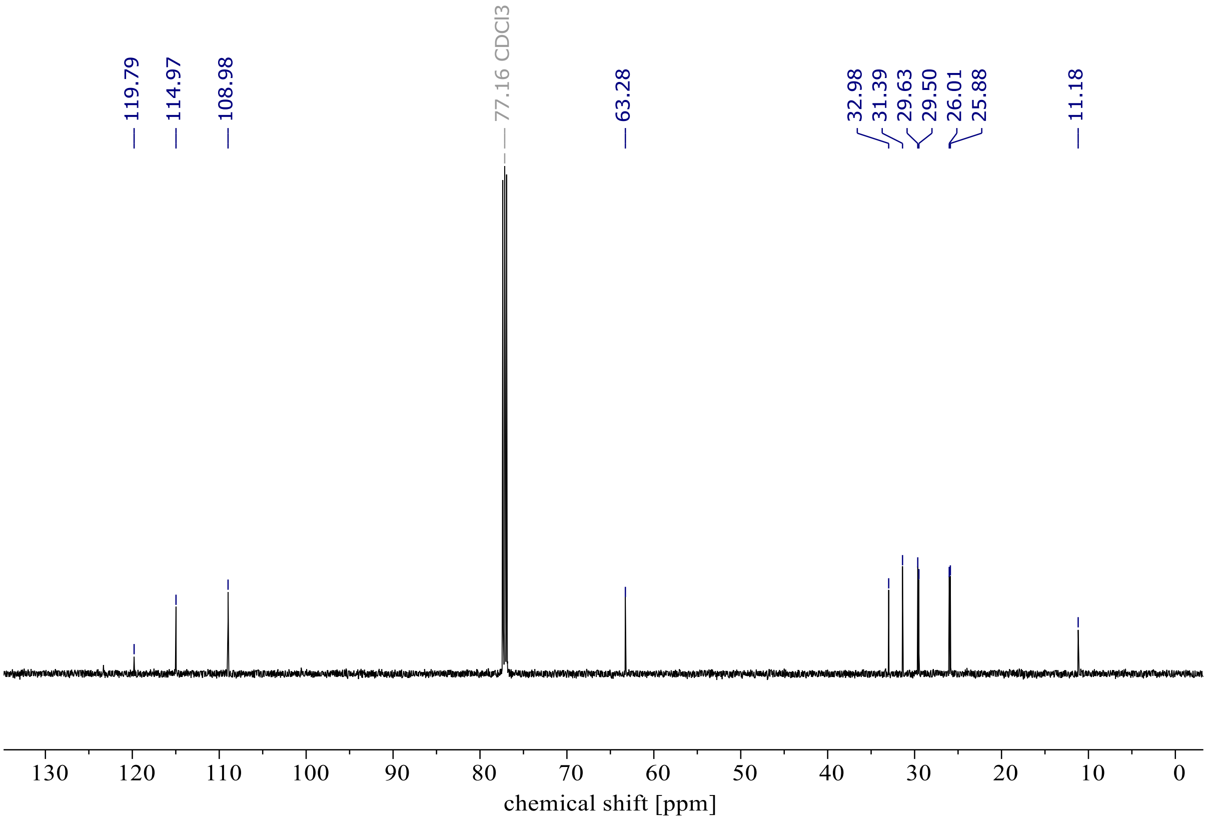
119.79
114.97
108.98
77.16 CDCl3
63.28
32.98
31.39
29.63
29.50
26.01
25.88
11.18
130 120 110 100 90 80 70 60 50 40 30 20 10 0
chemical shift [ppm]

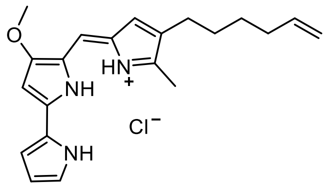
O
HN
+
NH
Cl−
NH

12

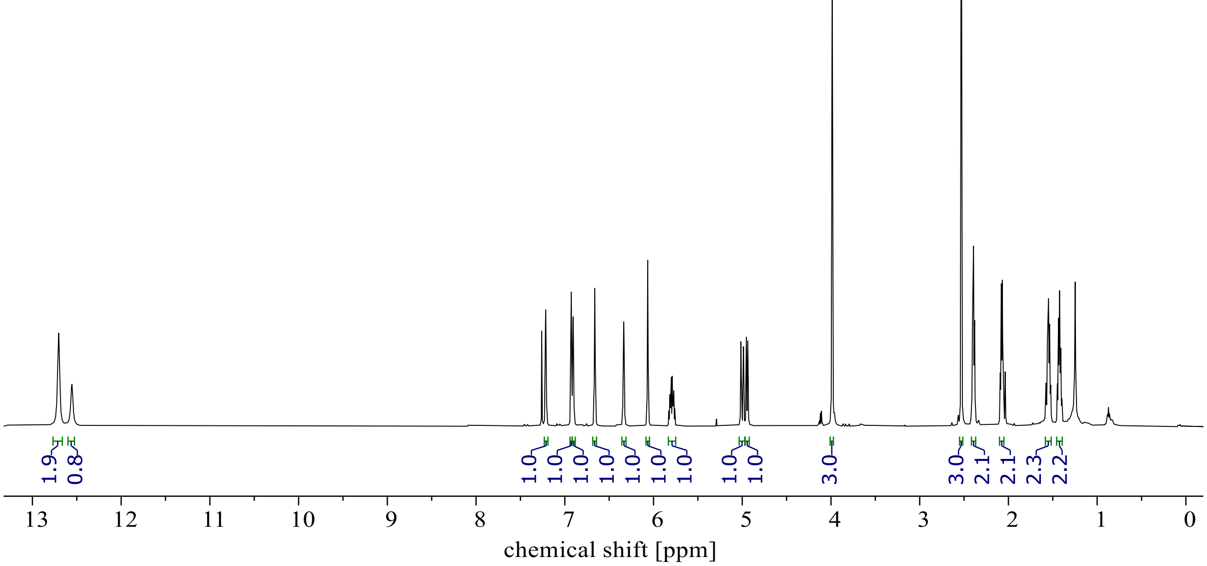
1.9
0.8
1.0
1.0
1.0
1.0
1.0
1.0
1.0
1.0
1.0
3.0
3.0
2.1
2.1
2.3
2.2
13 12 11 10 9 8 7 6 5 4 3 2 1 0
chemical shift [ppm]

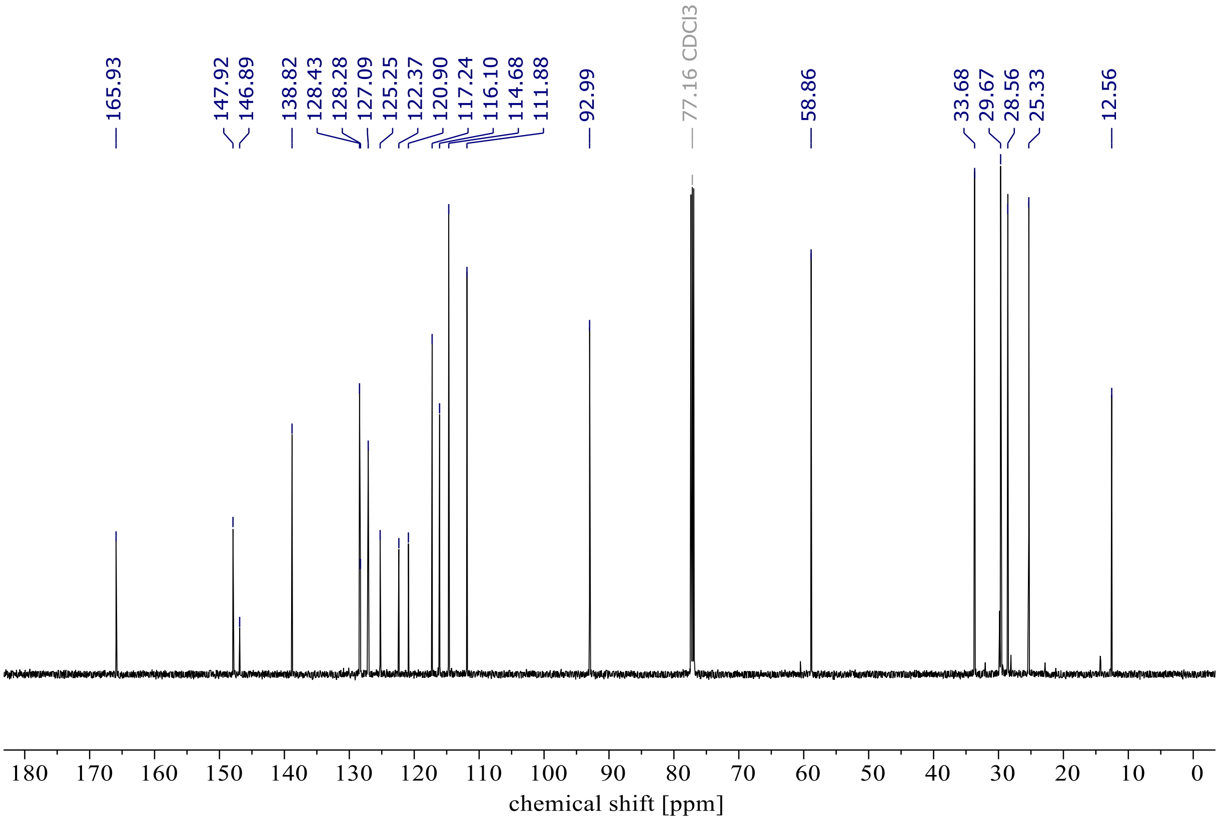
165.93
147.92
146.89
138.82
128.43
128.28
127.09
125.25
122.37
120.90
117.24
116.10
114.68
111.88
92.99
77.16 CDCl3
58.86
33.68
29.67
28.56
25.33
12.56
180 170 160 150 140 130 120 110 100 90 80 70 60 50 40 30 20 10 0
chemical shift [ppm]

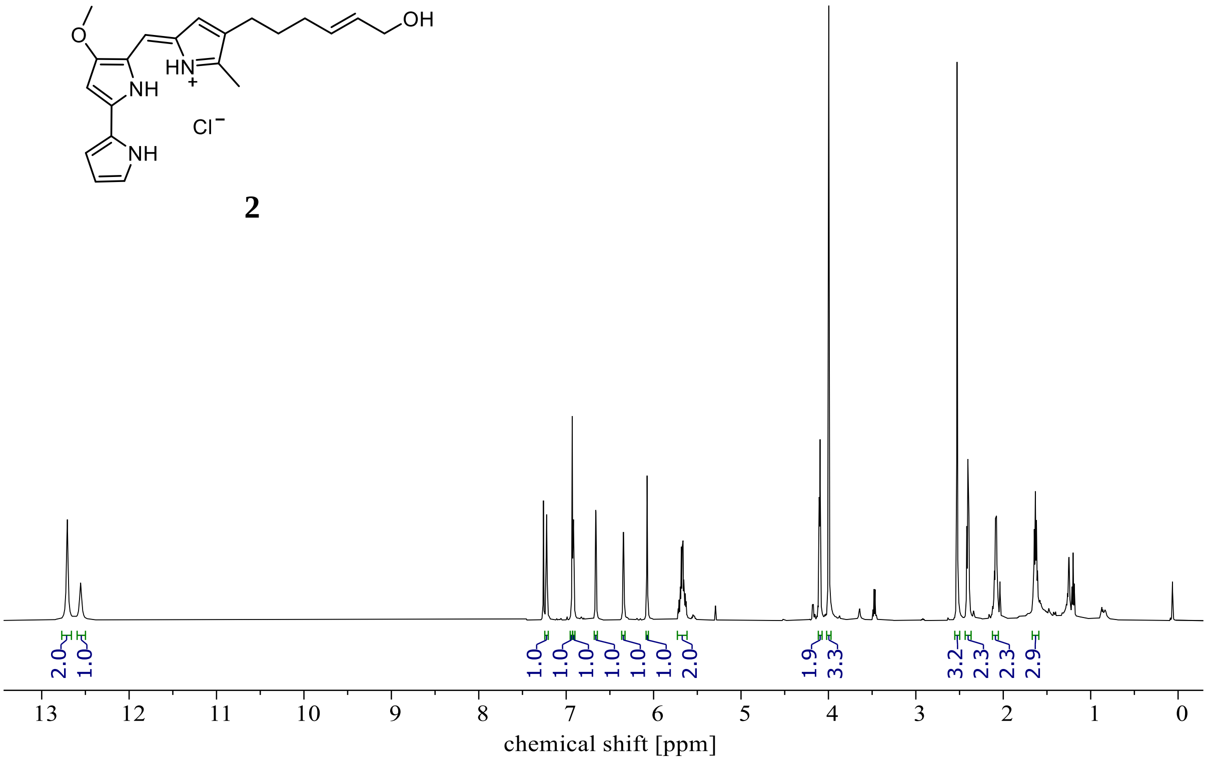
O
NH
HN
+
Cl−
NH
OH
2
2.0
1.0
1.0
1.0
1.0
1.0
1.0
1.0
2.0
1.9
3.3
3.2
2.3
2.3
2.9
13 12 11 10 9 8 7 6 5 4 3 2 1 0
chemical shift [ppm]

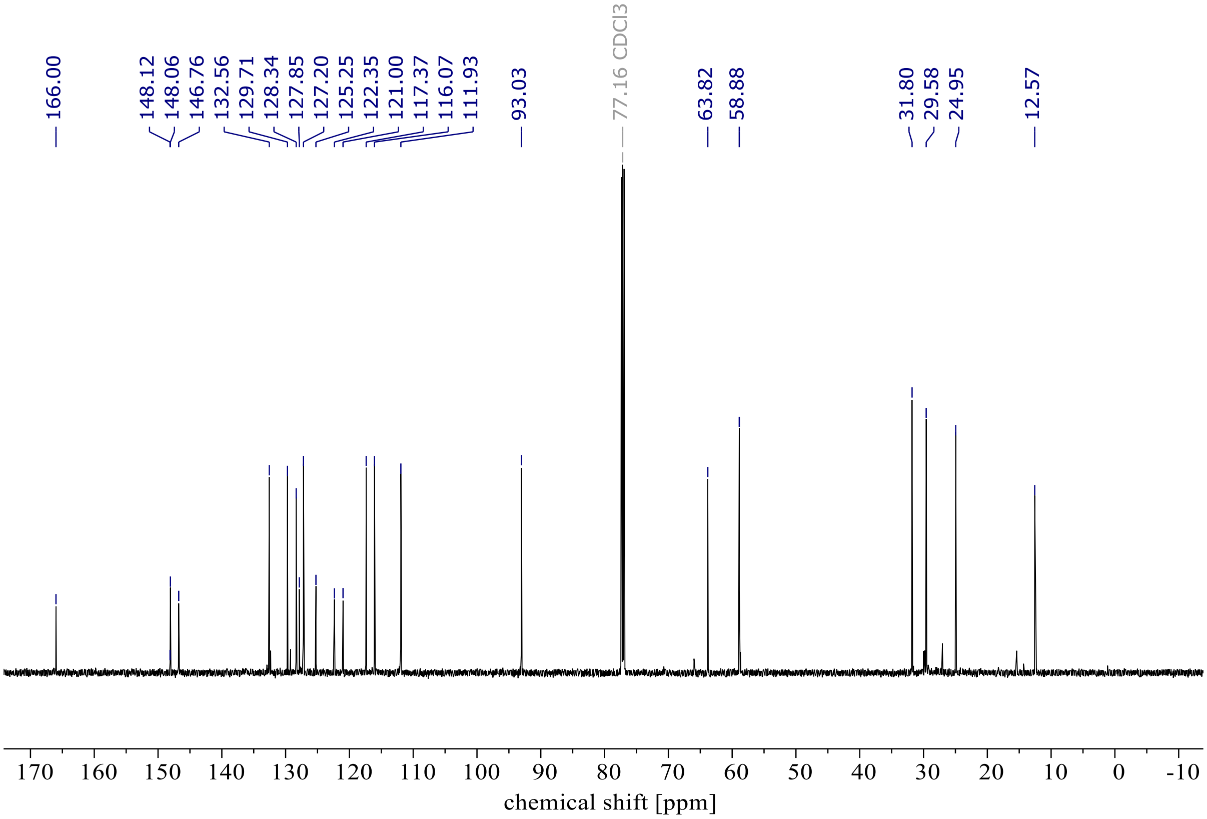
166.00
148.12
148.06
146.76
132.56
129.71
128.34
127.85
127.20
125.25
122.35
121.00
117.37
116.07
111.93
93.03
77.16 CDCl3
63.82
58.88
31.80
29.58
24.95
12.57
170 160 150 140 130 120 110 100 90 80 70 60 50 40 30 20 10 0 -10
chemical shift [ppm]

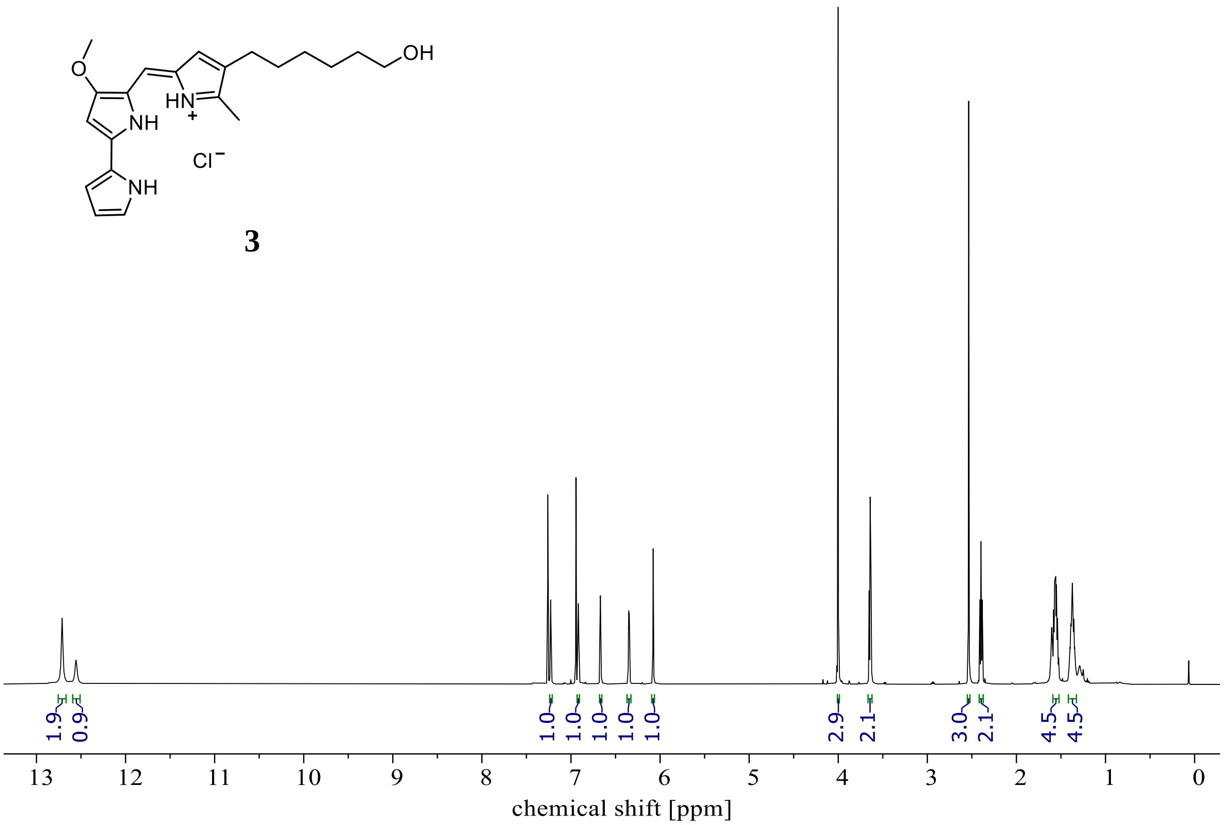
OH
O
NH
HN
+
Cl−
NH
3
1.9
0.9
1.0
1.0
1.0
1.0
1.0
2.9
2.1
3.0
2.1
4.5
4.5
13 12 11 10 9 8 7 6 5 4 3 2 1 0
chemical shift [ppm]

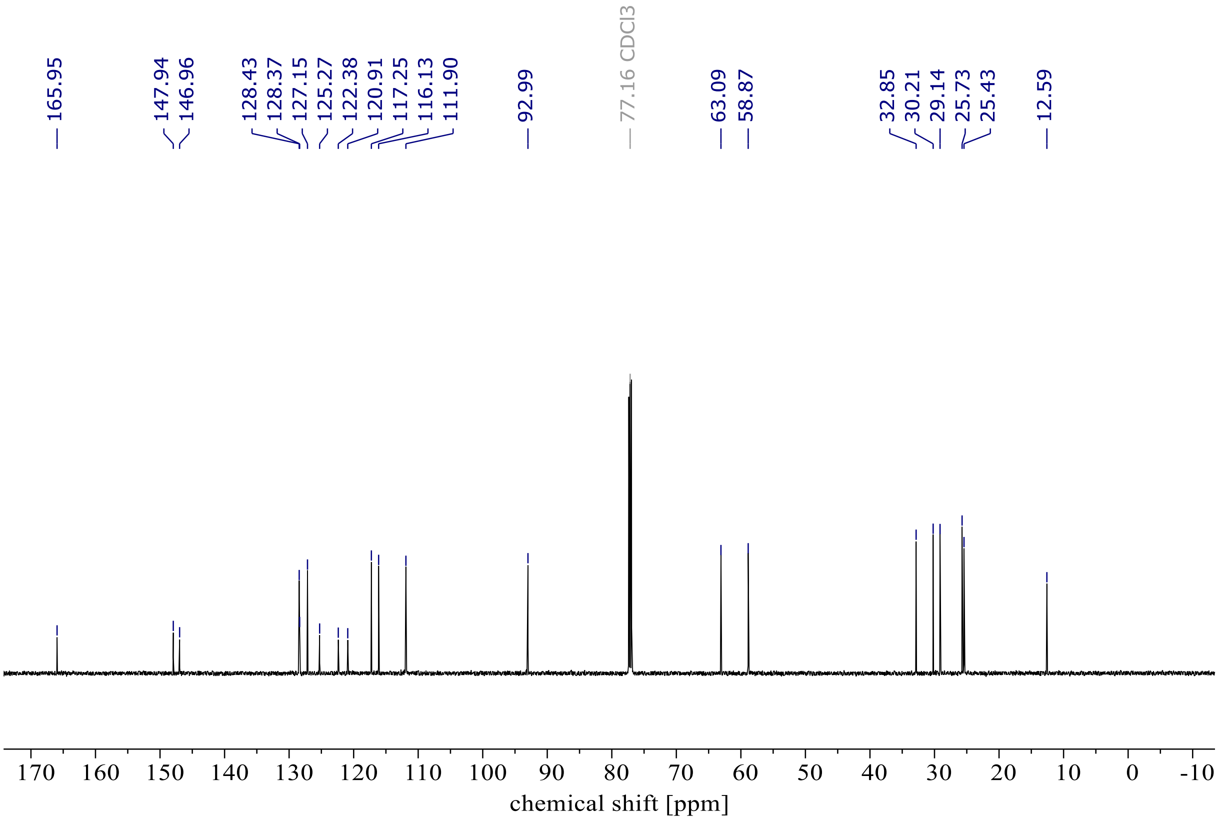
165.95
147.94
146.96
128.43
128.37
127.15
125.27
122.38
120.91
117.25
116.13
111.90
92.99
77.16 CDCl3
63.09
58.87
32.85
30.21
29.14
25.73
25.43
12.59
170 160 150 140 130 120 110 100 90 80 70 60 50 40 30 20 10 0 -10
chemical shift [ppm]

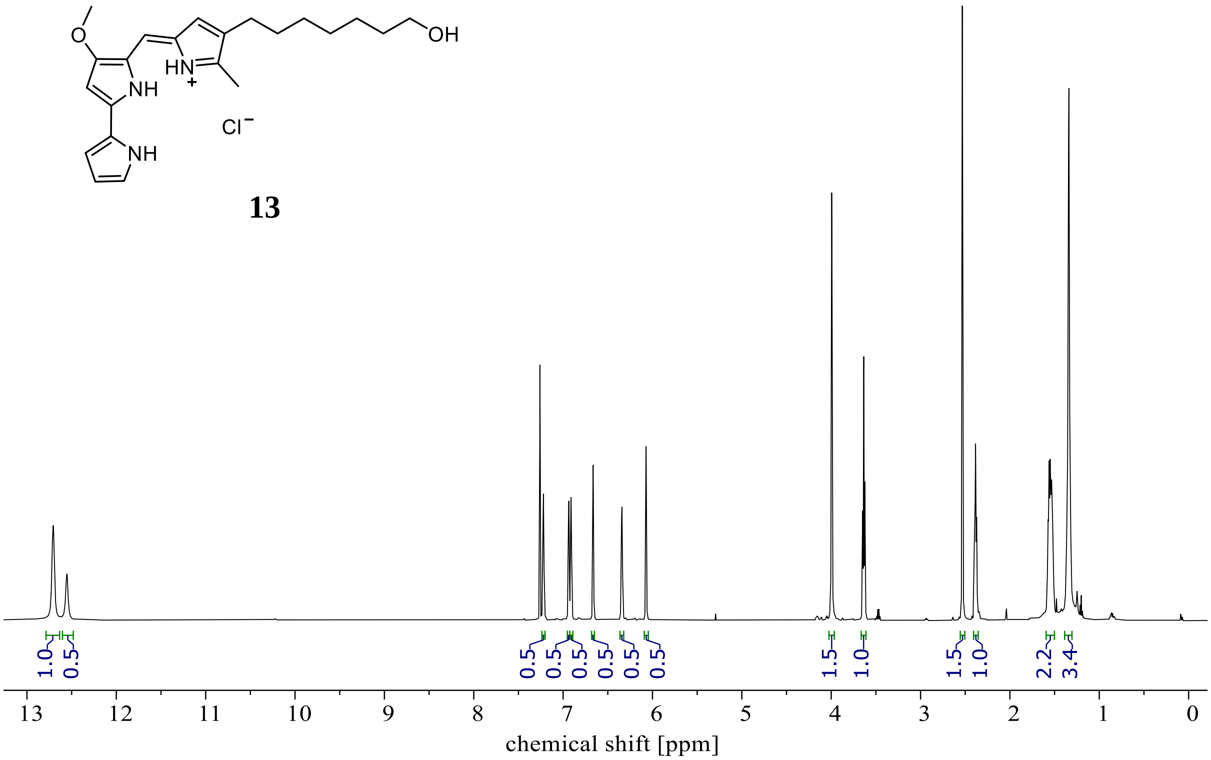
O
NH
HN
+
OH
Cl⁻
NH
13
1.0
0.5
0.5
0.5
0.5
0.5
0.5
0.5
1.5
1.0
1.5
1.0
2.2
3.4
13 12 11 10 9 8 7 6 5 4 3 2 1 0
chemical shift [ppm]

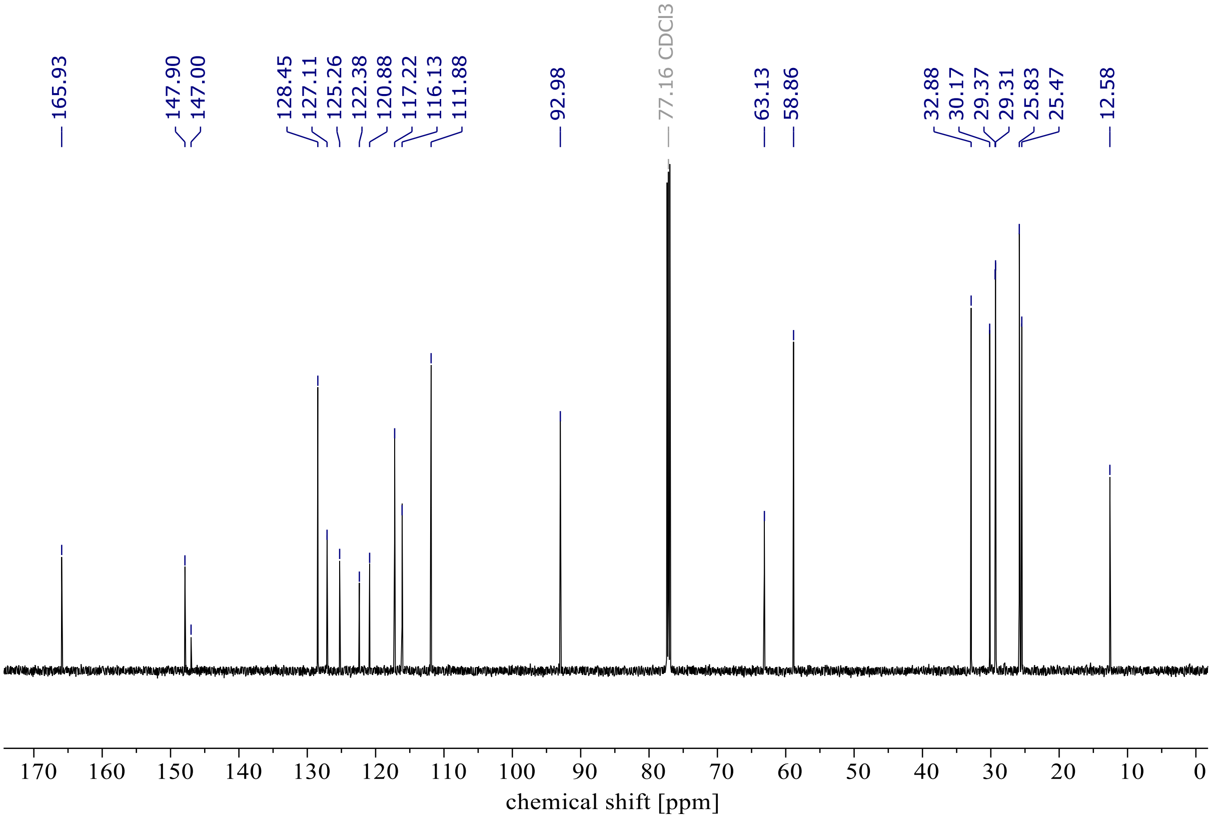
165.93
147.90
147.00
128.45
127.11
125.26
122.38
120.88
117.22
116.13
111.88
92.98
77.16 CDCl3
63.13
58.86
32.88
30.17
29.37
29.31
25.83
25.47
12.58
170 160 150 140 130 120 110 100 90 80 70 60 50 40 30 20 10 0
chemical shift [ppm]

III Impact of prodiginines on the plant parasitic nematode *Heterodera schachtii*

Supplementary Table S4. List of concentrations used for treatments.

Treatment	Concentration (µg/mL)					
Di-rhamnolipid (**4**)	1.125	2.500	5.000	7.500	10.000	-
Prodigiosin (**1**)	1.260	2.519	5.039	7.558	10.077	-
Hydroxylated prodiginine **3**	1.365	2.729	5.459	8.188	10.918	13.647
Hydroxylated prodiginine **2**	1.358	2.715	5.431	8.146	10.861	13.577
Hydroxylated prodiginine **13**	1.414	2.828	5.655	8.483	11.311	14.138

All prodiginines were obtaines as hydrochlorides. Accordingly, all given weights and concentrations refer to the respective molecular weights.

Supplementary Table S5. List of treatments and concentrations used in the combinatorial assay.

Treatment	Concentration (µg/mL)		
	Di-rhamnolipid (**4**)	Prodigiosin (**1**)	Hydroxylated prodiginine **3**
Compound alone (EC_{50})	2.77	-	-
	-	5.43	-
	-	-	12.16
Compound combination (0.25x, 0.5x, 0.75x, 1x, 1.25x of EC_{50})	0.69	1.36	-
	1.39	2.72	-
	2.08	4.07	-
	2.77	5.43	-
	3.46	6.79	-
	0.69	-	3.04
	1.39	-	6.08
	2.08	-	9.12
	2.77	-	12.16
	3.46	-	15.20

All prodiginines were obtaines as hydrochlorides. Accordingly, all given weights and concentrations refer to the respective molecular weights.

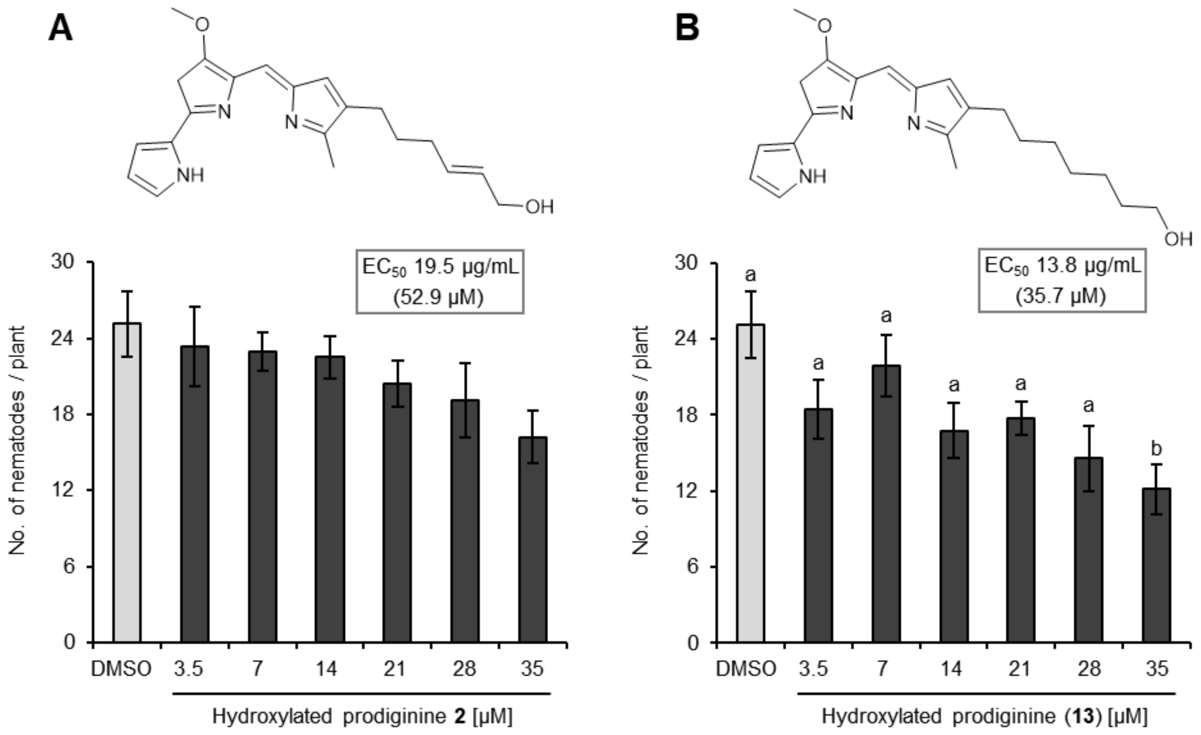

Supplementary Figure S6. EC_{50} determination of hydroxylated prodiginines 2 and 13 on the plant-parasitic nematode *Heterodera schachtii*. Effects of hydroxylated prodinines **2 (A)** and **13 (B)** were assessed as reduction of nematode numbers on *A. thaliana*. EC_{50} values (effective concentration that causes a reduction of nematode infestation of *A. thaliana* by 50%) were calculated by using software 'CompuSyn'. Results are expressed as the mean ± standard error of two independent biological replicates (n≥16; at least 8 plants/treatment were evaluated per biological replicate). Different letters indicate statistically significant differences among treatments according to Dunn's Method (p<0.05). No significant differences were found for treatments with different concentrations of prodiginine **2**.

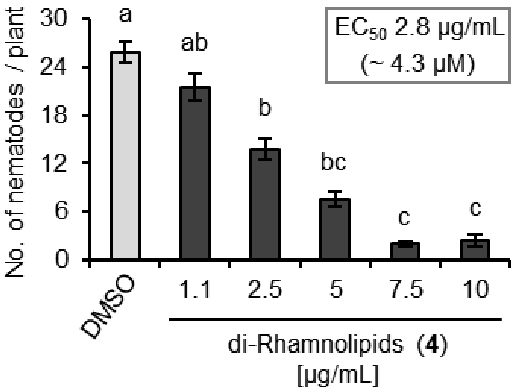

Supplementary Figure S7. EC_{50} determination of di-rhamnolipids (4) on the plant-parasitic nematode *Heterodera schachtii*. The effect of di-rhamnolipids (**4**) was assessed as reduction of nematode numbers on *A. thaliana*. The EC_{50} value (effective concentration that causes a reduction of nematode infestation of *A. thaliana* by 50%) was calculated by using software 'CompuSyn'. Results are expressed as the mean ± standard error of three independent biological replicates (n≥20). Different letters indicate statistically significant differences among treatments according to Dunn's Method (p<0.05).

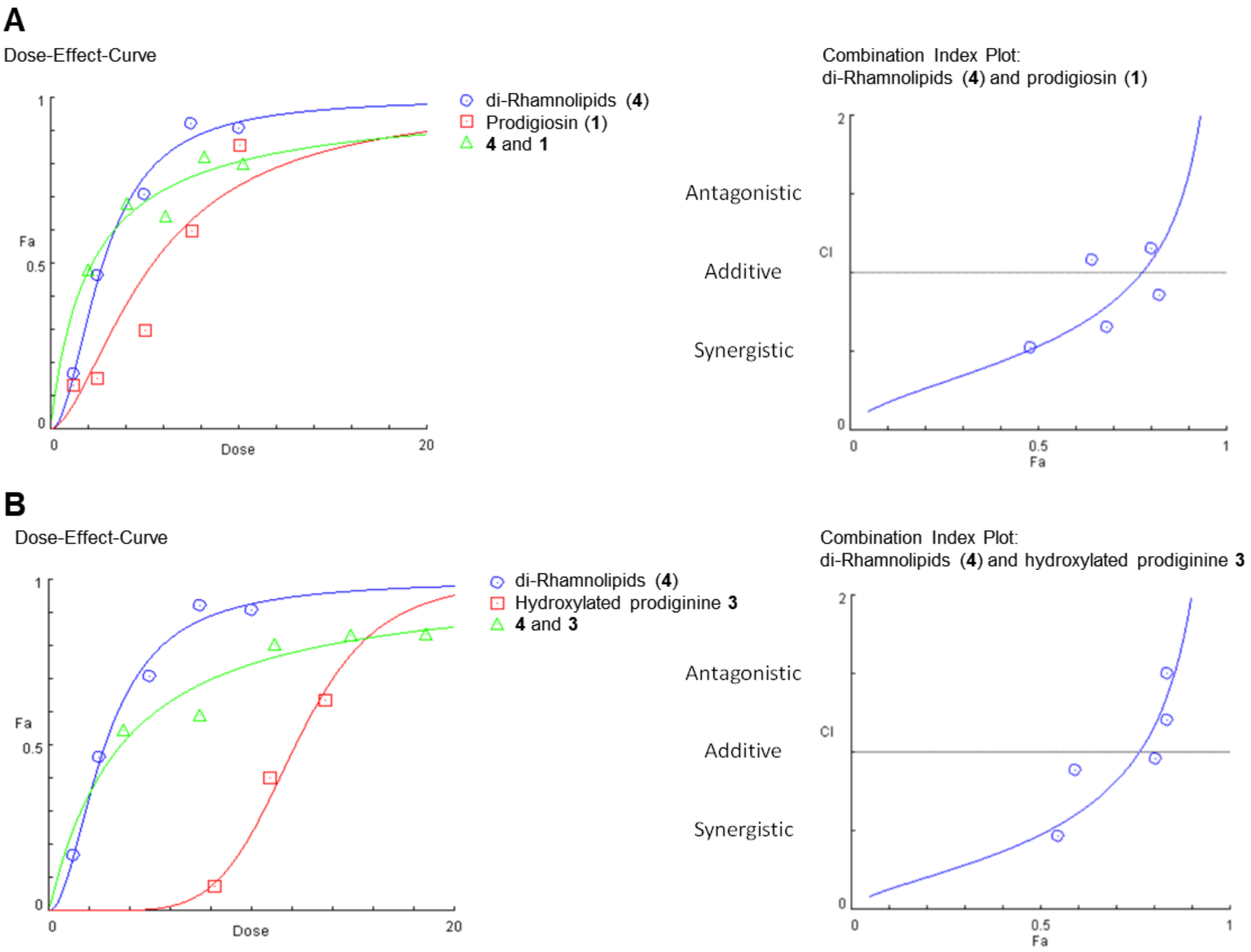

Supplementary Figure S8. Dose-effect curves and combination index plots of prodiginines and di-rhamnolipids. Effects of the compound combinations were assessed as reduction of *Heterodera schachtii* numbers on *Arabidopsis thaliana*. The schematic representation was generated by software 'CompuSyn' (Chou and Martin, 2005) based on five concentrations (0.25x, 0.5x, 0.75x, 1x, 1.25x of before determined EC_{50} (see Tables S4 and S5)) of prodigiosin (**1**) **(A)** or hydroxylated prodiginine **3** **(B)** applied alone or in combination with di-rhamnolipids (**4**). Dose – total compound dose in µg/mL; Fa – fraction affected; CI – combination index (determined using 'CompuSyn'(Chou and Martin, 2005)).

Supplementary References

Aldrich, L. N. (2012). Progress toward the total synthesis of marineosins A & B; total synthesis of tambjamine K and unnatural analogs with improved anticancer activity, and discovery of selective M1 antagonists.

Brands, S., Brass, H. U. C., Klein, A. S., Pietruszka, J., Ruff, A. J., and Schwaneberg, U. (2020). A colourimetric high-throughput screening system for directed evolution of prodigiosin ligase PigC. *Chem. Commun.* 56, 8631–8634. doi:10.1039/d0cc02181d.

Brass, H. U. C., Klein, A. S., Nyholt, S., Classen, T., and Pietruszka, J. (2019). Condensing enzymes from *Pseudoalteromonadaceae* for prodiginine synthesis. *Adv. Synth. Catal.* 361, 2659–2667.

Chawrai, S. R., Williamson, N. R., Mahendiran, T., Salmond, G. P. C., and Leeper, F. J. (2012). Characterisation of PigC and HapC, the prodigiosin synthetases from *Serratia* sp. and *Hahella chejuensis* with potential for biocatalytic production of anticancer agents. *Chem. Sci.* 3, 447–454. doi:10.1039/c1sc00588j.

Chou, T., and Martin, N. (2005). CompuSyn for drug combinations: PC software and user's guide: A computer program for quantitation of synergism and antagonism in drug combinations, and the determination of IC50 and ED50 and LD50 values. *ComboSyn Inc, Paramus,*.

Couturier, M., Bhalara, H. D., Chawrai, S. R., Monson, R., Williamson, N. R., Salmond, G. P. C., et al. (2019). Substrate flexibility of the flavin-dependent dihydropyrrole oxidases PigB and HapB involved in antibiotic prodigiosin biosynthesis. *ChemBioChem* 21, 523–530. doi:10.1002/cbic.201900424.

de Lorenzo, V., Eltis, L., Kessler, B., and Timmis, K. N. (1993). Analysis of *Pseudomonas* gene products using *lacI*q/*Ptrp-lac* plasmids and transposons that confer conditional phenotypes. *Gene* 123, 17–24. doi:10.1016/0378-1119(93)90533-9.

Domröse, A., Weihmann, R., Thies, S., Jaeger, K. E., Drepper, T., and Loeschcke, A. (2017). Rapid generation of recombinant *Pseudomonas putida* secondary metabolite producers using yTREX. *Synth. Syst. Biotechnol.* 2, 310–319. doi:10.1016/j.synbio.2017.11.001.

Habash, S. S., Brass, H. U. C., Klein, A. S., Klebl, D. P., Weber, T. M., Classen, T., et al. (2020). Novel prodiginine derivatives demonstrate bioactivities on plants, nematodes, and fungi. *Front. Plant Sci.* 11, 579807. doi:10.3389/fpls.2020.579807.

Klein, A. S., Domröse, A., Bongen, P., Brass, H. U. C., Classen, T., Loeschcke, A., et al. (2017). New Prodigiosin Derivatives Obtained by Mutasynthesis in *Pseudomonas putida*. *ACS Synth. Biol.* 6, 1757–1765. doi:10.1021/acssynbio.7b00099.

Loeschcke, A., Markert, A., Wilhelm, S., Wirtz, A., Rosenau, F., Jaeger, K.-E., et al. (2013). TREX: A universal tool for the transfer and expression of biosynthetic pathways in bacteria. *ACS Synth. Biol.* 2, 22–33. doi:10.1021/sb3000657.

Mody, R. S., Heidarynejad, V., Patel, A. M., and Dave, P. J. (1990). Isolation and characterization of

Serratia marcescens mutants defective in prodigiosin biosynthesis. *Curr. Microbiol.* 20, 95–103. doi:10.1007/BF02092880.

Taber, D. F., and Frankowski, K. J. (2006). Grubbs's cross metathesis of eugenol with *cis*-2-butene-1,4-diol to make a natural product. An organometallic experiment for the undergraduate lab. *J. Chem. Educ.* 83, 283–284. doi:10.1021/ed083p283.

Weihmann, R., Domröse, A., Drepper, T., Jaeger, K. E., and Loeschcke, A. (2020). Protocols for yTREX/Tn5-based gene cluster expression in *Pseudomonas putida*. *Microb. Biotechnol.* 13, 250–262. doi:10.1111/1751-7915.13402.

Williamson, N. R., Fineran, P. C., Leeper, F. J., and Salmond, G. P. C. (2006). The biosynthesis and regulation of bacterial prodiginines. *Nat. Rev. Microbiol.* 4, 887–99. doi:10.1038/nrmicro1531.

Williamson, N. R., Simonsen, H. T., Ahmed, R. A. A., Goldet, G., Slater, H., Woodley, L., et al. (2005). Biosynthesis of the red antibiotic, prodigiosin, in *Serratia*: Identification of a novel 2-methyl-3-n-amyl-pyrrole (MAP) assembly pathway, definition of the terminal condensing enzyme, and implications for undecylprodigiosin biosynthesis in <i>Strep. *Mol. Microbiol.* 56, 971–989. doi:10.1111/j.1365-2958.2005.04602.x.
